# NIR Analysis of Intact Grape Berries: Chemical and Physical Properties Prediction Using Multivariate Analysis

**DOI:** 10.3390/foods10010113

**Published:** 2021-01-07

**Authors:** Teodora Basile, Antonio Domenico Marsico, Rocco Perniola

**Affiliations:** Consiglio per la Ricerca in Agricoltura e L’analisidell’Economiaagrarian-Centro di RicercaViticoltura ed Enolgia (CREA- VE), 148-70010 Turi (Ba), Italy; adomenico.marsico@crea.gov.it (A.D.M.); rocco.perniola@crea.gov.it (R.P.)

**Keywords:** NIR, grape, hardness, crunchiness, TSS, iPLS, R software

## Abstract

Texture characteristics are valuable parameters in the perceived quality and overall acceptability of fresh fruit. The characterization of grape texture attributes, such as firmness and crunchiness, is usually performed by sensory analysis or instrumental texture analysis. Both methodologies are destructive. Hence, it is not possible to test multiple times or perform any other analysis on the same sample. In this article, near-infrared (NIR) spectroscopy was applied to intact berries of table grape cv. Regal Seedless. NIR spectra were employed to predict both the physical parameter “hardness”, which is correlated with the crunchiness of berry flesh and the sweetness, which is correlated with the total soluble solids content (TSS, as °Brix). The chemometric analysis was carried out exclusively based on an open-source software environment, producing results readily usable for any operator, besides the specific level of experience with NIR spectroscopy.

## 1. Introduction

### 1.1. Grape Texture Attributes

The production of table grapes with organoleptic properties positively perceived by consumers is a primary objective for grape producers. Texture attributes are of primary importance in the perceived quality and overall acceptability of fresh fruits. According to consumer preference, crunchiness represents a major sensory quality trait of fresh table grape. The assessment of pulp compactness and berry skin consistency is therefore important to predict customer acceptance of the product [1]. Significant effort has been spent in the development of instrumental techniques, involving penetration or puncture and compression tests, which attempt to reproduce the mechanical operations of biting or chewing, to quantitatively determine the mechanical properties of wine grapes and table grapes, according to the sensory texture attributes [2]. The use of a texture analyzer as a surveying analytical technique is often applied in the food sector, for both time and economic reasons, providing more objective and standardized results [3]. In table grapes, typical mechanical parameters instrumentally measured to define the textural quality of whole berry and pulp are hardness, cohesiveness, gumminess, springiness, chewiness, resilience, firmness, toughness, and stiffness, whereas those used to characterize berry skin are hardness, stiffness, and thickness. Hardness, which is the measurement of the force necessary to attain a given deformation was shown to be related to perceived flesh crunchiness in grape berries [4]. The Regal Seedless variety used in this study is a table grape characterized by very large crunchy berries with firm flesh, average sweet taste (berry size 18–20 mm, °Brix 16–18), light green color, and a good shelf life [5].

### 1.2. NIR Spectroscopy

Near-infrared (NIR) spectroscopy is an analytical method suitable for the prediction of both chemical and physical properties of samples, which allows simultaneous qualitative and accurate quantitative analysis of different parameters. One great advantage of this cost-effective and non-destructive analytical method is the ease of sample handling (no sample preparation) and the possibility to analyze samples in any aggregation state. It allows various measurement modes that can be chosen based on the nature of the sample—reflection (for solids, powders, granulate), transflection (for semi-solids, liquids, films, emulsions), or transmission (for clean liquids). An NIR spectrum is composed of overlapping absorption bands related to overtones and combination signals. A large amount of information is hidden in this intricate superimposition of signals. A suitable analysis algorithm is then required to extract the information [6]. A limitation of this technique is the requirement of a large number of samples for a robust calibration of the model and after for maintenance of a calibration routine for each specific application. The prediction of parameters with values out of the training set range is also not possible. NIR spectroscopy is a secondary analytical technique, which requires calibration against a reference method. Both the accuracy and the precision of NIR models are governed by the accuracy and precision of the reference (primary) method (i.e., the reference measured values of the training set) [7].

### 1.3. Prediction Models

NIR spectroscopy has a wide range of agri-food applications. The ability of NIR spectroscopy to measure the quality attributes of fruits and vegetables was shown for many products. Although modern NIR devices were recently developed with attention to their simplification, by integrating user-friendly software for statistical processing and partial automation of analysis, intending to allow less skillful users to routinely perform multiple component quantification, NIR spectroscopy is not a technique without development problems. Indeed, data complexity arising from NIR requires specific statistical analyses and qualified operators [8]. In this article, NIR data were analyzed exclusively with the R software. R is a programming language and a free software environment for statistical computing and graphics. It not only implements a wide variety of statistical and graphical technique with the support of R packages (collections of functions and data sets developed by the community, which are continuously released and improved) but, moreover, gives a researcher tools to create its own functions to perform any statistical analysis [9]. The results of this work show how it is possible to obtain valuable results for predicting the chemical and physical parameters from NIR spectra for difficult samples like intact grape berries, exclusively using an open-source R software, leading to results readily usable for any NIR operator, besides the specific level of experience.

## 2. Material and Methods

### 2.1. Grape Barriers Samples

Regal Seedless grape berries were collected during the 2018 vintage from the experimental vineyards of CREA Research Centre for Viticulture and Enology of Turi, Southern Italy (40° 57′ 26′′ N; 17° 00′ 26′′ E). Three blocks (namely blocks T1, T2, and T3) were identified in different areas of the vineyard. From each block, fifteen bunches were collected at harvest. In the laboratory, berries from each bunch were sorted according to their density by flotation in saline solution, following the procedure described by Rolle et al. [10]. Berries with TSS between 16 and 17 °Brix were collected and subsequently divided according to their equatorial diameter. For each block, three plastic bags containing 50 berries each, were set up. Each plastic bag was covered with a plastic film and placed in a cold store at 0 °C, until the analysis. From each plastic bag, about 30 berries were randomly picked and left at room temperature (20°C) for two hours, prior to the analysis. Each berry was shortly washed with distilled water and gently tapped with paper, prior to NIR measurement. After spectra acquisition for each berry, the Texture Profile Analysis (TPA) was measured, followed by the total soluble solids measurement.

### 2.2. NIR Spectra Measurements

A Bruker TANGO FT-NIR (Fourier Transform Near-Infrared) spectrometer was employed for spectra acquisition. In order to build FT-NIR calibration models for the investigated parameters, after the NIR analysis, hundreds of samples of fresh Regal berries were analyzed with the primary methods. For each berry, the FT-NIR spectra of the intact single berry were recorded in the 12,000–4000 cm^−1^ (833–2500 nm) range on three different berry faces. A background spectrum was automatically recorded, prior to each sample, while both temperature and humidity were kept constant.

### 2.3. Instrumental Texture Analysis

**Figure 1:**
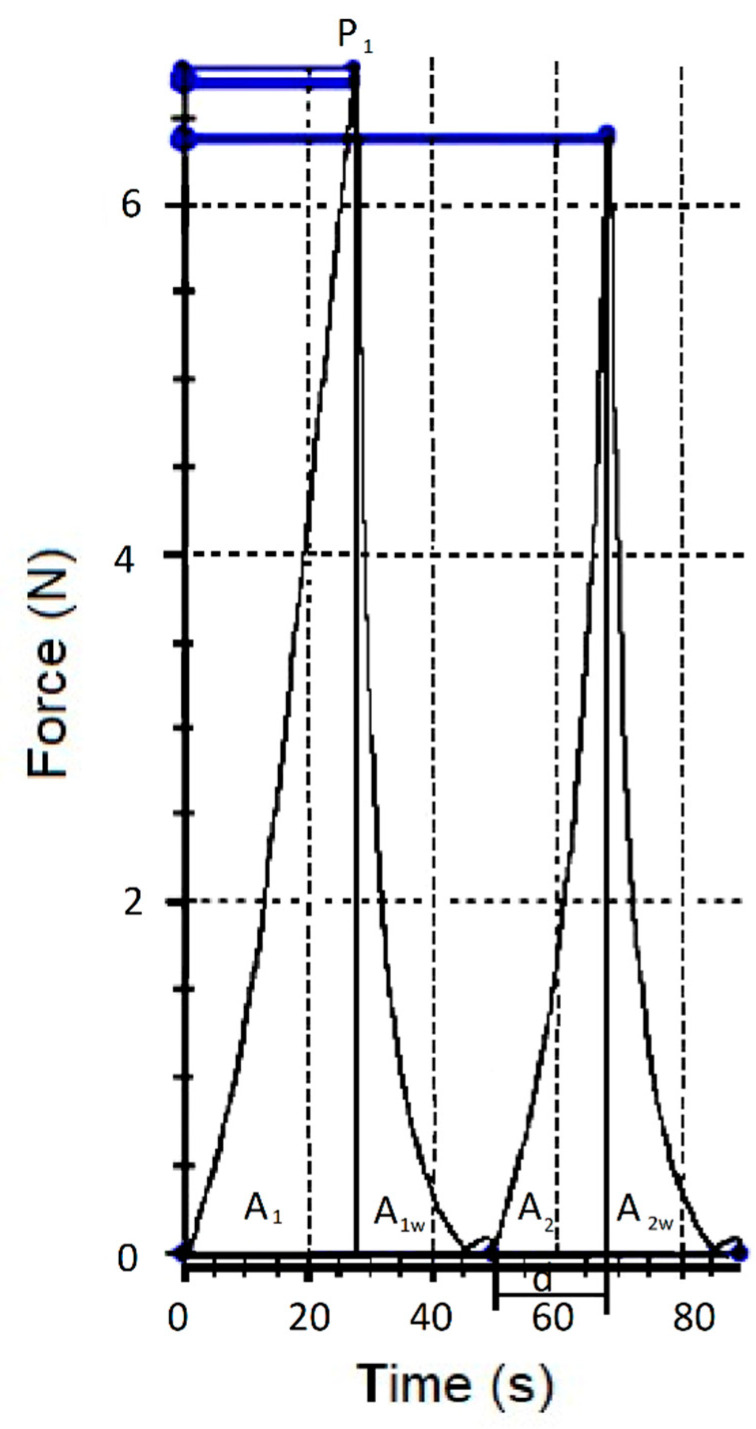
Force-time curves corresponding to the texture profile analysis. P1—force to obtain a deformation of 20% of the equatorial diameter of the berries. A1, A1w, A2, and A2w—areas underpinning specific portions of the force–time curve. d—springiness.

For each berry, the Texture Profile Analysis (TPA) was measured using anXforceP texture analyzer (Zwick/Roell GmbH &Co., Ulm, Germany), equipped with the Zwick Roell software package (testXpert II Zwick/Roell, vers. 3.31 Ulm, Germany). The TPA was obtained by performing on each berry at a double compression test. The berries were placed in their equatorial position on a metal base (first probe) and compressed twice using a 35 mm P/35 flat cylindrical probe (Stable Micro Systems) (second probe), under 20% deformation, with a waiting time between the two compressions of 2 s and a test speed of 1 mm/s. From the force–time curve (Figure 1), values for hardness (N, as P1), springiness (mm, as d), cohesiveness (adimensional, (A2 + A2w)/(A1 + A1w)), gumminess (N, as hardness*cohesiveness), and chewiness (mJ, as gummines*springeness) were automatically calculated by the software [11]. The equatorial diameters of the berry were also provided by the software as the distance between the two probes when the second probe touched the surface of the berry.

### 2.4. Soluble Solids Measurements

After spectra acquisition and double compression tests, the total soluble solids content (TSS, °Brix) was measured in triplicates at 20 °C, using a digital refractometer Atago PR1 (Atago Co., Tokyo, Japan).

### 2.5. Statistical Analysis

All statistical procedures described in detail in the following paragraphs, including pre-treatments of the original spectra, outlier detection (principal component analysis, PCA), calibration, cross validation, and external validation of prediction models (partial least-squares PLS and interval partial least-squares iPLS regressions), were performed using the open source R statistical software. R version 3.5.3 (11 March 2019) Copyright © 2021 The R foundation for Statistical Computing, Venna, Austria [9]. The R packages used were—mdatools [12], signals [13], ggbiplot [14], and ClassDiscovery [15].

## 3. Results and Discussion

### 3.1. NIR Spectra Pretreatments

**Figure 2:**
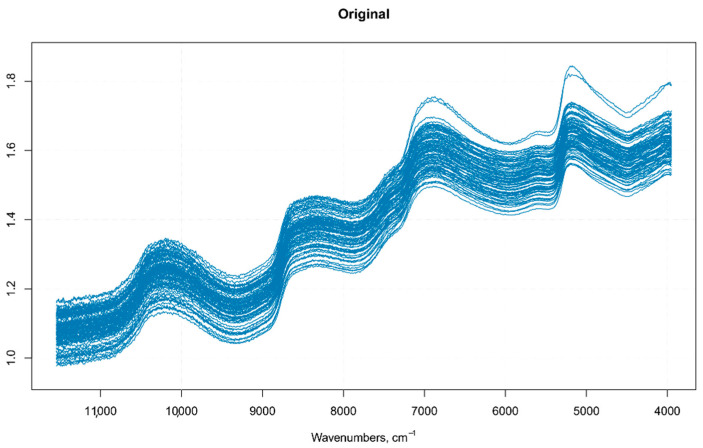
Raw spectra of single Regal seedless berries.

**Figure 3:**
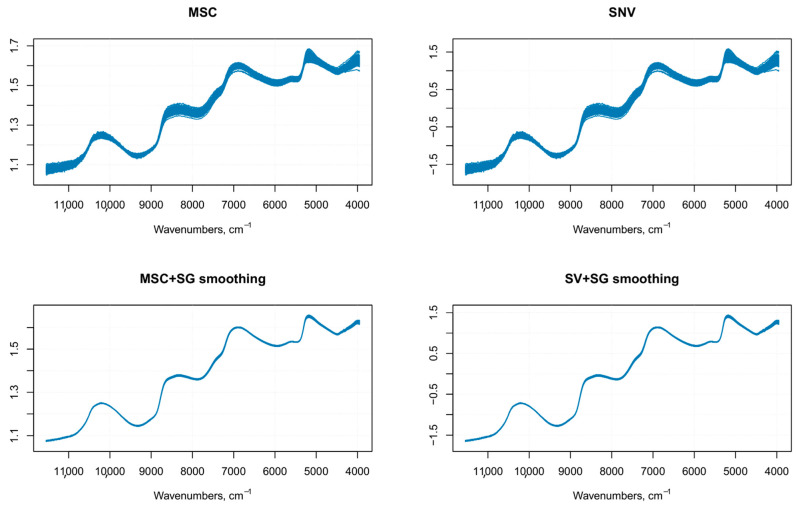
Results of spectral data pretreatment using multiplicative scatter correction (MSC) and standard normal variate (SNV) with (bottom spectra) or without (top spectra) smoothing.

**Figure 4:**
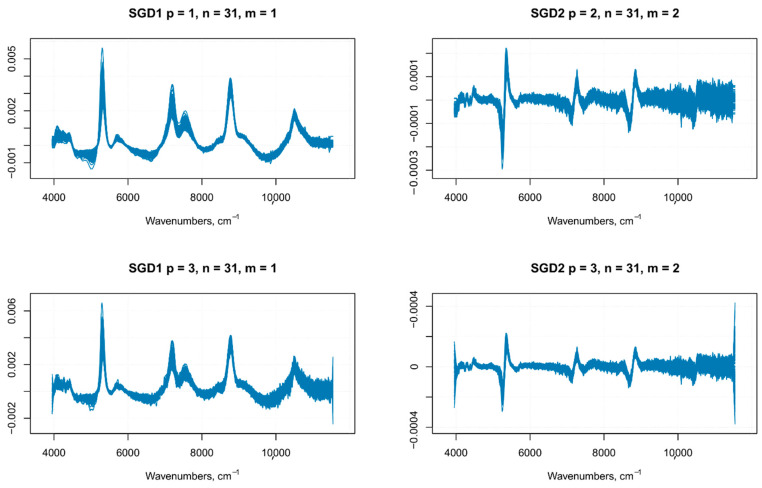
SGD1 and SGD2 with different polynomial order (p) and frame size (m).

Differences in the physical nature of the samples such as the non-homogeneous distribution of particles, particle size differences, density variations, morphology (shape and roughness) differences of the sample surface, result in sample-to-sample variations in the overall path-length the photons have to travel before reaching the detector. Even small physical differences lead to light scattering effects that influence the NIR spectra and result in baseline shifts and scaling variations (intensity variations) [16]. Therefore, it is common in NIR analysis of solid samples recorded in the reflectance mode to find variations in spectral intensity, light scattering effects, path length variations, and baseline shifts in the spectra due to particle size or particle size distribution, which together with other causes of variations (instrumental effects such as random noise, changes in lamp intensity, and detector response) can adversely affect the robustness and reliability of the analysis [17,18,19]. These alterations in the spectra can be detrimental to subsequent quantitative analysis, leading to inaccurate predicted results. The pre-treatment of NIR original raw data is, therefore, the first step prior to any model development and optimization [16]. An optimal pre-treatment helps to eliminate interferences and facilitates information extraction from NIR spectral data, allowing the building of a prediction model from a regression analysis, characterized by the lowest statistical error [20]. There are several pre-processing methods available for NIR spectra. The choice of the (or the combination of) specific pre-treatment that can or should be applied requires an appropriate evaluation of the quality of the raw data to be analyzed (e.g., baseline shifts, presence of nearby peaks, poor spectral resolution, and so on) and the software that is used for data analysis [17]. In this article, the pre-treatments were applied to the raw spectra using the library “signal” [13]. The pre-processing techniques usually employed on NIR spectra, belong to two main categories—scatter-correction methods (which are used to remove undesired spectral variations due to light scatter effects and variations in effective path length) and spectral derivatives (useful to emphasize pronounced, but small features, e.g., steep edges of a peak, over a broad background, at the cost of increasing noise) [21]. In this study, the pretreatment methods tested on the NIR raw spectra (*n* = 270) (Figure 2) were multiplicative scatter correction (MSC) with or without smoothing, standard normal variate (SNV)with or without smoothing (Figure 3), first order Savitzky–Golay derivative (SGD1), second order Savitsky–Golay derivative (SGD2) (Figure 4), and their combination. Both MSC and SNV pre-treatments were able to remove a large part of the variance from the original spectra without distorting the spectral features.

There is no unified method to define the parameters of the Savitzky–Golay filter, such as the polynomial order, frame size, and the derivative order; usually, the choice is based on a trial and error method. In this work, the multiple set of parameters employed was based on previous data on similar samples, which were then employed on our samples as the starting point for a further optimization of the Savitzky–Golay filter parameters, as explained in the following lines. In a previous article on grape juice, the best prediction model for TSS (°Brix), anthocyanins and total polyphenols was built with NIR spectra pre-treated with an SGD1, with a window size of 15 (with or without SNV) [22]. Even if the nature of our samples is quite different (i.e., the whole berry), we used this combination of parameters as a starting point to estimate the optimal parameters of a Savitzky–Golay smoothing filter. The purpose of smoothing is to get rid of random noise while (ideally) preserving the true spectral signal, therefore, the optimal choice of smoothing parameters allows having a reasonable estimate of the signal from the noise. After the application of filters with different parameters, the noise level was evaluated on the portions of the spectrum thatwere devoid of important features (i.e., no peaks). If the noise increasedin those regions of the spectrum, the combination of parameters used in the filter was discarded (Figure 4 shows a comparison of SGD1 and SGD2 with different parameters). The optimal parameters chosen were—first polynomial order and 31 frame size for the first derivative, and third polynomial order and 31 frame size for the second derivative.

**Figure 5:**
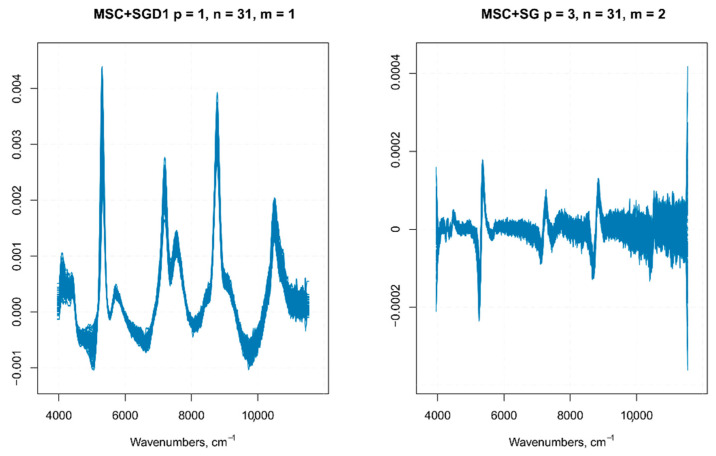
MSC followed by first order Savitzky–Golay derivative (MSC + SG1D) and MSC followed by second order Savitzky–Golay derivative (MSC + SG2D).

Since the order of combined pre-treatments leads to different results [23], and the Savitzky–Golay smoothing method allowed eliminating noises like baseline-drift, tilt, reverse, and so on [24,25], therefore, the Savitzky–Golay filter was applied after SNV or MSC treatments. It is known that MSC and SNV produce very similar resultsfor most practical applications, especially when the average spectrum of the calibration set is used as the reference spectrum in MSC [21], as it was performed on our spectra.A comparison of SNV and MSC pre-processed spectra gave similar results, as shown in Figure 3. Since these pre-treatments are closely related and the difference in prediction ability between the two methods is very small, as shown by other authors [18], therefore, we used only MSC as the scatter-correction method, prior to the derivatization. The following combinations of pre-processing methods were performed on the raw spectra—MSC followed by Savitzky–Golay smoothing, MSC followed by first-order Savitzky–Golay derivative (MSC + SG1D), MSC followed by second-order Savitzky–Golay derivative (MSC + SG2D) (Figure 5).

### 3.2. NIR Spectra Analysis: Principal Component Analysis

**Figure 6:**
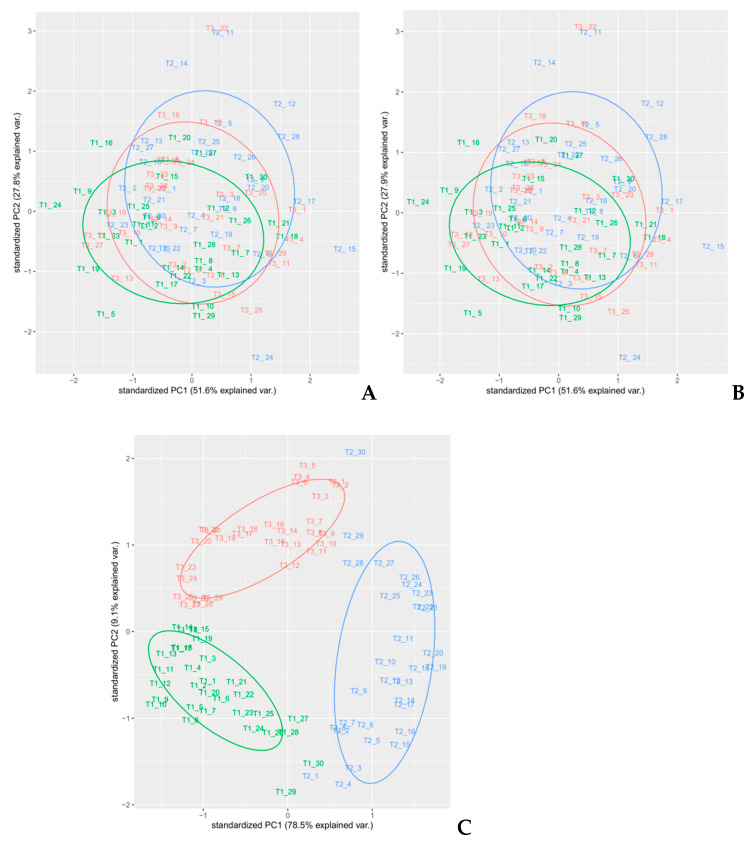
PCA analysis of spectra with different pre-treatments—(**A**) MSC, (**B**) SNV, and (**C**) MSC with smoothing.

**Figure 7:**
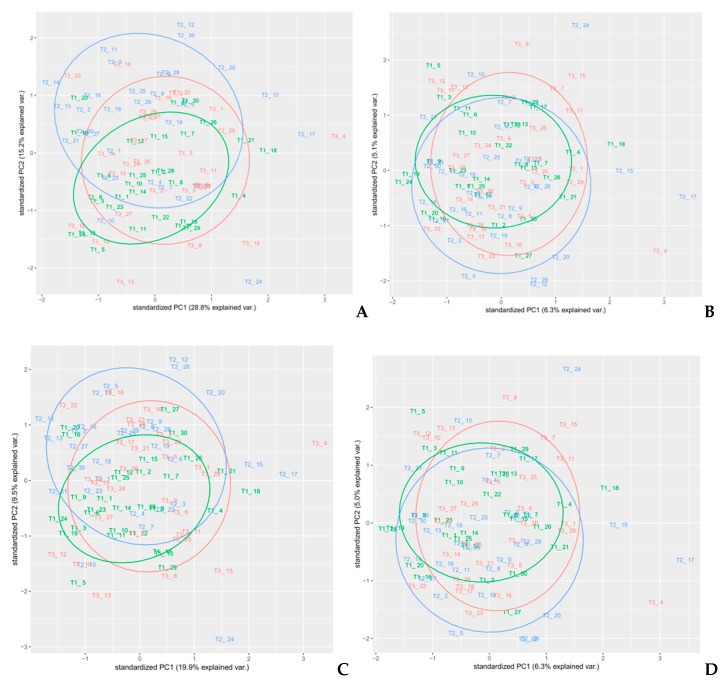
PCA analysis on spectra pre-treated with Savitzky–Golay derivatives. (**A**) D1 (*p* = 1, *n* = 31, *m* = 1), (**B**) D2 (*p* = 2, *n* = 31, *m* = 2), (**C**) D1 (*p* = 3, *n* = 31, *m* = 1), and (**D**) D2(*p* = 3, *n* = 31, *m* = 2).

**Figure 8:**
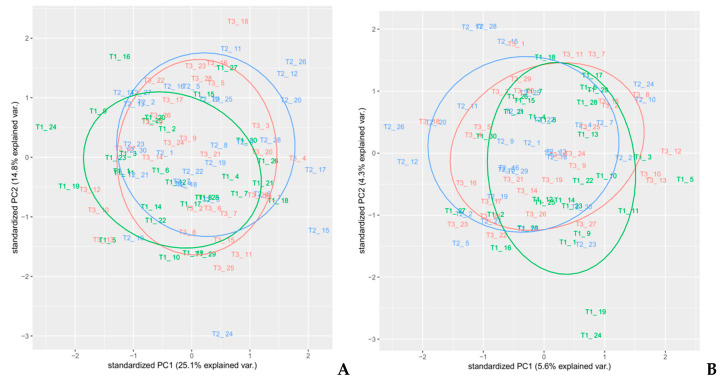
PCA analysis—(**A**) MSC+SGD1 (*p* = 1, *n* = 31, *m* = 1), and (**B**) MSC+SGD2(*p* = 3, *n* = 31, *m* = 2) pre-treatments.

In order to select an appropriate pre-treatment, PCA analyses on each pre-treated set of spectra were performed. Different colors used for the samples reflected the randomized block design (namely blocks T1, T2, and T3). PCA is a well-known unsupervised method often used for dimensionality reduction of multivariate data [26] that is able to highlight common features that allow to group samples with similar composition and thus underlying attributes, as shown in previous works [27,28]. In this article, a PCA analysis was performed on all spectral regions of each different pre-processed spectra as a preliminary exploratory tool, in order to verify the clustering of samples, to compose calibration and external validation groups, and identify outliers (Figure 6, Figure 7 and Figure 8). PCA was performed using the built-in R functions princomp, which uses the Singular Values Decomposition (SVD) method to compute PCA loadings. The pre-processing method in PCA analysis that resulted in the best discrimination of samples was MSC, followed by smoothing, where two principal components explained 87.6% of the total variance of the data; Table 1. In Figure 6C, a distinct separation of the sample in the plot that reflected the experimental design (randomized block) was observed. Therefore, we selected MSC as the pre-treatment to use on the spectra, since the cumulative amount of variance explained by the first two PCs for MSC pre-treated spectra was also high (79.4%), without any net separation of the different groups of samples, based on the experimental design. The loadings on the two PC’s for the MSC pre-treated spectra are reported in Supplementary Materials Table S1.

After the identification of MSC as the optimal pre-treatment for our data, to ensure the reliability of the dataset, it was necessary to identify and remove the outliers. The presence of outliers is dangerous since even a small percentage of them can distort the results and lead to a misleading outcome. Outlier detection for high dimensional data becomes more complex for multivariate data. PCA could be used as an outlier detection technique. Since this algorithm is computationally fast and robust in detecting outliers in multivariate data, it is able to detect multiple outliers in high dimensional datasets [29]. Another way to detect outlier is the calculation of the Mahalanobis distance, which is commonly used to determine multivariate outliers. Not only was PCA used to classify the spectral data and determine outliers, but moreover, the outlier’s detection was performed computing p-values associated with the Mahalanobis distance for each sample using the ClassDiscovery package [15] Therefore, we compared the results of PCA and Mahalanobis distance computation to find outliers in our dataset. Spectra named T1_5, T1_24, T2_11, T2_12, T2_14, T2_15, T2_24, and T3_22 were identified as the sample outliers.

### 3.3. Texture Data

#### 3.3.1. Multivariate Calibration and Validation on “Hardness” Data

**Figure 9:**
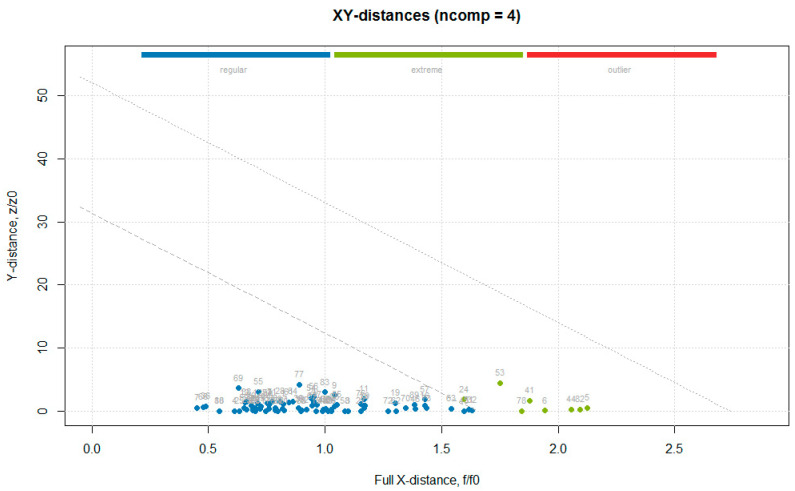
Residual (distance) plot with normalized orthogonal and score distances of the “hardness” model for cross-validated results built using a robust approach for computing critical limits (indicated by the dotted lines).

The data obtained from the primary methods for the test set (Table S2 in Supplementary Materials) were used together with the absorbance values for the entire wave number axis (1899 values), to build a data frame used in the following analysis. The multivariate calibration was performed using the partial least squares (PLS) regression, which uses the so called “PLS components” that are determined to maximize the covariance between the spectral data (independent variables) and the concentrations (dependent variables), to create multivariate models using a linear combination of spectra wave numbers with a given property of interest [20]. The PLS analysis was performed using the mdatools package, in which the only algorithm implemented for PLS analysis was SIMPLS [12]. The optimal number of components couldbe automatically selected using the RMSE values calculated for the different number of components and through cross-validation predictions. In order to detect and remove outliers prior to obtaining the final PLS model, the procedure followed was the one described in Rodiova et al. (2020) [30]. The optimal number of principal components for the PLS model was determined by performing a leave-one-out cross-validation procedure. The mdatools package implemented several methods for computing the critical limits for residual distances, a robust approach was first used, since using classical estimators when data are contaminated with outliers might lead to wrong estimators, therefore, in this case it was recommended to instead use a robust version. The robust approach utilizes the median and inter-quartile range instead of mean and standard deviation, and is insensitive to small and larger deviations [31]. After the outlier’s detection and removal step, a classic approach, namely the data driven approach, based on classical estimators (statistical moments), is used to build the final model [32]. Although some samples were detected as outliers with the PCA analysis, none of them was detected as an outlier in the PLS. Some of the PCA outliers were found among the samples identified as extremes in the residual plot (Figure 9: 5 is T1_5, 6 is T1_6, 41 is T2_11, 44 is T2_14, 53 is T2_23, 78 is T3_18, and 82 is T3_22).

**Figure 10:**
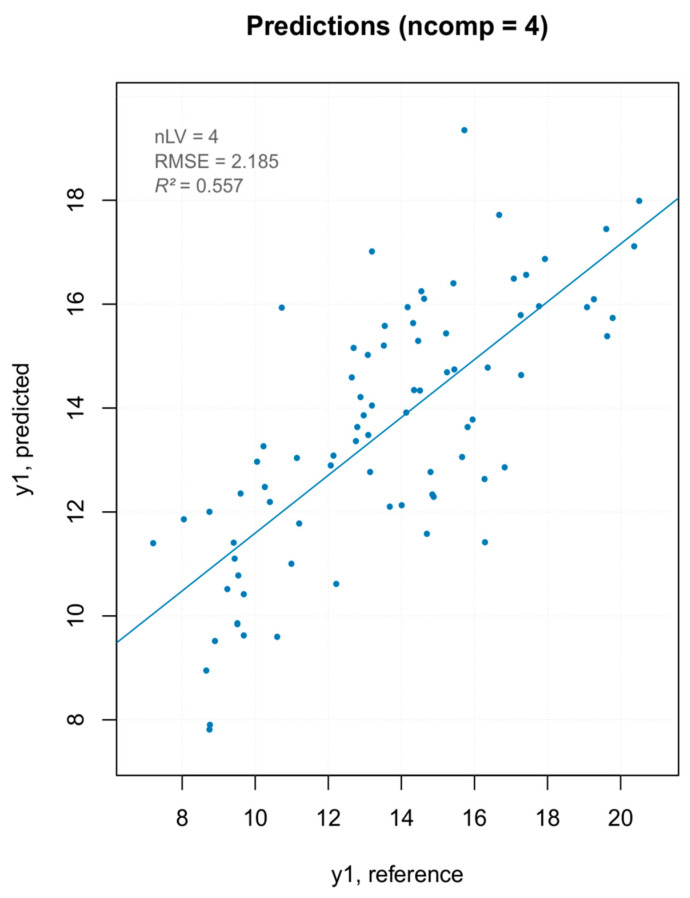
PCA outlier removal—prediction plot for the final model for “hardness” with performance statistics.

**Figure 11:**
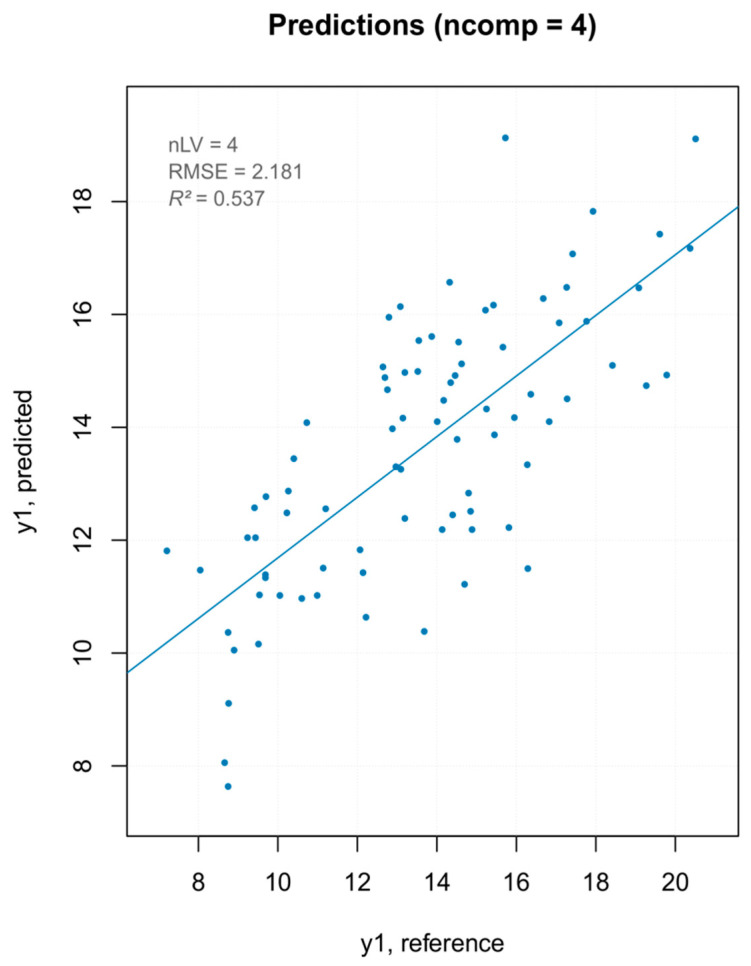
Extreme values removal—prediction plot for the final model for “hardness” with performance statistics.

Three PLS models were built—one with all MSC pretreated spectra, one removing the PCA outliers (Figure 10), and one removing the extreme values (Figure 11). The model built removing the PCA detected outliers gave the best results in terms of prediction capability, which was evaluated by the root mean square error (RMSE), coefficient of determination (R^2^), bias (average difference between the NIR-predicted values and the measured or reference ones) and Residual Prediction Deviation (RPD) for calibration and cross-validation (Table 2). The number of factors (called “ranks”) is a very important parameter to evaluate the quality of a regression model. A low rank implies that the model had a higher stability due to noise reduction. The three low-rank models (number of component 4) indicated an effective removal of sparse noise from the NIR signal [33]. The coefficient of determination gave the percentage of variance present in the component values, which was reproduced in the prediction. A low *R*^2^ means an insufficient precision of the reference data or the presence of outliers in the calibration dataset. Due to the intrinsic complex nature of the solid samples, generally accepted good *R*^2^ values for solid samples are lower than those for liquid samples (optimum was 90%). [34] Even so, the values shown for PLS models were below 50%, describing models of insufficient quality. Both RMSE for calibration and cross-validation were calculated for the three models. Not only was there a very small difference in the RMSE values among the models but, moreover, even the smallest one was over 2, which reflected the poor ability of the model to precisely predict the hardness parameter. The library used to build the PLS model computed the RPD (the standard deviation of observed values divided by the Root Mean Square Error of Prediction (RMSEP)). The RPD takes both the prediction error and the variation of observed values into account, providing a metric of model validity that is more objective than the RMSEP and is more easily comparable across model validation studies. The higher the RPD, the better the model’s predictive capacity. Even though some authors established RPD values higher than 2 for satisfactory calibration models for prediction purposes, the values ranging between 1.4 and 2.0 were indicative of fair models [35], and the interpretation of the RPD was somewhat arbitrary with different thresholds for a good model used in the literature [36]. However, its systematic use was criticized by several authors, since using the standard deviation to represent the spread of a variable can be misleading on a skewed dataset [37]. As bias is defined as the average difference between the NIR-predicted value and the real value, it should be close to zero. Therefore, a positive value means that, on average, the model over-estimated the composition of this amount, while a negative value represented an underestimation. It must be underlined that there are no acceptable values for any of the parameters listed above. They are better interpreted and applied comparatively, rather than absolutely. The nature of the dataset (e.g., disperse values, high standard deviation of measured data, and so on) greatly affect the quality of the predicted outcome. For the PLS regression, however, even the best regression model, in terms of *R*^2^ and RPD, showed that the data points did not fit well with the statistical model (low *R*^2^) and were not able to accurately predict the reference values (RPD close to one), with a slight underestimation of the actual values (negative bias).

#### 3.3.2. iPLS on “Hardness” Data

**Figure 12:**
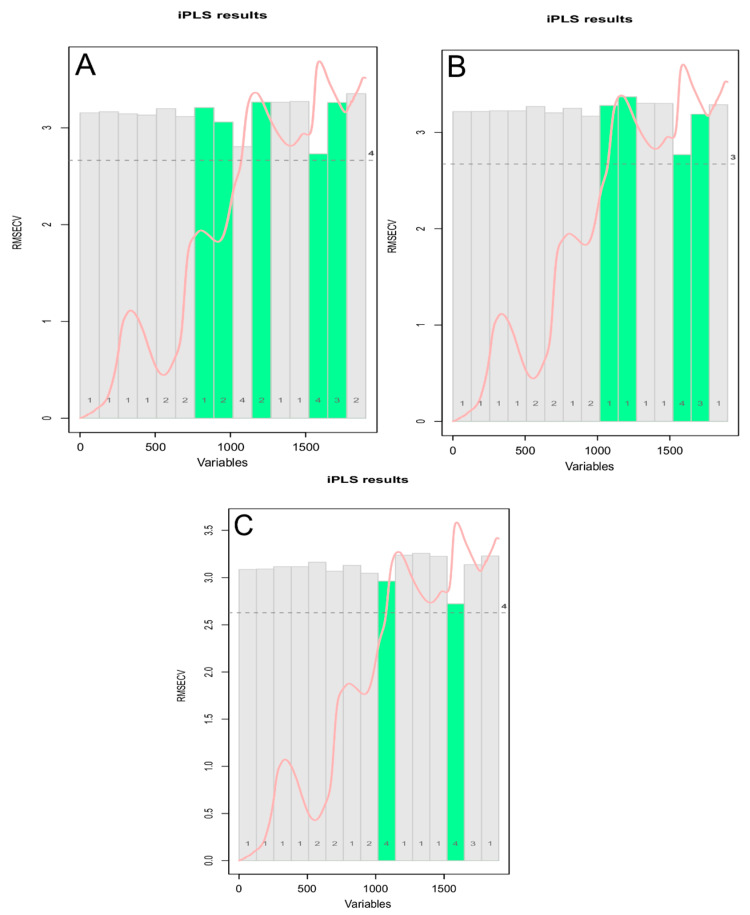
Selected intervals (in green) for iPLS models for “hardness” data—(**A**) for all spectra of the training set, (**B**) after PCA outliers removal, and (**C**) after extreme spectra removal.

It was shown how a large number of variables in a dataset often results in an unreliable prediction of the dependent variable. Chemometrics can be used for the selection of a small number of relevant variables, which represent the most useful information contained in the full spectra. In order to extract this useful information from the NIR spectra, different variable selection methods could be performed [38]. Therefore, in order to improve the predictive performance of the regression model, an interval partial least-squares (iPLS) regression was implemented. The iPLS is a modelling procedure for variable selection method, mostly used on spectroscopic data in which different intervals of the spectral wavenumbers and their combinations are used to find the most relevant one for prediction of a response variable [39]. The iPLS models were built (mdatools library) with a forward iterative stepwise selection procedure on 15 intervals and a ten-fold venetian blind cross validation—one using all spectra, one removing the PCA outliers, and another one removing the extreme samples.The iPLS variable selection results is shown in Figure 12, where the bars represent the 15 intervals (the selected intervals are the 5 green ones), the height of each bar corresponds to the RMSECV value for the local model made using variables from this interval as predictors (first iteration), and within each bar, the number of PLS components used in the local modelis reported. The mean spectrum is also reported in red, while the dashed line shows error for the global model. The optimal number of principal components for the iPLS models was determined using an automatic selection based on the “Wold criterion”, which is based on a ratio between the PRESS values for the current and the next component.

**Figure 13:**
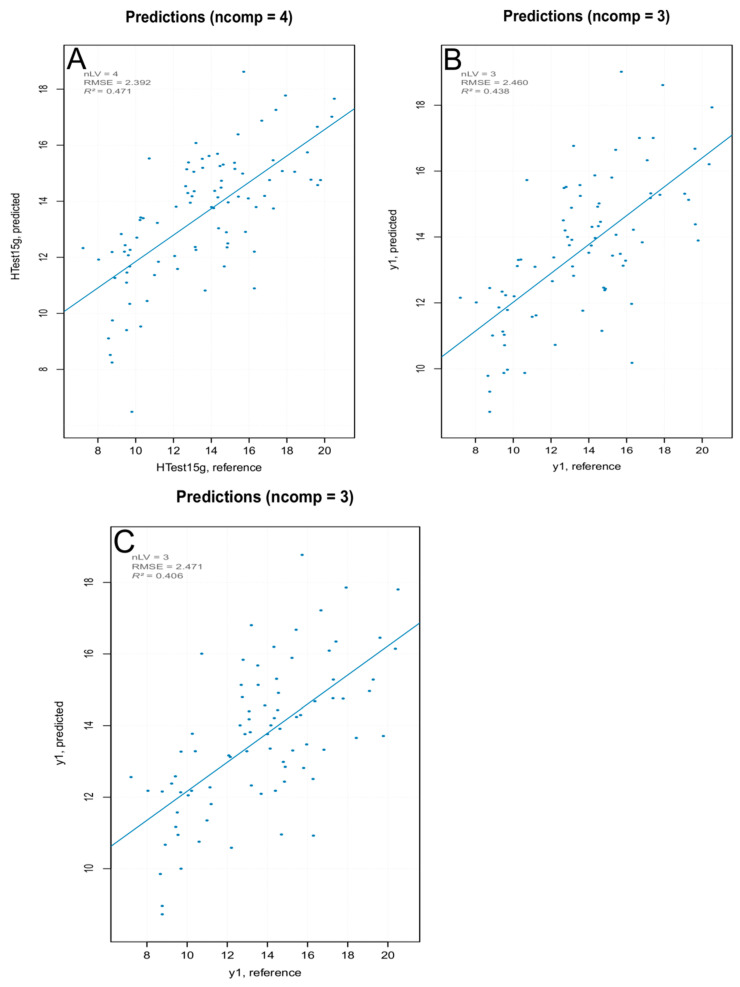
iPLS prediction models for (**A**) all spectra of the training set, (**B**) after PCA outliers removal, and (**C**) after extreme spectra removal.

The iPLS models built using selected interval of wavelengths showed more satisfactory parameters compared to the PLS models (Table 3 and Figure 13). The best predictive resultswere obtained with an iPLS model (Table S3) built using all spectra in the training set, with an increased fitting of the experimental data on the statistical model (*R*^2^ 0.44 for the best iPLS model compared to 0.32 for the best PLS model), better prediction of the parameter (higher RPD: 1.30 for the best iPLS model compared to 1.23 for the best PLS model), with a slight overestimation of the actual values (positive bias).

**Figure 14:**
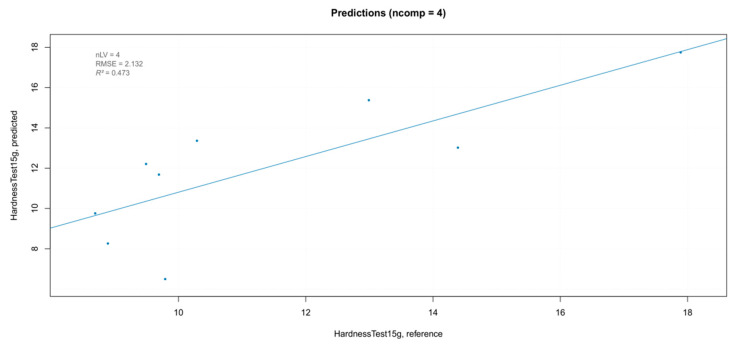
Results of the external validation for the “hardness” parameter.

In order to estimate the overall prediction ability of the best iPLS model, it was validated with a new set of samples. The samples in the test set were berries belonging to the three blocks of the experimental randomized design, which were not used in the calibration of the model, with “hardness” values included in the range of those of the training set. The predicted values are shown in Figure 14. The overestimation of the prediction model was more evident for the lower values of hardness. This could be attributed to the small numerosity of berries with the smaller hardness values in training the dataset, which resulted in a smaller contribution to the model.

Both the accuracy and precision of a model obtained from the NIR data are strictly linked to the accuracy and precision of the primary reference method used [7]. This meant that low precision and or accuracy in the reference data strongly affect the prediction model efficacy. For any quantitative measure, the knowledge of the uncertainty associated with the measurement is essential for a correct interpretation of the results. Without uncertainty information, results could be misinterpreted (e.g., observed differences are not only linked to the experimental variability), which in turn, might lead to an incorrect evaluation of the analysis outcome [40]. The precision of the instrumental measure of “hardness” was determined for the densimetric-sorted berries (i.e., berries belonging to the same density class). The standard deviation range of the measured values (SD 0.2–3.8) was consistent with the values usually associated with the measure of this parameter using compression testing machines as the primary reference methods by other authors (SD ranging from 0.7 to 3.2) [4]. Such high uncertainty values associated with the reference method negatively affected the NIR analysis outcome. Usually, when dealing with primary methods with low precision or accuracy, the best choice is to use another reference method. Unfortunately, all methods used for texture analysis (sensory analysis and instrumental texture analysis) are affected by strong uncertainty. Therefore, when evaluating the NIR outcome, we must consider the nature of the input data used. The most commonly used statistical index that accounts for the model reliability is the residual predictive deviation, which evaluates the predictive accuracy. For the best iPLS prediction model, RPD was 1.30, which indicated a fairly accurate model [35]. These results can be considered very good for the nature of our dataset. The RPIQ (residual predictive interquartile amplitude, IQ/SECV) is another index able to evaluate the predictive ability of the calibration models. It indicates that the calibration models are satisfactory for prediction purposes when RPIQ > 2 [37,41]. For the best “hardness” model the RPIQ was >2 therefore, according to this criterion, the calibration model developed for the “hardness” parameter on the whole berries could be considered as good ones for the prediction of hardness values from unknown samples.

### 3.4. TSS Data

#### 3.4.1. Multivariate Calibration and Validation on TSS Data

**Figure 15:**
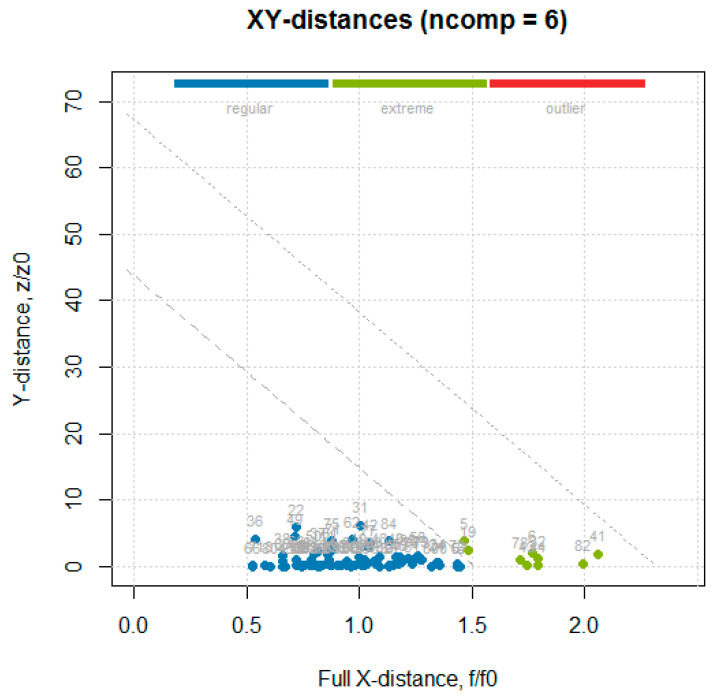
Distance (residual) plot with normalized orthogonal and score distances of the TSS model built using a classic approach for computing the critical limits.

The procedure described in the previous paragraphs for the parameter “hardness” was also followed for the TSS data. Since no outliers were detected with the robust approach (Figure 15), the classic data driven approach was used to build the model and the optimal number of components was automatically selected.

**Figure 16:**
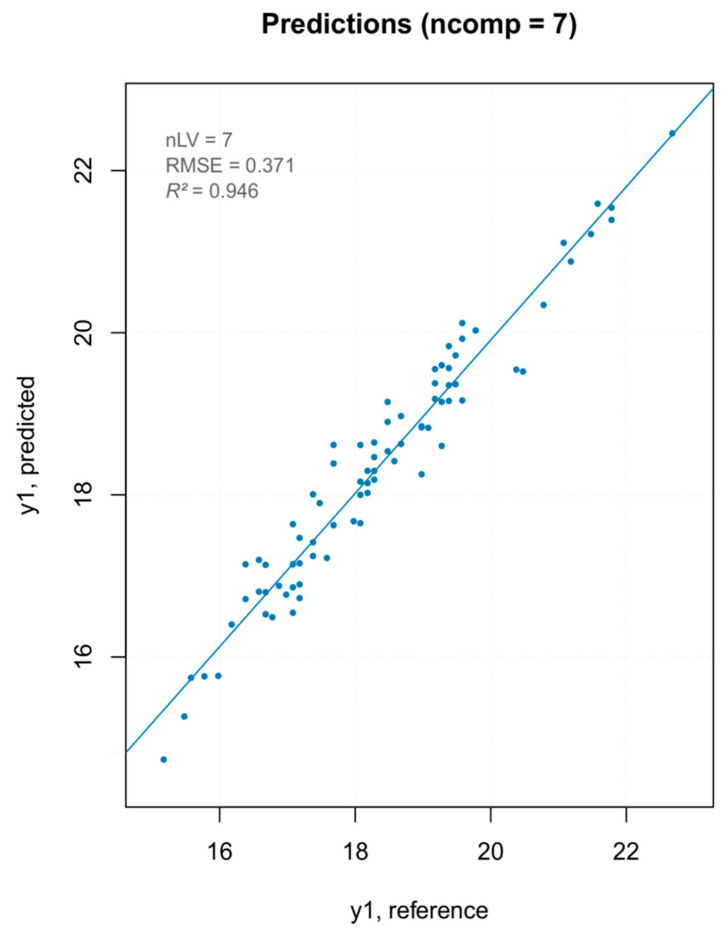
Prediction plot for the final TSS model.

Two prediction models were built, one using all spectra and another byremoving the PCA detected outliers (Table 4 and Figure 16). Since the removal of the extreme values did not lead to any improvement in the prediction model, those spectra were not removed.

#### 3.4.2. iPLS on TSS Data

**Figure 17:**
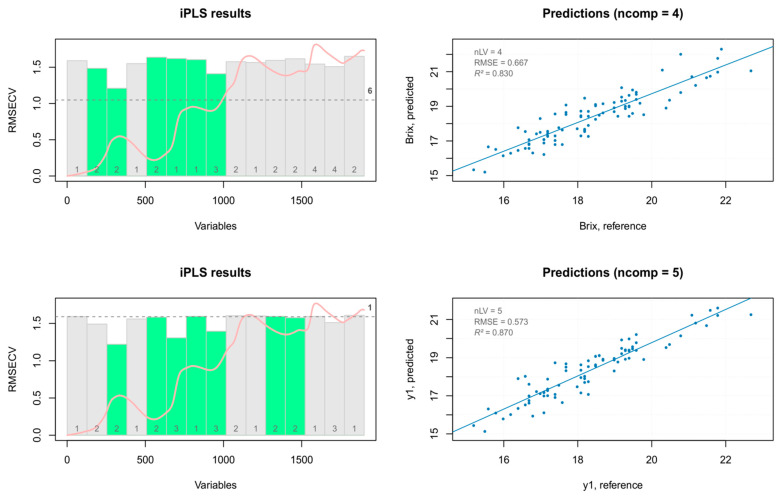
iPLS selected intervals (**left**) and iPLS prediction models (**right**) for TSS data.

The TSS prediction models built with PLS regression were improved in terms of both accuracy and predictability, using the iPLS analysis (forward method) with venetian blinds validation on 15 intervals. The iPLS variable selection results are reported in Figure 17. The five components iPLS model obtained after PCA outliers removal using seven selected intervals, showed a better prediction capability. Indeed, the best regression model showed a good fit of the experimental data (*R*^2^), it was able to accurately predict the reference values (RMSEP), and had a high RPD (RPD >2 indicate satisfactory calibration models for prediction purposes) [35] with a good estimation of the values (bias close to zero) (Table 5).

**Figure 18:**
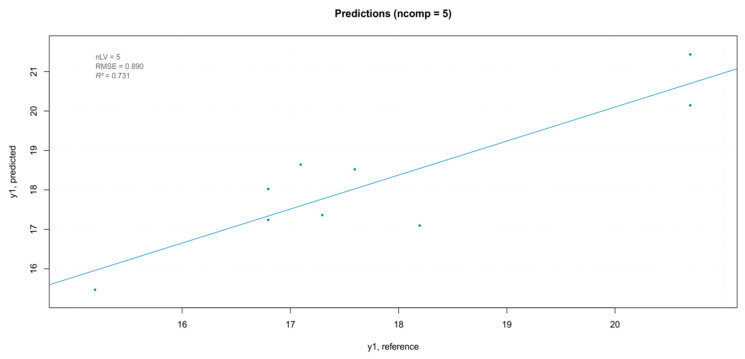
Results of the external validation for the TSS model.

The iPLS model showed a better predictability, and thus was used for the external validation on the same test set used for the “hardness” parameter. The model built with the external validation set showed a good fit (Figure 18). As reported in previous articles, NIR technology is not appropriate for the measurement of internal qualities in fruits with thick skin (e.g., passion fruit) or heterogeneous internal structure (e.g., tomatoes). This result was shown for fruit quality attributes such as sugars or acids, which are routinely measured with high accuracy using NIR spectroscopy on other fruits [42]. Our results conform with these findings, showing a difference between the NIR-predicted values and the measured values. This result could be mainly attributed to the low depth of penetration of the NIR radiation, which is more evident on the solid non-homogeneous samples.

## 4. Conclusions

The objective of this research was the analysis of intact table grape berries in terms of both texture-related (crunchiness as textural hardness) and chemical-related (sweetness as TSS) parameters, using NIR spectroscopy. These specific parameters were investigated due to their importance in customer’s acceptance of fresh table grape. Standard analysis techniques for texture characteristics of fresh fruits are destructive ones, which prevents any further analysis of the sample. NIR spectroscopy is a non-destructive methodology often used for fruit and vegetables. In NIR analysis, a change in concentration of an NIR-active compound modifies the spectrum, allowing to correlate the amount of that compound (or a class of structurally similar compounds, e.g., sugars) to the modification observed. Indeed, NIR analysis shows an optimal prediction for parameters linked to chemical compounds present in the sample. The prediction of properties not directly connected to a chemical compound or a class of compounds but rather to a combination of different components is not simple. This difficulty increases when dealing with non-homogenous samples such as intact fresh fruit samples. Our results show that it is possible to obtain information on texture-related parameters on fresh fruits, using adequate chemometric techniques. NIR spectra were recorded in the spectral region of 12,000–4000 cm^−1^ (833–2500 nm), using a set of hundreds of berries. Several pre-treatments of the original spectra were applied to the raw spectra and the best pre-treatment (i.e., MSC) was selected based on the amount of variability explained in a PCA analysis. Moreover, outlier samples in the PCA were identified based on the Mahalanobis distance. A PLS analysis was applied to MSC pre-processed spectra using different training sets—all recorded spectra, after the removal of those recognized as outliers in the PCA or removing the spectra that were extreme, by considering the PLS critical limits. To improve the predictability of the models, interval partial least square (iPLS) models were also built on the same three training sets. The predictability of the best iPLS model was confirmed by external validation by an independent test set. The same procedure was followed for both texture and chemical data. The NIR analysis of the investigated texture parameter provided models with less satisfactory performance statistics compared to the TSS one. Given the accuracy and precision of the reference method employed, the regression model was able to satisfactorily predict the “hardness” parameter (residual predictive interquartile amplitude, RPIQ > 2). According to our results, NIR technology appears to be a promising technique for both predicting the compositional parameters and obtaining information concerning the physical parameters in table grape, which would allow the application of efficient and low-cost instruments for the fruit industry. Both PLS and iPLS analysis are widely used in NIR analysis to search for the linear relationships between the measured and predicted values. To achieve a better prediction for the physical parameters, other types of relationships could be investigated. Ongoing studies are evaluating the predictive ability of non-linear models (e.g., as artificial neural networks, ANN) with newly released R packages.

## Figures and Tables

**Table 1:** PCA analysis results of spectra with different pre-processing techniques.

Pretreatment	Parameters	PC1%	PC2%
MSC	-	51.6	27.8
SNV	-	51.6	27.9
SGD1	1 31 1	28.8	15.2
SGD2	3 31 2	6.3	5
MSC+SG smoothing	-	78.5	9.1
MSC+SGD1	1 31 1	25.1	14.8
MSC+SGD2	3 31 2	5.6	4.3

**Table 2:** PLS model summary for “hardness” data.

Data Set	Model	X Cumexpvar	Y Cumexpvar	*R* ^2^	RMSE	Slope	Bias	RPD
Training set (all spectra) nComp = 4	Cal	88.88688	50.56211	0.506	2.312	0.506	0.000	1.43
	Cv	NA	NA	0.305	2.741	0.383	−0.0005	1.21
PCA outlier removal nComp = 4	Cal	84.40549	55.65603	0.557	2.185	0.557	0.0000	1.51
	Cv	NA	NA	0.327	2.692	0.423	−0.0468	1.23
Extreme values removal nComp = 4	Cal	86.99243	53.71146	0.537	2.181	0.537	0.000	1.48
	Cv	NA	NA	0.325	2.633	0.411	0.028	1.22

Number of selected components for all models—4; Cross-validation—full (leave-one-out); NA—not available.

**Table 3:** iPLS models for the “hardness” parameter.

Data Set	Model	X Cumexpvar	Y Cumexpvar	*R* ^2^	RMSE	Slope	Bias	RPD
All training set nComp = 4	initial global model	98.47121	47.09408	0.471	2.392	0.471	0.0000	1.38
	final model	NA	NA	0.405	2.536	0.440	0.0176	1.30
PCA outlier removal nComp = 3	initial global model	76.11868	43.80306	0.438	2.460	0.438	0.0000	1.34
	final model	NA	NA	0.391	2.561	0.402	−0.0120	1.29
Extreme values removal nComp = 3	initial global model	86.49316	40.5588	0.406	2.471	0.406	0.0000	1.31
	final model	NA	NA	0.376	2.531	0.394	0.0412	1.27

nComp—number of selected components; NA—not available.

**Table 4:** PLS model (class pls) summary for the TSS data.

Data Set	Model	X Cumexpvar	Y Cumexpvar	*R* ^2^	RMSE	Slope	Bias	RPD
Training set (all spectra)nComp = 6	Cal	91.90354	83.1635	0.832	0.663	0.832	0.0000	2.45
	Cv	NA	NA	0.611	1.008	0.621	0.0158	1.61
PCA outlier removal nComp = 7	Cal	91.34024	94.56569	0.946	0.371	0.946	0.0000	4.32
	Cv	NA	NA	0.619	0.982	0.598	0.0415	1.63

nComp—number of selected components; Cross-validation—full (leave-one-out); NA—not available.

**Table 5:** iPLS models for TSS data.

Data Set	Model	X Cumexpvar	Y Cumexpvar	*R* ^2^	RMSE	Slope	Bias	RPD
Training set (all spectra) nComp = 4	Cal	95.33922	82.98385	0.830	0.667	0.830	0.0000	2.44
	Cv	NA	NA	0.659	0.943	0.615	0.0145	1.72
PCA outlier removal nComp = 5	Cal	95.69764	87.0354	0.870	0.573	0.870	0.0000	2.79
	Cv	NA	NA	0.701	0.870	0.655	0.0191	1.84

nComp—number of selected components; NA—not available.

## Data Availability

Data is contained within the article or supplementary material.

## Supplementary Materials

The following are available online at https://www.mdpi.com/2304-8158/10/1/113/s1.Table S1: The loadings for the MSC-pretreated spectra samples on the two PC’s. Table S2. Instrumental texture analysis and TSS parameters. Table S3. Regression coefficients, standard error, p-values, and t-values for best iPLS on “hardness” parameter (ncomp = 4).

## References

[1] Kohyama K., Nishinari K. (2020). Chapter 1. Food Texture—Sensory Evaluation and Instrumental Measurement. Textural Characteristics of World Foods.

[2] Rolle L., Siret R., Río Segade S., Maury C., Gerbi V., Jourjon F. (2012). Instrumental texture analysis parameters as markers of table-grape and winegrape quality: A review. Am. J. Enol. Vitic..

[3] Yakushiji H., Sakurai N., Morinaga K. (2008). Changes in cell-wall polysaccharides from the mesocarp of grape berries during veraison. Physiol. Plant.

[4] Giacosa S., Zeppa G., Baiano A., Torchio F., Río Segade S., Gerbi V., Rolle L. (2015). Assessment of sensory firmness and crunchiness of table grapes by acoustic and mechanical properties. Aust. J. Grape Wine Res..

[5] Antonacci D., Genghi R., Perniola R., Alba V., Roccotelli S. (2015). Densità di impianto e qualità dell’uva Regal Seedless: Selezionato un clone più produttivo. Riv. Fruttic. Ortofloric. Ed. Agric..

[6] Bart J.C.J., Gucciardi E., Cavallaro S., Bart J.C.J., Gucciardi E., Cavallaro S. (2013). Chapter 8—Quality assurance of biolubricants. Woodhead Publishing Series in Energy, Biolubricants.

[7] Chung H. (2007). Applications of Near-Infrared Spectroscopy in Refineries and Important Issues to Address. Appl. Spectrosc. Rev..

[8] Beghi R., Buratti S., Giovenzana V., Benedetti S., Guidetti R. (2017). Electronic nose and visible-near infrared spectroscopy in fruit and vegetable monitoring. Rev. Anal. Chem..

[9] R Core Team (2016). R: A Language and Environment for Statistical Computing.

[10] Rolle L., Río Segade S., Torchio F., Giacosa S., Cagnasso E., Marengo F., Gerbi V. (2011). Influence of grape density and harvest date on changes in phenolic composition, phenol extractability indices, and instrumental texture properties during ripening. J. Agric. Food Chem..

[11] Rolle L., Giacosa S., Gerbi V., Novello V. (2011). Comparative study of texture properties, color characteristics, and chemical composition of ten white table-grape varieties. Am. J. Enol. Vitic..

[12] Kucheryavskiy S. (2020). mdatools—R package for chemometrics. Chemom. Intell. Lab. Syst..

[13] (2013). Signal Developers, Signal: Signal Processing. http://r-forge.r-project.org/projects/signal/.

[14] Vu V.Q. (2011). ggbiplot: A ggplot2 Based Biplot. R Package, Version 0.55. http://github.com/vqv/ggbiplot.

[15] Coombes K.R., Fritsche H.A., Clarke C., Chen J., Baggerly K.A., Morris J.S., Xiao L., Hung M., Kuerer H.M. (2003). Quality control and peak finding for proteomics data collected from nipple aspirate fluid by surface-enhanced laser desorption and ionization. Clin. Chem..

[16] Azzouz T., Puigdoménech A., Aragay M., Tauler R. (2003). Comparison between different data pretreatment methods in the analysis of forage samples using near-infrared diffuse reflectance spectroscopy and partial least-squares multivariate calibration method. Anal. Chim. Acta.

[17] Sabatier D., Dardenne P., Thuriès L. (2011). Near Infrared Reflectance Calibration Optimisation to Predict Lignocellulosic Compounds in Sugarcane Samples with Coarse Particle Size. J. Near Infrared Spectros..

[18] Awotwe-Otoo D., Zidanm A., Rahman Z., Habib M.J. (2012). Evaluation of Anticancer Drug-Loaded Nanoparticle Characteristics by Nondestructive Methodologies. AAPS PharmSciTech.

[19] Basile T., Marsico A.D., Cardone M.F., Antonacci D., Perniola R. (2020). FT-NIR Analysis of Intact Table Grape Berries to Understand Consumer Preference Driving Factors. Foods.

[20] Bampi M., Scheer A.D.P., de Castilhos F. (2013). Application of near infrared spectroscopy to predict the average droplet size and water content in biodiesel emulsions. Fuel.

[21] Rinnan Å., van den Berg F., Engelsen S.B. (2009). Review of the most common pre-processing techniques for near-infrared spectra. Trends Anal. Chem..

[22] Fernández-Novales J., Tardáguila J., Gutiérrez S., Diago M.P. (2019). On-The-Go VIS + SW − NIR Spectroscopy as a Reliable Monitoring Tool for Grape Composition within the Vineyard. Molecules.

[23] Sampaio P., Soares A., Castanho A., Almeida A.S., Oliveira J., Brites C. (2017). Dataset of Near-infrared spectroscopy measurement for amylose determination using PLS algorithms. Data Brief.

[24] Savitzky A., Golay M.J.E. (1964). Smoothing and differentiation of data by simplified least squares procedures. Anal. Chem..

[25] Xie S.F., Xiang B.R., Yu L.Y., Deng H.S. (2009). Tailoring noise frequency spectrum to improve NIR determinations. Talanta.

[26] Eriksson L., Johansson E., Kettaneh-Wold N., Trygg J., Wikström C., Wold S. (2006). Multi- and Megavariate Data Analysis. Part I: Basic Principles and Applications.

[27] Marsico A.D., Perniola R., Cardone M.F., Velenosi M., Antonacci D., Alba V., Basile T. (2018). Study of the Influence of Different Yeast Strains on Red Wine Fermentation with FT-NIR Spectroscopy and Principal Component Analysis. J. Multidiscip. Sci. J..

[28] Acri G., Testagrossa B., Vermiglio G. (2016). FT-NIR Analysis of Different Garlic Cultivars. J. Food Meas. Charact..

[29] Saha P., Roy N., Mukherjee D., Kumar Sarkar A. (2016). Application of principal component analysis for outlier detection in heterogeneous traffic data. Procedia Comput. Sci..

[30] Rodionova O.Y., Pomerantsev A.L. (2020). Detection of Outliers in Projection-Based Modeling. Anal. Chem..

[31] Pomerantsev A.L., Rodionova O.Y. (2014). Concept and role of extreme objects in PCA/SIMCA. J. Chemom..

[32] Pomerantsev A.L. (2008). Acceptance areas for multivariate classification derived by projection methods. J. Chemom..

[33] Rasti B., Scheunders P., Ghamisi P., Licciardi G., Chanussot J. (2018). Noise reduction in hyperspectral imagery: Overview and application. Remote Sens..

[34] Conzen J.P. (2014). Multivariate Calibration.

[35] Chang C.-W., Laird D.A., Mausbach M.J., Hurburgh C.R. (2001). Near-Infrared reflectance spectroscopy-principal components regression analyses of soil properties. Soil Sci. Soc. Am. J..

[36] Williams P.C., Sobering D.C. (1993). Comparison of commercial near infrared transmittance and reflectance instruments for analysis of whole grains and seeds. J. Near Infrared Spectrosc..

[37] Bellon-Maurel V., Fernandez-Ahumada E., Palagos B., Roger J.M., Mc Bratney A. (2010). Critical review of chemometric indicators commonly used for assessing the quality of the prediction of soil attributes by NIR spectroscopy. TrAC Trends Anal. Chem..

[38] Mehmood T., Hovde Liland K., Snipen L., Sæbø S. (2012). A review of variable selection methods in Partial Least Squares Regression. Chemom. Intell. Lab. Syst..

[39] Nørgaard L., Saudland A., Wagner J., Nielser J.P., Munck L., Engelsen S.B. (2000). Interval Partial Least-Squares Regression (iPLS): A Comparative Chemometric Study with an Example from Near-Infrared Spectroscopy. Appl. Spectrosc..

[40] ISO 21748:2017(EN) Guidance for The Use of Repeatability, Reproducibility and Trueness Estimates in Measurement Uncertainty Evaluation. https://www.iso.org/standard/71615.html.

[41] Cozzolino D., Cynkar W., Shah N., Smith P. (2011). Quantitative analysis of minerals and electric conductivity of red grape homogenates by near infrared reflectance spectroscopy. Comput. Electron. Agric..

[42] de Oliveira G.A., Bureau S., Renard C.M.G.C., Pereira-Netto A.B., de Castilhos F. (2014). Comparison of NIRS approach for prediction of internal quality traits in three fruit species. Food Chem..

