# Supplementary material for: NIR Analysis of Intact Grape Berries: Chemical and Physical Properties Prediction Using Multivariate Analysis
Source: Foods. 2021 Jan 7;10(1):113. doi: 10.3390/foods10010113 (PMC7827816; doi:10.3390/foods10010113)
Supplement: Supplementary file 1 [file foods-10-00113-s001.pdf]

Table S1. The loadings for the MSC pretreated spectra samples on the two PC's.

	PC1	PC2
[1,]	2.733938e-02	-1.660486e-02
[2,]	2.725230e-02	-1.760770e-02
[3,]	2.707246e-02	-1.797356e-02
[4,]	2.689235e-02	-1.817123e-02
[5,]	2.627180e-02	-1.895130e-02
[6,]	2.622129e-02	-1.970949e-02
[7,]	2.652210e-02	-1.927172e-02
[8,]	2.695249e-02	-1.799363e-02
[9,]	2.736243e-02	-1.687749e-02
[10,]	2.744361e-02	-1.679531e-02
[11,]	2.735153e-02	-1.738192e-02
[12,]	2.709423e-02	-1.755663e-02
[13,]	2.663474e-02	-1.720353e-02
[14,]	2.669455e-02	-1.700145e-02
[15,]	2.693453e-02	-1.763469e-02
[16,]	2.662303e-02	-1.919826e-02
[17,]	2.647403e-02	-2.002844e-02
[18,]	2.693032e-02	-1.933045e-02
[19,]	2.678360e-02	-1.861158e-02
[20,]	2.650316e-02	-1.854426e-02
[21,]	2.646659e-02	-1.818879e-02
[22,]	2.678954e-02	-1.809783e-02
[23,]	2.698792e-02	-1.796613e-02
[24,]	2.692982e-02	-1.783806e-02
[25,]	2.666944e-02	-1.816960e-02
[26,]	2.629518e-02	-1.886002e-02
[27,]	2.611801e-02	-1.977133e-02
[28,]	2.595904e-02	-2.020701e-02
[29,]	2.609096e-02	-2.009795e-02
[30,]	2.671486e-02	-1.917812e-02
[31,]	2.757213e-02	-1.736981e-02
[32,]	2.791214e-02	-1.616125e-02
[33,]	2.756471e-02	-1.655360e-02
[34,]	2.713104e-02	-1.763736e-02
[35,]	2.655236e-02	-1.855797e-02
[36,]	2.614701e-02	-1.926719e-02
[37,]	2.647648e-02	-1.907429e-02
[38,]	2.716412e-02	-1.787578e-02
[39,]	2.735811e-02	-1.667017e-02
[40,]	2.730435e-02	-1.601982e-02

[41,]	2.727794e-02 -1.583532e-02
[42,]	2.678397e-02 -1.669296e-02
[43,]	2.620833e-02 -1.839691e-02
[44,]	2.604669e-02 -1.931798e-02
[45,]	2.633001e-02 -1.905617e-02
[46,]	2.693670e-02 -1.886742e-02
[47,]	2.699745e-02 -1.933879e-02
[48,]	2.667608e-02 -1.982824e-02
[49,]	2.657040e-02 -1.907921e-02
[50,]	2.669225e-02 -1.745495e-02
[51,]	2.701090e-02 -1.700057e-02
[52,]	2.708492e-02 -1.825580e-02
[53,]	2.688381e-02 -1.913303e-02
[54,]	2.706933e-02 -1.873387e-02
[55,]	2.716769e-02 -1.838190e-02
[56,]	2.707841e-02 -1.848858e-02
[57,]	2.698773e-02 -1.819400e-02
[58,]	2.685973e-02 -1.806375e-02
[59,]	2.674017e-02 -1.873703e-02
[60,]	2.676562e-02 -1.922027e-02
[61,]	2.695473e-02 -1.886607e-02
[62,]	2.737900e-02 -1.777011e-02
[63,]	2.773882e-02 -1.585030e-02
[64,]	2.776415e-02 -1.540860e-02
[65,]	2.727307e-02 -1.709872e-02
[66,]	2.681016e-02 -1.850690e-02
[67,]	2.638544e-02 -1.888105e-02
[68,]	2.622439e-02 -1.890619e-02
[69,]	2.635467e-02 -1.845250e-02
[70,]	2.698066e-02 -1.825988e-02
[71,]	2.748068e-02 -1.848289e-02
[72,]	2.730695e-02 -1.827928e-02
[73,]	2.711133e-02 -1.718636e-02
[74,]	2.726904e-02 -1.592706e-02
[75,]	2.750638e-02 -1.590472e-02
[76,]	2.737100e-02 -1.694465e-02
[77,]	2.710652e-02 -1.762830e-02
[78,]	2.712197e-02 -1.729318e-02
[79,]	2.749136e-02 -1.644540e-02
[80,]	2.767000e-02 -1.621343e-02
[81,]	2.725489e-02 -1.772647e-02
[82,]	2.664805e-02 -1.957890e-02

[83,]	2.668072e-02 -1.910443e-02
[84,]	2.740033e-02 -1.711824e-02
[85,]	2.760473e-02 -1.630195e-02
[86,]	2.743705e-02 -1.646552e-02
[87,]	2.740189e-02 -1.640044e-02
[88,]	2.724602e-02 -1.674217e-02
[89,]	2.723574e-02 -1.775818e-02
[90,]	2.730652e-02 -1.836281e-02
[91,]	2.729034e-02 -1.836442e-02
[92,]	2.727936e-02 -1.787362e-02
[93,]	2.756299e-02 -1.695839e-02
[94,]	2.795899e-02 -1.627141e-02
[95,]	2.795619e-02 -1.660730e-02
[96,]	2.747305e-02 -1.791127e-02
[97,]	2.735773e-02 -1.840359e-02
[98,]	2.806856e-02 -1.663832e-02
[99,]	2.837391e-02 -1.426356e-02
[100,]	2.821189e-02 -1.401633e-02
[101,]	2.803031e-02 -1.450331e-02
[102,]	2.783654e-02 -1.483188e-02
[103,]	2.748628e-02 -1.580008e-02
[104,]	2.765398e-02 -1.613393e-02
[105,]	2.810322e-02 -1.566051e-02
[106,]	2.804673e-02 -1.553833e-02
[107,]	2.792840e-02 -1.543047e-02
[108,]	2.810913e-02 -1.547202e-02
[109,]	2.785646e-02 -1.600736e-02
[110,]	2.728369e-02 -1.618355e-02
[111,]	2.723179e-02 -1.495994e-02
[112,]	2.792791e-02 -1.329429e-02
[113,]	2.832436e-02 -1.281876e-02
[114,]	2.831693e-02 -1.407229e-02
[115,]	2.839146e-02 -1.531085e-02
[116,]	2.856329e-02 -1.487805e-02
[117,]	2.843246e-02 -1.482606e-02
[118,]	2.776861e-02 -1.665658e-02
[119,]	2.745293e-02 -1.792592e-02
[120,]	2.781954e-02 -1.731910e-02
[121,]	2.810397e-02 -1.652295e-02
[122,]	2.812623e-02 -1.647790e-02
[123,]	2.795904e-02 -1.604704e-02
[124,]	2.813677e-02 -1.515740e-02

[125,]	2.830530e-02 -1.489422e-02
[126,]	2.829137e-02 -1.516425e-02
[127,]	2.835870e-02 -1.518057e-02
[128,]	2.807168e-02 -1.525467e-02
[129,]	2.733327e-02 -1.615478e-02
[130,]	2.705693e-02 -1.729473e-02
[131,]	2.741146e-02 -1.726707e-02
[132,]	2.776389e-02 -1.649269e-02
[133,]	2.772612e-02 -1.644551e-02
[134,]	2.764480e-02 -1.726382e-02
[135,]	2.740725e-02 -1.779585e-02
[136,]	2.729687e-02 -1.734095e-02
[137,]	2.787336e-02 -1.621808e-02
[138,]	2.841863e-02 -1.472455e-02
[139,]	2.847428e-02 -1.348564e-02
[140,]	2.822369e-02 -1.339641e-02
[141,]	2.771252e-02 -1.458619e-02
[142,]	2.763991e-02 -1.610749e-02
[143,]	2.781362e-02 -1.678423e-02
[144,]	2.794732e-02 -1.614920e-02
[145,]	2.831092e-02 -1.468760e-02
[146,]	2.874216e-02 -1.370123e-02
[147,]	2.857147e-02 -1.383054e-02
[148,]	2.824749e-02 -1.407193e-02
[149,]	2.814869e-02 -1.364971e-02
[150,]	2.789668e-02 -1.352509e-02
[151,]	2.772139e-02 -1.451135e-02
[152,]	2.779634e-02 -1.547652e-02
[153,]	2.797365e-02 -1.600090e-02
[154,]	2.795872e-02 -1.663738e-02
[155,]	2.776477e-02 -1.676533e-02
[156,]	2.777657e-02 -1.642710e-02
[157,]	2.791799e-02 -1.618405e-02
[158,]	2.805809e-02 -1.589411e-02
[159,]	2.830345e-02 -1.518402e-02
[160,]	2.833637e-02 -1.474412e-02
[161,]	2.807224e-02 -1.539587e-02
[162,]	2.757480e-02 -1.558028e-02
[163,]	2.753365e-02 -1.544960e-02
[164,]	2.768194e-02 -1.591215e-02
[165,]	2.789059e-02 -1.610215e-02
[166,]	2.804438e-02 -1.565616e-02

[167,]	2.785028e-02 -1.513398e-02
[168,]	2.814358e-02 -1.509887e-02
[169,]	2.861480e-02 -1.496832e-02
[170,]	2.831643e-02 -1.491386e-02
[171,]	2.768610e-02 -1.529139e-02
[172,]	2.739511e-02 -1.533112e-02
[173,]	2.760440e-02 -1.497819e-02
[174,]	2.739881e-02 -1.446050e-02
[175,]	2.741441e-02 -1.431587e-02
[176,]	2.787290e-02 -1.423138e-02
[177,]	2.847596e-02 -1.376654e-02
[178,]	2.897278e-02 -1.319484e-02
[179,]	2.881722e-02 -1.355023e-02
[180,]	2.786183e-02 -1.484935e-02
[181,]	2.715549e-02 -1.568095e-02
[182,]	2.693490e-02 -1.499328e-02
[183,]	2.753483e-02 -1.392270e-02
[184,]	2.825264e-02 -1.311806e-02
[185,]	2.863761e-02 -1.292600e-02
[186,]	2.872742e-02 -1.352915e-02
[187,]	2.838853e-02 -1.384558e-02
[188,]	2.819815e-02 -1.354054e-02
[189,]	2.794506e-02 -1.322728e-02
[190,]	2.789053e-02 -1.307720e-02
[191,]	2.837639e-02 -1.283741e-02
[192,]	2.860025e-02 -1.212778e-02
[193,]	2.831454e-02 -1.096254e-02
[194,]	2.848218e-02 -1.068744e-02
[195,]	2.840995e-02 -1.209333e-02
[196,]	2.796623e-02 -1.432598e-02
[197,]	2.780909e-02 -1.547831e-02
[198,]	2.787054e-02 -1.419486e-02
[199,]	2.787631e-02 -1.119061e-02
[200,]	2.828504e-02 -8.940077e-03
[201,]	2.825364e-02 -8.898119e-03
[202,]	2.771061e-02 -1.053574e-02
[203,]	2.710407e-02 -1.202633e-02
[204,]	2.599119e-02 -1.269084e-02
[205,]	2.545113e-02 -1.409062e-02
[206,]	2.527750e-02 -1.584763e-02
[207,]	2.548271e-02 -1.618631e-02
[208,]	2.621985e-02 -1.473192e-02

[209,]	2.638251e-02	-1.240663e-02
[210,]	2.711530e-02	-1.163266e-02
[211,]	2.746742e-02	-1.151276e-02
[212,]	2.800214e-02	-1.091825e-02
[213,]	2.782662e-02	-1.133749e-02
[214,]	2.683795e-02	-1.263865e-02
[215,]	2.691184e-02	-1.145748e-02
[216,]	2.694875e-02	-9.054931e-03
[217,]	2.635344e-02	-7.066424e-03
[218,]	2.644139e-02	-4.854369e-03
[219,]	2.671931e-02	-3.801470e-03
[220,]	2.689687e-02	-3.538263e-03
[221,]	2.750842e-02	-2.188732e-03
[222,]	2.775895e-02	-2.637929e-03
[223,]	2.708437e-02	-6.554606e-03
[224,]	2.515296e-02	-9.409597e-03
[225,]	2.447699e-02	-7.926768e-03
[226,]	2.552791e-02	-5.384326e-03
[227,]	2.622063e-02	-5.943551e-03
[228,]	2.608563e-02	-6.284081e-03
[229,]	2.633913e-02	-3.802173e-03
[230,]	2.654356e-02	-2.287386e-03
[231,]	2.632683e-02	-3.332492e-03
[232,]	2.493308e-02	-4.316717e-03
[233,]	2.233680e-02	-2.952724e-03
[234,]	2.074932e-02	-1.667765e-03
[235,]	2.028863e-02	-1.156099e-03
[236,]	2.135890e-02	3.003770e-04
[237,]	2.285850e-02	1.544521e-03
[238,]	2.407546e-02	2.641418e-04
[239,]	2.272972e-02	-1.596548e-03
[240,]	1.903662e-02	-8.538290e-04
[241,]	1.741797e-02	3.935587e-04
[242,]	1.809141e-02	-1.003552e-03
[243,]	1.889443e-02	-2.857990e-03
[244,]	1.848607e-02	-6.771157e-04
[245,]	1.754958e-02	5.305838e-03
[246,]	1.859651e-02	9.142455e-03
[247,]	1.960835e-02	9.280097e-03
[248,]	1.683027e-02	8.709852e-03
[249,]	1.234607e-02	9.268386e-03
[250,]	8.435657e-03	9.118006e-03

[251,]	6.147521e-03	8.218450e-03
[252,]	6.127192e-03	1.107172e-02
[253,]	6.728450e-03	1.635329e-02
[254,]	6.935952e-03	1.883449e-02
[255,]	6.864269e-03	1.614537e-02
[256,]	6.363371e-03	1.227917e-02
[257,]	4.644230e-03	1.010478e-02
[258,]	3.314364e-03	9.226663e-03
[259,]	3.805460e-03	1.157266e-02
[260,]	1.781885e-03	1.509525e-02
[261,]	-3.442042e-03	1.460230e-02
[262,]	-7.611852e-03	1.117460e-02
[263,]	-1.063158e-02	1.097162e-02
[264,]	-1.151312e-02	1.623856e-02
[265,]	-1.151137e-02	2.242166e-02
[266,]	-1.230538e-02	2.420367e-02
[267,]	-1.233419e-02	2.172943e-02
[268,]	-1.190701e-02	1.986806e-02
[269,]	-1.225965e-02	1.946871e-02
[270,]	-1.360026e-02	2.052898e-02
[271,]	-1.414981e-02	2.149083e-02
[272,]	-1.309761e-02	2.002999e-02
[273,]	-1.255914e-02	1.888633e-02
[274,]	-1.294782e-02	2.105308e-02
[275,]	-1.397683e-02	2.411164e-02
[276,]	-1.526340e-02	2.549019e-02
[277,]	-1.635321e-02	2.595570e-02
[278,]	-1.723263e-02	2.419192e-02
[279,]	-1.828179e-02	2.145668e-02
[280,]	-1.908823e-02	1.987388e-02
[281,]	-1.946378e-02	2.055142e-02
[282,]	-1.933629e-02	2.307554e-02
[283,]	-1.901607e-02	2.288499e-02
[284,]	-2.000212e-02	2.102734e-02
[285,]	-2.066588e-02	2.041098e-02
[286,]	-2.047046e-02	2.086363e-02
[287,]	-1.959432e-02	1.915516e-02
[288,]	-1.820786e-02	1.685508e-02
[289,]	-1.757782e-02	1.738255e-02
[290,]	-1.763734e-02	1.925154e-02
[291,]	-1.828135e-02	2.158430e-02
[292,]	-1.821361e-02	2.347981e-02

[293,]	-1.693370e-02	2.221539e-02
[294,]	-1.756825e-02	1.983473e-02
[295,]	-2.006008e-02	1.932082e-02
[296,]	-2.175712e-02	1.949201e-02
[297,]	-2.204857e-02	1.777508e-02
[298,]	-2.190066e-02	1.655067e-02
[299,]	-2.166518e-02	1.783312e-02
[300,]	-2.155181e-02	1.916822e-02
[301,]	-2.244307e-02	1.890989e-02
[302,]	-2.353792e-02	1.768217e-02
[303,]	-2.407400e-02	1.804725e-02
[304,]	-2.434775e-02	2.087874e-02
[305,]	-2.326351e-02	2.297564e-02
[306,]	-2.208192e-02	2.342918e-02
[307,]	-2.222310e-02	2.249096e-02
[308,]	-2.266153e-02	2.091797e-02
[309,]	-2.274497e-02	2.167998e-02
[310,]	-2.268312e-02	2.290481e-02
[311,]	-2.281554e-02	2.157867e-02
[312,]	-2.345890e-02	2.063195e-02
[313,]	-2.383435e-02	2.219306e-02
[314,]	-2.311301e-02	2.356677e-02
[315,]	-2.267240e-02	2.275397e-02
[316,]	-2.306499e-02	2.142039e-02
[317,]	-2.302245e-02	2.151054e-02
[318,]	-2.208534e-02	2.259505e-02
[319,]	-2.183100e-02	2.389177e-02
[320,]	-2.229028e-02	2.442685e-02
[321,]	-2.224793e-02	2.407772e-02
[322,]	-2.269084e-02	2.358129e-02
[323,]	-2.364555e-02	2.201371e-02
[324,]	-2.394976e-02	2.044531e-02
[325,]	-2.418328e-02	2.059488e-02
[326,]	-2.458011e-02	2.096329e-02
[327,]	-2.462836e-02	2.050163e-02
[328,]	-2.434276e-02	2.007690e-02
[329,]	-2.375963e-02	2.075168e-02
[330,]	-2.348259e-02	2.186605e-02
[331,]	-2.370866e-02	2.139607e-02
[332,]	-2.384801e-02	2.107809e-02
[333,]	-2.353296e-02	2.200209e-02
[334,]	-2.335523e-02	2.264453e-02

[335,]	-2.345622e-02	2.236540e-02
[336,]	-2.387188e-02	2.213196e-02
[337,]	-2.424905e-02	2.237778e-02
[338,]	-2.357623e-02	2.218294e-02
[339,]	-2.316808e-02	2.241786e-02
[340,]	-2.334892e-02	2.316088e-02
[341,]	-2.379025e-02	2.364767e-02
[342,]	-2.442165e-02	2.325434e-02
[343,]	-2.430526e-02	2.281203e-02
[344,]	-2.322025e-02	2.374700e-02
[345,]	-2.259034e-02	2.428590e-02
[346,]	-2.380830e-02	2.328475e-02
[347,]	-2.475839e-02	2.168556e-02
[348,]	-2.473356e-02	2.167727e-02
[349,]	-2.388373e-02	2.254074e-02
[350,]	-2.260472e-02	2.313545e-02
[351,]	-2.224514e-02	2.399536e-02
[352,]	-2.248568e-02	2.488915e-02
[353,]	-2.243104e-02	2.518256e-02
[354,]	-2.297699e-02	2.442642e-02
[355,]	-2.424819e-02	2.156586e-02
[356,]	-2.543061e-02	1.972261e-02
[357,]	-2.511407e-02	2.040806e-02
[358,]	-2.427428e-02	2.238570e-02
[359,]	-2.330701e-02	2.419392e-02
[360,]	-2.280952e-02	2.543611e-02
[361,]	-2.260246e-02	2.468082e-02
[362,]	-2.315987e-02	2.362526e-02
[363,]	-2.354856e-02	2.359181e-02
[364,]	-2.227256e-02	2.469099e-02
[365,]	-2.097367e-02	2.614567e-02
[366,]	-2.101455e-02	2.628327e-02
[367,]	-2.171818e-02	2.530319e-02
[368,]	-2.236640e-02	2.374616e-02
[369,]	-2.321750e-02	2.256398e-02
[370,]	-2.283847e-02	2.265171e-02
[371,]	-2.221251e-02	2.410343e-02
[372,]	-2.244812e-02	2.503128e-02
[373,]	-2.209087e-02	2.522330e-02
[374,]	-2.092482e-02	2.646545e-02
[375,]	-2.049144e-02	2.746545e-02
[376,]	-2.181471e-02	2.649454e-02

[377,]	-2.333547e-02	2.369243e-02
[378,]	-2.435447e-02	2.146369e-02
[379,]	-2.475847e-02	2.113416e-02
[380,]	-2.440839e-02	2.200628e-02
[381,]	-2.353624e-02	2.269170e-02
[382,]	-2.267281e-02	2.383906e-02
[383,]	-2.174429e-02	2.495648e-02
[384,]	-2.159027e-02	2.484598e-02
[385,]	-2.243267e-02	2.471505e-02
[386,]	-2.288126e-02	2.543883e-02
[387,]	-2.266613e-02	2.600051e-02
[388,]	-2.237085e-02	2.611858e-02
[389,]	-2.169223e-02	2.514763e-02
[390,]	-2.121308e-02	2.399620e-02
[391,]	-2.099247e-02	2.447757e-02
[392,]	-2.053083e-02	2.609972e-02
[393,]	-2.040988e-02	2.633958e-02
[394,]	-2.088884e-02	2.514915e-02
[395,]	-2.079213e-02	2.451232e-02
[396,]	-1.970445e-02	2.512450e-02
[397,]	-1.911771e-02	2.686719e-02
[398,]	-1.927779e-02	2.820755e-02
[399,]	-1.963768e-02	2.827092e-02
[400,]	-1.966808e-02	2.775501e-02
[401,]	-1.939264e-02	2.757598e-02
[402,]	-1.881828e-02	2.805305e-02
[403,]	-1.789895e-02	2.878775e-02
[404,]	-1.743769e-02	2.939548e-02
[405,]	-1.728785e-02	2.874010e-02
[406,]	-1.699434e-02	2.720562e-02
[407,]	-1.664484e-02	2.712559e-02
[408,]	-1.697884e-02	2.824967e-02
[409,]	-1.797936e-02	2.875617e-02
[410,]	-1.804867e-02	2.776690e-02
[411,]	-1.705824e-02	2.638700e-02
[412,]	-1.677848e-02	2.583414e-02
[413,]	-1.788297e-02	2.484855e-02
[414,]	-1.977303e-02	2.277150e-02
[415,]	-2.030932e-02	2.162881e-02
[416,]	-1.923418e-02	2.363747e-02
[417,]	-1.767397e-02	2.661718e-02
[418,]	-1.685315e-02	2.821714e-02

[419,]	-1.608300e-02	2.865417e-02
[420,]	-1.542328e-02	2.900379e-02
[421,]	-1.469053e-02	2.943388e-02
[422,]	-1.380316e-02	3.012125e-02
[423,]	-1.329271e-02	3.053458e-02
[424,]	-1.283483e-02	3.074721e-02
[425,]	-1.183905e-02	3.165006e-02
[426,]	-1.121515e-02	3.319268e-02
[427,]	-1.180477e-02	3.309335e-02
[428,]	-1.298956e-02	3.224108e-02
[429,]	-1.255644e-02	3.188139e-02
[430,]	-1.017446e-02	3.196062e-02
[431,]	-8.034967e-03	3.187969e-02
[432,]	-8.281349e-03	3.084485e-02
[433,]	-9.327743e-03	2.898715e-02
[434,]	-9.151227e-03	2.895780e-02
[435,]	-8.123164e-03	2.985476e-02
[436,]	-7.805968e-03	3.036683e-02
[437,]	-7.555617e-03	2.991917e-02
[438,]	-5.835261e-03	3.033115e-02
[439,]	-3.524288e-03	3.088315e-02
[440,]	-3.383133e-03	3.085197e-02
[441,]	-4.969086e-03	2.951046e-02
[442,]	-6.272384e-03	2.825161e-02
[443,]	-6.833689e-03	2.804828e-02
[444,]	-5.872507e-03	2.851118e-02
[445,]	-2.857421e-03	3.075036e-02
[446,]	3.325763e-04	3.206920e-02
[447,]	1.409107e-03	3.209275e-02
[448,]	7.644124e-04	3.186508e-02
[449,]	-1.045138e-03	3.152224e-02
[450,]	-2.221035e-03	3.051689e-02
[451,]	-1.005175e-03	2.831243e-02
[452,]	8.307989e-04	2.656287e-02
[453,]	1.279737e-03	2.742324e-02
[454,]	1.365889e-03	2.993999e-02
[455,]	2.102494e-03	2.921047e-02
[456,]	3.247699e-03	2.618465e-02
[457,]	5.026842e-03	2.406791e-02
[458,]	7.697815e-03	2.405181e-02
[459,]	9.924691e-03	2.479275e-02
[460,]	1.069543e-02	2.459144e-02

[461,]	9.932756e-03	2.339698e-02
[462,]	8.664986e-03	2.502837e-02
[463,]	9.145343e-03	2.887028e-02
[464,]	1.233077e-02	3.051427e-02
[465,]	1.435073e-02	2.784969e-02
[466,]	1.262601e-02	2.350737e-02
[467,]	1.024587e-02	2.147406e-02
[468,]	9.801323e-03	2.236121e-02
[469,]	1.087394e-02	2.448728e-02
[470,]	1.182054e-02	2.580515e-02
[471,]	1.220649e-02	2.431007e-02
[472,]	1.308160e-02	2.097324e-02
[473,]	1.467954e-02	1.832460e-02
[474,]	1.695553e-02	1.888312e-02
[475,]	1.930137e-02	1.946344e-02
[476,]	2.119698e-02	1.703721e-02
[477,]	2.300875e-02	1.452947e-02
[478,]	2.297502e-02	1.373550e-02
[479,]	2.223511e-02	1.482874e-02
[480,]	2.221808e-02	1.692612e-02
[481,]	2.228576e-02	1.753828e-02
[482,]	2.206528e-02	1.610241e-02
[483,]	2.101831e-02	1.546029e-02
[484,]	1.939741e-02	1.616305e-02
[485,]	1.933768e-02	1.603224e-02
[486,]	2.142742e-02	1.443467e-02
[487,]	2.364627e-02	1.291827e-02
[488,]	2.376406e-02	1.216497e-02
[489,]	2.395174e-02	1.271645e-02
[490,]	2.463496e-02	1.380215e-02
[491,]	2.526834e-02	1.405073e-02
[492,]	2.622040e-02	1.303077e-02
[493,]	2.700457e-02	1.091233e-02
[494,]	2.692215e-02	8.948817e-03
[495,]	2.661293e-02	8.778212e-03
[496,]	2.660169e-02	1.056903e-02
[497,]	2.579966e-02	1.228808e-02
[498,]	2.520860e-02	1.293151e-02
[499,]	2.585133e-02	1.358015e-02
[500,]	2.596750e-02	1.399493e-02
[501,]	2.599810e-02	1.364526e-02
[502,]	2.656117e-02	1.213570e-02

[503,]	2.661694e-02	9.963286e-03
[504,]	2.692659e-02	8.794756e-03
[505,]	2.730900e-02	7.705908e-03
[506,]	2.729113e-02	6.010754e-03
[507,]	2.651370e-02	5.559843e-03
[508,]	2.600550e-02	6.670125e-03
[509,]	2.667222e-02	6.883707e-03
[510,]	2.786191e-02	5.416398e-03
[511,]	2.855306e-02	4.406537e-03
[512,]	2.838238e-02	4.941220e-03
[513,]	2.842533e-02	6.458279e-03
[514,]	2.825144e-02	7.849487e-03
[515,]	2.816994e-02	7.785973e-03
[516,]	2.813247e-02	5.610292e-03
[517,]	2.788137e-02	3.540757e-03
[518,]	2.819874e-02	3.824055e-03
[519,]	2.816933e-02	4.484740e-03
[520,]	2.786627e-02	3.631269e-03
[521,]	2.816347e-02	2.988384e-03
[522,]	2.834532e-02	2.883387e-03
[523,]	2.858400e-02	2.481129e-03
[524,]	2.878198e-02	2.543912e-03
[525,]	2.892927e-02	3.232346e-03
[526,]	2.903670e-02	3.071083e-03
[527,]	2.929071e-02	2.124016e-03
[528,]	2.918580e-02	2.115862e-03
[529,]	2.915491e-02	3.401220e-03
[530,]	2.909878e-02	4.137335e-03
[531,]	2.887826e-02	3.295092e-03
[532,]	2.865268e-02	2.088938e-03
[533,]	2.873930e-02	1.850857e-03
[534,]	2.895700e-02	2.643373e-03
[535,]	2.929982e-02	3.056847e-03
[536,]	2.934405e-02	2.699639e-03
[537,]	2.934862e-02	2.920980e-03
[538,]	2.937395e-02	3.291012e-03
[539,]	2.948772e-02	2.731107e-03
[540,]	2.960912e-02	2.124198e-03
[541,]	2.964891e-02	2.396511e-03
[542,]	2.980692e-02	2.503236e-03
[543,]	2.977824e-02	1.982113e-03
[544,]	2.976864e-02	2.338603e-03

[545,]	2.963617e-02	3.172780e-03
[546,]	2.933593e-02	2.665699e-03
[547,]	2.953120e-02	1.769419e-03
[548,]	2.962346e-02	1.835827e-03
[549,]	2.959004e-02	2.200375e-03
[550,]	2.961811e-02	2.249449e-03
[551,]	2.972533e-02	2.436157e-03
[552,]	2.992670e-02	2.821727e-03
[553,]	3.001055e-02	2.680228e-03
[554,]	3.009101e-02	2.073463e-03
[555,]	3.010618e-02	2.420955e-03
[556,]	2.981977e-02	3.959708e-03
[557,]	2.948053e-02	4.782915e-03
[558,]	2.927928e-02	3.851183e-03
[559,]	2.895724e-02	2.763288e-03
[560,]	2.893746e-02	2.968106e-03
[561,]	2.925506e-02	3.276203e-03
[562,]	2.956979e-02	2.691647e-03
[563,]	2.984862e-02	1.458441e-03
[564,]	2.995440e-02	5.162015e-05
[565,]	2.997202e-02	-4.579973e-04
[566,]	2.961933e-02	-4.430556e-05
[567,]	2.930690e-02	1.050964e-03
[568,]	2.908657e-02	2.703465e-03
[569,]	2.894540e-02	3.228557e-03
[570,]	2.910409e-02	1.621239e-03
[571,]	2.931714e-02	2.016450e-04
[572,]	2.955043e-02	8.283266e-04
[573,]	2.954237e-02	2.092640e-03
[574,]	2.952133e-02	2.699680e-03
[575,]	2.942183e-02	2.835984e-03
[576,]	2.925111e-02	2.990927e-03
[577,]	2.914759e-02	3.532760e-03
[578,]	2.918492e-02	3.996601e-03
[579,]	2.946096e-02	3.974272e-03
[580,]	2.968314e-02	3.110750e-03
[581,]	2.941171e-02	1.565773e-03
[582,]	2.895397e-02	1.037318e-03
[583,]	2.872262e-02	1.869375e-03
[584,]	2.911711e-02	2.713094e-03
[585,]	2.966561e-02	3.612144e-03
[586,]	2.969460e-02	4.893473e-03

[587,]	2.944595e-02	6.243117e-03
[588,]	2.910091e-02	7.695863e-03
[589,]	2.890392e-02	8.389414e-03
[590,]	2.927537e-02	7.498813e-03
[591,]	2.967787e-02	6.128695e-03
[592,]	2.947266e-02	5.269988e-03
[593,]	2.920850e-02	4.847544e-03
[594,]	2.896876e-02	5.018580e-03
[595,]	2.885172e-02	6.012157e-03
[596,]	2.870106e-02	7.159729e-03
[597,]	2.844164e-02	7.374170e-03
[598,]	2.824698e-02	6.317895e-03
[599,]	2.830936e-02	5.028891e-03
[600,]	2.805040e-02	4.650337e-03
[601,]	2.738372e-02	5.559436e-03
[602,]	2.683962e-02	6.825333e-03
[603,]	2.693037e-02	7.104912e-03
[604,]	2.767702e-02	7.010327e-03
[605,]	2.831687e-02	7.851977e-03
[606,]	2.848514e-02	8.981869e-03
[607,]	2.854042e-02	9.264612e-03
[608,]	2.804371e-02	8.881118e-03
[609,]	2.727194e-02	8.336686e-03
[610,]	2.721320e-02	8.285115e-03
[611,]	2.713346e-02	9.894002e-03
[612,]	2.676499e-02	1.286649e-02
[613,]	2.624900e-02	1.461612e-02
[614,]	2.599814e-02	1.473152e-02
[615,]	2.542591e-02	1.511080e-02
[616,]	2.524641e-02	1.549529e-02
[617,]	2.597691e-02	1.457759e-02
[618,]	2.667071e-02	1.445323e-02
[619,]	2.596579e-02	1.577988e-02
[620,]	2.513475e-02	1.645668e-02
[621,]	2.556152e-02	1.603891e-02
[622,]	2.605041e-02	1.471426e-02
[623,]	2.545185e-02	1.335852e-02
[624,]	2.447521e-02	1.329777e-02
[625,]	2.404347e-02	1.441758e-02
[626,]	2.393240e-02	1.595650e-02
[627,]	2.355405e-02	1.720470e-02
[628,]	2.317585e-02	1.742592e-02

[629,]	2.395934e-02	1.761892e-02
[630,]	2.451044e-02	1.794061e-02
[631,]	2.420200e-02	1.782639e-02
[632,]	2.353717e-02	1.794041e-02
[633,]	2.342069e-02	1.929175e-02
[634,]	2.344023e-02	2.028007e-02
[635,]	2.283848e-02	2.070242e-02
[636,]	2.206811e-02	2.114116e-02
[637,]	2.162009e-02	2.032479e-02
[638,]	2.137478e-02	1.881477e-02
[639,]	2.106720e-02	1.899560e-02
[640,]	1.985867e-02	2.085177e-02
[641,]	1.875878e-02	2.324467e-02
[642,]	1.838238e-02	2.463007e-02
[643,]	1.792266e-02	2.537650e-02
[644,]	1.674160e-02	2.562190e-02
[645,]	1.623119e-02	2.501017e-02
[646,]	1.724145e-02	2.410698e-02
[647,]	1.784172e-02	2.308315e-02
[648,]	1.733487e-02	2.265519e-02
[649,]	1.704367e-02	2.366280e-02
[650,]	1.691136e-02	2.543268e-02
[651,]	1.611397e-02	2.661065e-02
[652,]	1.488329e-02	2.723027e-02
[653,]	1.357720e-02	2.819483e-02
[654,]	1.206821e-02	2.879572e-02
[655,]	1.084737e-02	2.872601e-02
[656,]	1.046551e-02	2.888925e-02
[657,]	1.076315e-02	2.936305e-02
[658,]	1.057453e-02	3.034140e-02
[659,]	9.297077e-03	3.223588e-02
[660,]	7.956156e-03	3.369079e-02
[661,]	7.216876e-03	3.273121e-02
[662,]	6.449736e-03	3.111118e-02
[663,]	4.648893e-03	3.109339e-02
[664,]	2.591964e-03	3.281596e-02
[665,]	1.296322e-03	3.382419e-02
[666,]	2.268695e-04	3.372392e-02
[667,]	-1.382867e-03	3.357492e-02
[668,]	-3.411153e-03	3.357238e-02
[669,]	-5.122122e-03	3.345386e-02
[670,]	-5.072234e-03	3.265369e-02

[671,]	-3.664165e-03	3.310144e-02
[672,]	-3.817554e-03	3.449755e-02
[673,]	-6.050952e-03	3.527751e-02
[674,]	-8.757995e-03	3.488495e-02
[675,]	-1.120822e-02	3.351353e-02
[676,]	-1.215664e-02	3.250485e-02
[677,]	-1.195218e-02	3.254748e-02
[678,]	-1.270092e-02	3.260602e-02
[679,]	-1.478305e-02	3.216844e-02
[680,]	-1.640721e-02	3.198401e-02
[681,]	-1.692517e-02	3.233751e-02
[682,]	-1.750594e-02	3.196956e-02
[683,]	-1.908482e-02	3.070930e-02
[684,]	-2.073320e-02	2.895210e-02
[685,]	-2.154525e-02	2.778911e-02
[686,]	-2.150550e-02	2.810348e-02
[687,]	-2.168012e-02	2.850373e-02
[688,]	-2.262222e-02	2.758040e-02
[689,]	-2.333034e-02	2.666109e-02
[690,]	-2.322457e-02	2.656555e-02
[691,]	-2.317676e-02	2.667384e-02
[692,]	-2.363682e-02	2.626942e-02
[693,]	-2.437806e-02	2.517479e-02
[694,]	-2.501302e-02	2.387927e-02
[695,]	-2.532781e-02	2.317586e-02
[696,]	-2.548915e-02	2.331170e-02
[697,]	-2.565141e-02	2.336859e-02
[698,]	-2.614991e-02	2.248341e-02
[699,]	-2.683221e-02	2.135982e-02
[700,]	-2.713757e-02	2.092122e-02
[701,]	-2.718289e-02	2.096871e-02
[702,]	-2.724197e-02	2.107426e-02
[703,]	-2.727453e-02	2.094939e-02
[704,]	-2.758705e-02	2.010430e-02
[705,]	-2.817945e-02	1.866140e-02
[706,]	-2.863236e-02	1.756102e-02
[707,]	-2.875146e-02	1.723248e-02
[708,]	-2.864491e-02	1.748557e-02
[709,]	-2.851796e-02	1.794128e-02
[710,]	-2.853472e-02	1.792565e-02
[711,]	-2.864461e-02	1.737178e-02
[712,]	-2.878542e-02	1.700016e-02

[713,]	-2.895705e-02	1.671944e-02
[714,]	-2.905456e-02	1.621448e-02
[715,]	-2.920711e-02	1.593094e-02
[716,]	-2.938843e-02	1.568379e-02
[717,]	-2.957234e-02	1.496868e-02
[718,]	-2.967827e-02	1.444645e-02
[719,]	-2.957858e-02	1.465574e-02
[720,]	-2.952004e-02	1.468132e-02
[721,]	-2.965021e-02	1.415588e-02
[722,]	-2.980326e-02	1.375197e-02
[723,]	-2.987308e-02	1.363353e-02
[724,]	-2.985203e-02	1.379106e-02
[725,]	-2.980759e-02	1.406013e-02
[726,]	-2.990821e-02	1.366911e-02
[727,]	-3.010261e-02	1.274716e-02
[728,]	-3.019304e-02	1.223849e-02
[729,]	-3.024795e-02	1.234948e-02
[730,]	-3.025477e-02	1.272494e-02
[731,]	-3.020759e-02	1.285295e-02
[732,]	-3.015747e-02	1.256325e-02
[733,]	-3.013113e-02	1.228689e-02
[734,]	-3.025211e-02	1.236237e-02
[735,]	-3.030632e-02	1.251886e-02
[736,]	-3.019261e-02	1.256229e-02
[737,]	-3.013944e-02	1.260801e-02
[738,]	-3.020184e-02	1.263392e-02
[739,]	-3.031256e-02	1.240136e-02
[740,]	-3.043091e-02	1.187420e-02
[741,]	-3.046168e-02	1.145255e-02
[742,]	-3.040669e-02	1.135369e-02
[743,]	-3.040534e-02	1.135238e-02
[744,]	-3.049491e-02	1.121493e-02
[745,]	-3.053533e-02	1.108225e-02
[746,]	-3.043146e-02	1.139784e-02
[747,]	-3.023340e-02	1.208054e-02
[748,]	-3.012951e-02	1.240560e-02
[749,]	-3.028543e-02	1.200158e-02
[750,]	-3.049299e-02	1.119416e-02
[751,]	-3.057492e-02	1.058000e-02
[752,]	-3.058781e-02	1.053415e-02
[753,]	-3.056672e-02	1.106664e-02
[754,]	-3.042845e-02	1.189095e-02

[755,]	-3.031060e-02	1.239438e-02
[756,]	-3.037950e-02	1.198181e-02
[757,]	-3.054804e-02	1.102111e-02
[758,]	-3.064241e-02	1.037597e-02
[759,]	-3.065697e-02	1.040567e-02
[760,]	-3.051968e-02	1.104083e-02
[761,]	-3.033439e-02	1.181003e-02
[762,]	-3.028404e-02	1.208494e-02
[763,]	-3.034310e-02	1.194760e-02
[764,]	-3.046853e-02	1.167455e-02
[765,]	-3.057567e-02	1.112116e-02
[766,]	-3.066197e-02	1.060296e-02
[767,]	-3.068658e-02	1.049676e-02
[768,]	-3.063484e-02	1.052250e-02
[769,]	-3.059907e-02	1.071200e-02
[770,]	-3.051459e-02	1.124405e-02
[771,]	-3.042653e-02	1.176677e-02
[772,]	-3.036879e-02	1.187587e-02
[773,]	-3.047219e-02	1.164003e-02
[774,]	-3.054499e-02	1.118051e-02
[775,]	-3.061446e-02	1.060576e-02
[776,]	-3.075762e-02	1.005047e-02
[777,]	-3.083337e-02	9.721260e-03
[778,]	-3.076327e-02	9.882471e-03
[779,]	-3.071750e-02	1.011537e-02
[780,]	-3.074038e-02	9.900638e-03
[781,]	-3.073449e-02	9.769855e-03
[782,]	-3.063896e-02	1.014226e-02
[783,]	-3.061546e-02	1.044283e-02
[784,]	-3.069079e-02	1.019419e-02
[785,]	-3.072684e-02	1.008851e-02
[786,]	-3.068090e-02	1.062442e-02
[787,]	-3.064165e-02	1.076639e-02
[788,]	-3.071972e-02	1.001761e-02
[789,]	-3.079447e-02	9.538877e-03
[790,]	-3.076977e-02	9.905031e-03
[791,]	-3.068928e-02	1.033576e-02
[792,]	-3.065591e-02	1.038520e-02
[793,]	-3.068085e-02	1.018457e-02
[794,]	-3.074693e-02	9.863202e-03
[795,]	-3.074603e-02	9.773308e-03
[796,]	-3.069887e-02	1.014121e-02

[797,]	-3.069198e-02	1.036060e-02
[798,]	-3.070582e-02	9.951005e-03
[799,]	-3.074836e-02	9.547645e-03
[800,]	-3.075284e-02	9.706318e-03
[801,]	-3.069344e-02	9.986307e-03
[802,]	-3.068916e-02	9.857954e-03
[803,]	-3.072799e-02	9.667326e-03
[804,]	-3.067625e-02	9.892403e-03
[805,]	-3.061872e-02	1.003850e-02
[806,]	-3.073286e-02	9.624183e-03
[807,]	-3.086231e-02	9.223416e-03
[808,]	-3.084338e-02	9.412586e-03
[809,]	-3.076356e-02	9.765861e-03
[810,]	-3.075506e-02	9.657927e-03
[811,]	-3.084181e-02	9.131425e-03
[812,]	-3.091506e-02	8.876354e-03
[813,]	-3.089437e-02	9.328137e-03
[814,]	-3.076410e-02	9.944193e-03
[815,]	-3.067863e-02	1.020791e-02
[816,]	-3.063713e-02	1.024088e-02
[817,]	-3.062440e-02	1.028783e-02
[818,]	-3.064921e-02	1.041815e-02
[819,]	-3.066649e-02	1.038211e-02
[820,]	-3.067966e-02	1.020249e-02
[821,]	-3.068755e-02	1.039712e-02
[822,]	-3.065301e-02	1.075705e-02
[823,]	-3.065507e-02	1.066297e-02
[824,]	-3.070027e-02	1.026518e-02
[825,]	-3.067633e-02	1.027142e-02
[826,]	-3.055169e-02	1.085235e-02
[827,]	-3.048563e-02	1.120578e-02
[828,]	-3.058762e-02	1.088588e-02
[829,]	-3.065790e-02	1.055913e-02
[830,]	-3.060342e-02	1.061595e-02
[831,]	-3.057432e-02	1.061675e-02
[832,]	-3.055879e-02	1.067444e-02
[833,]	-3.056396e-02	1.079691e-02
[834,]	-3.065324e-02	1.055338e-02
[835,]	-3.067160e-02	1.049590e-02
[836,]	-3.057488e-02	1.095807e-02
[837,]	-3.054620e-02	1.119664e-02
[838,]	-3.063099e-02	1.086075e-02

[839,]	-3.071987e-02	1.038729e-02
[840,]	-3.072593e-02	1.033490e-02
[841,]	-3.069001e-02	1.063008e-02
[842,]	-3.060201e-02	1.079633e-02
[843,]	-3.053487e-02	1.097448e-02
[844,]	-3.048925e-02	1.129510e-02
[845,]	-3.043434e-02	1.176181e-02
[846,]	-3.038426e-02	1.221660e-02
[847,]	-3.037606e-02	1.229880e-02
[848,]	-3.034498e-02	1.224587e-02
[849,]	-3.026580e-02	1.227392e-02
[850,]	-3.024561e-02	1.224532e-02
[851,]	-3.033994e-02	1.212443e-02
[852,]	-3.043195e-02	1.173281e-02
[853,]	-3.052704e-02	1.113291e-02
[854,]	-3.059303e-02	1.081184e-02
[855,]	-3.055767e-02	1.093219e-02
[856,]	-3.050622e-02	1.119234e-02
[857,]	-3.048010e-02	1.142369e-02
[858,]	-3.046607e-02	1.167499e-02
[859,]	-3.043770e-02	1.183457e-02
[860,]	-3.042671e-02	1.188050e-02
[861,]	-3.040331e-02	1.194944e-02
[862,]	-3.039568e-02	1.191710e-02
[863,]	-3.043413e-02	1.159256e-02
[864,]	-3.047875e-02	1.136578e-02
[865,]	-3.049347e-02	1.155867e-02
[866,]	-3.046762e-02	1.188778e-02
[867,]	-3.043436e-02	1.211029e-02
[868,]	-3.036361e-02	1.229928e-02
[869,]	-3.026923e-02	1.261735e-02
[870,]	-3.021381e-02	1.287693e-02
[871,]	-3.015666e-02	1.276734e-02
[872,]	-3.016354e-02	1.247007e-02
[873,]	-3.021553e-02	1.226548e-02
[874,]	-3.028561e-02	1.228740e-02
[875,]	-3.037711e-02	1.223760e-02
[876,]	-3.041259e-02	1.213450e-02
[877,]	-3.034758e-02	1.237423e-02
[878,]	-3.034110e-02	1.233072e-02
[879,]	-3.042145e-02	1.180310e-02
[880,]	-3.041066e-02	1.170164e-02

[881,]	-3.030217e-02	1.220089e-02
[882,]	-3.020924e-02	1.288559e-02
[883,]	-3.010971e-02	1.353137e-02
[884,]	-2.998333e-02	1.384974e-02
[885,]	-2.998182e-02	1.364258e-02
[886,]	-3.011404e-02	1.316790e-02
[887,]	-3.015376e-02	1.303692e-02
[888,]	-2.998938e-02	1.349891e-02
[889,]	-2.979682e-02	1.410488e-02
[890,]	-2.978560e-02	1.429983e-02
[891,]	-2.993688e-02	1.382635e-02
[892,]	-3.013419e-02	1.299265e-02
[893,]	-3.027840e-02	1.243364e-02
[894,]	-3.030291e-02	1.237304e-02
[895,]	-3.025986e-02	1.252733e-02
[896,]	-3.025179e-02	1.259120e-02
[897,]	-3.024174e-02	1.262454e-02
[898,]	-3.021163e-02	1.273385e-02
[899,]	-3.022036e-02	1.281766e-02
[900,]	-3.023371e-02	1.290202e-02
[901,]	-3.015587e-02	1.300554e-02
[902,]	-3.005710e-02	1.307012e-02
[903,]	-3.003688e-02	1.311816e-02
[904,]	-3.006020e-02	1.317203e-02
[905,]	-3.004691e-02	1.323697e-02
[906,]	-3.000547e-02	1.340645e-02
[907,]	-2.998557e-02	1.360200e-02
[908,]	-3.005123e-02	1.357787e-02
[909,]	-3.009474e-02	1.342410e-02
[910,]	-2.999582e-02	1.353515e-02
[911,]	-2.984575e-02	1.388645e-02
[912,]	-2.982065e-02	1.400588e-02
[913,]	-2.995878e-02	1.376248e-02
[914,]	-3.001507e-02	1.354487e-02
[915,]	-2.998647e-02	1.359724e-02
[916,]	-2.994856e-02	1.382791e-02
[917,]	-2.991706e-02	1.416639e-02
[918,]	-2.994978e-02	1.417690e-02
[919,]	-3.008048e-02	1.361235e-02
[920,]	-3.022822e-02	1.306855e-02
[921,]	-3.026281e-02	1.299428e-02
[922,]	-3.018480e-02	1.314681e-02

[923,]	-3.014649e-02	1.321138e-02
[924,]	-3.014909e-02	1.312823e-02
[925,]	-3.015238e-02	1.306863e-02
[926,]	-3.011902e-02	1.315012e-02
[927,]	-3.010664e-02	1.303394e-02
[928,]	-3.016498e-02	1.244068e-02
[929,]	-3.026969e-02	1.184787e-02
[930,]	-3.033569e-02	1.171054e-02
[931,]	-3.029150e-02	1.189312e-02
[932,]	-3.022844e-02	1.244355e-02
[933,]	-3.009559e-02	1.318409e-02
[934,]	-3.004106e-02	1.348180e-02
[935,]	-3.011933e-02	1.338666e-02
[936,]	-3.016455e-02	1.333776e-02
[937,]	-3.014726e-02	1.328975e-02
[938,]	-3.016696e-02	1.314073e-02
[939,]	-3.021503e-02	1.288671e-02
[940,]	-3.022637e-02	1.274073e-02
[941,]	-3.024147e-02	1.274905e-02
[942,]	-3.030011e-02	1.248009e-02
[943,]	-3.032096e-02	1.218614e-02
[944,]	-3.021224e-02	1.267513e-02
[945,]	-3.006100e-02	1.338623e-02
[946,]	-3.015430e-02	1.304296e-02
[947,]	-3.031560e-02	1.207279e-02
[948,]	-3.035651e-02	1.172889e-02
[949,]	-3.035765e-02	1.200581e-02
[950,]	-3.033740e-02	1.225686e-02
[951,]	-3.033593e-02	1.221295e-02
[952,]	-3.034816e-02	1.209838e-02
[953,]	-3.037128e-02	1.207598e-02
[954,]	-3.043282e-02	1.178699e-02
[955,]	-3.050818e-02	1.131836e-02
[956,]	-3.053699e-02	1.120785e-02
[957,]	-3.050312e-02	1.140430e-02
[958,]	-3.048698e-02	1.149305e-02
[959,]	-3.053291e-02	1.121993e-02
[960,]	-3.065228e-02	1.069146e-02
[961,]	-3.072677e-02	1.032246e-02
[962,]	-3.074425e-02	1.027756e-02
[963,]	-3.071102e-02	1.032140e-02
[964,]	-3.069914e-02	1.026445e-02

[965,]	-3.076600e-02	1.001072e-02
[966,]	-3.083752e-02	9.615035e-03
[967,]	-3.089011e-02	9.339388e-03
[968,]	-3.091744e-02	9.232565e-03
[969,]	-3.091679e-02	9.137713e-03
[970,]	-3.091199e-02	9.048724e-03
[971,]	-3.091262e-02	9.024422e-03
[972,]	-3.089711e-02	9.060272e-03
[973,]	-3.087490e-02	9.144697e-03
[974,]	-3.090797e-02	9.114011e-03
[975,]	-3.097644e-02	8.695891e-03
[976,]	-3.103670e-02	7.970421e-03
[977,]	-3.106753e-02	7.529533e-03
[978,]	-3.109062e-02	7.414284e-03
[979,]	-3.110045e-02	7.025203e-03
[980,]	-3.114160e-02	6.470360e-03
[981,]	-3.120390e-02	6.232263e-03
[982,]	-3.122714e-02	6.246520e-03
[983,]	-3.123284e-02	6.351450e-03
[984,]	-3.123296e-02	6.459321e-03
[985,]	-3.125884e-02	6.150177e-03
[986,]	-3.129056e-02	5.543072e-03
[987,]	-3.132417e-02	5.164726e-03
[988,]	-3.141194e-02	4.887168e-03
[989,]	-3.143679e-02	4.584692e-03
[990,]	-3.139782e-02	4.493609e-03
[991,]	-3.134343e-02	4.534810e-03
[992,]	-3.130325e-02	4.460906e-03
[993,]	-3.134342e-02	4.230523e-03
[994,]	-3.142976e-02	3.771496e-03
[995,]	-3.145918e-02	3.141249e-03
[996,]	-3.146563e-02	2.772614e-03
[997,]	-3.145194e-02	2.586935e-03
[998,]	-3.142506e-02	2.043514e-03
[999,]	-3.144158e-02	1.385891e-03
[1000,]	-3.145896e-02	9.695415e-04
[1001,]	-3.144935e-02	4.154219e-04
[1002,]	-3.142177e-02	-4.286330e-04
[1003,]	-3.135051e-02	-1.155332e-03
[1004,]	-3.131983e-02	-1.259192e-03
[1005,]	-3.139460e-02	-7.122271e-04
[1006,]	-3.144437e-02	-2.685536e-04

[1007,]	-3.147350e-02	-6.145586e-04
[1008,]	-3.146743e-02	-1.436863e-03
[1009,]	-3.139993e-02	-2.009812e-03
[1010,]	-3.130392e-02	-2.085527e-03
[1011,]	-3.126083e-02	-1.928651e-03
[1012,]	-3.126119e-02	-2.124780e-03
[1013,]	-3.119150e-02	-3.023005e-03
[1014,]	-3.109435e-02	-4.051339e-03
[1015,]	-3.111965e-02	-4.589545e-03
[1016,]	-3.112970e-02	-4.927800e-03
[1017,]	-3.107913e-02	-5.375407e-03
[1018,]	-3.108863e-02	-5.730071e-03
[1019,]	-3.109737e-02	-5.972749e-03
[1020,]	-3.105990e-02	-6.343536e-03
[1021,]	-3.098322e-02	-6.843525e-03
[1022,]	-3.090405e-02	-7.290572e-03
[1023,]	-3.090637e-02	-7.602382e-03
[1024,]	-3.092479e-02	-7.725060e-03
[1025,]	-3.091876e-02	-7.687217e-03
[1026,]	-3.089616e-02	-7.689643e-03
[1027,]	-3.081267e-02	-7.794022e-03
[1028,]	-3.071714e-02	-8.105114e-03
[1029,]	-3.057762e-02	-8.845958e-03
[1030,]	-3.039278e-02	-9.911062e-03
[1031,]	-3.028586e-02	-1.056762e-02
[1032,]	-3.026540e-02	-1.056823e-02
[1033,]	-3.023467e-02	-1.064009e-02
[1034,]	-3.018158e-02	-1.098149e-02
[1035,]	-3.013444e-02	-1.133602e-02
[1036,]	-3.000807e-02	-1.148647e-02
[1037,]	-2.996594e-02	-1.130705e-02
[1038,]	-2.997714e-02	-1.120390e-02
[1039,]	-2.999603e-02	-1.154870e-02
[1040,]	-2.994576e-02	-1.215655e-02
[1041,]	-2.986011e-02	-1.262510e-02
[1042,]	-2.990855e-02	-1.272467e-02
[1043,]	-2.998342e-02	-1.257502e-02
[1044,]	-2.993475e-02	-1.250306e-02
[1045,]	-2.967295e-02	-1.312561e-02
[1046,]	-2.930870e-02	-1.440247e-02
[1047,]	-2.909291e-02	-1.541735e-02
[1048,]	-2.903684e-02	-1.584900e-02

[1049,]	-2.901759e-02	-1.586402e-02
[1050,]	-2.910652e-02	-1.542773e-02
[1051,]	-2.928447e-02	-1.476699e-02
[1052,]	-2.929478e-02	-1.473260e-02
[1053,]	-2.913223e-02	-1.556361e-02
[1054,]	-2.888291e-02	-1.634276e-02
[1055,]	-2.871625e-02	-1.669351e-02
[1056,]	-2.855053e-02	-1.714904e-02
[1057,]	-2.840534e-02	-1.768630e-02
[1058,]	-2.830847e-02	-1.783052e-02
[1059,]	-2.822086e-02	-1.798576e-02
[1060,]	-2.807157e-02	-1.849527e-02
[1061,]	-2.785279e-02	-1.914673e-02
[1062,]	-2.754076e-02	-1.992861e-02
[1063,]	-2.720965e-02	-2.076286e-02
[1064,]	-2.694260e-02	-2.136411e-02
[1065,]	-2.677356e-02	-2.180683e-02
[1066,]	-2.654685e-02	-2.231190e-02
[1067,]	-2.619778e-02	-2.289225e-02
[1068,]	-2.584523e-02	-2.334984e-02
[1069,]	-2.566593e-02	-2.363798e-02
[1070,]	-2.560380e-02	-2.394031e-02
[1071,]	-2.541624e-02	-2.455069e-02
[1072,]	-2.503229e-02	-2.538845e-02
[1073,]	-2.448645e-02	-2.615565e-02
[1074,]	-2.380857e-02	-2.703657e-02
[1075,]	-2.332128e-02	-2.776264e-02
[1076,]	-2.317565e-02	-2.808777e-02
[1077,]	-2.281682e-02	-2.877396e-02
[1078,]	-2.216596e-02	-2.980472e-02
[1079,]	-2.174245e-02	-3.038318e-02
[1080,]	-2.126623e-02	-3.091029e-02
[1081,]	-2.027772e-02	-3.203619e-02
[1082,]	-1.914299e-02	-3.330797e-02
[1083,]	-1.822972e-02	-3.413015e-02
[1084,]	-1.756693e-02	-3.466536e-02
[1085,]	-1.687607e-02	-3.516728e-02
[1086,]	-1.596757e-02	-3.578164e-02
[1087,]	-1.509696e-02	-3.636279e-02
[1088,]	-1.448158e-02	-3.652067e-02
[1089,]	-1.398399e-02	-3.664051e-02
[1090,]	-1.324938e-02	-3.700910e-02

[1091,]	-1.246273e-02	-3.753833e-02
[1092,]	-1.180466e-02	-3.789299e-02
[1093,]	-1.110527e-02	-3.791567e-02
[1094,]	-1.017651e-02	-3.783721e-02
[1095,]	-9.134162e-03	-3.805768e-02
[1096,]	-8.282141e-03	-3.829228e-02
[1097,]	-7.442429e-03	-3.833634e-02
[1098,]	-6.529752e-03	-3.825837e-02
[1099,]	-5.813422e-03	-3.815009e-02
[1100,]	-5.238956e-03	-3.795906e-02
[1101,]	-4.801632e-03	-3.782901e-02
[1102,]	-4.361926e-03	-3.792580e-02
[1103,]	-3.731671e-03	-3.804181e-02
[1104,]	-3.041015e-03	-3.769166e-02
[1105,]	-2.408468e-03	-3.710816e-02
[1106,]	-1.967716e-03	-3.655021e-02
[1107,]	-1.805668e-03	-3.616485e-02
[1108,]	-1.633612e-03	-3.598506e-02
[1109,]	-1.237701e-03	-3.583108e-02
[1110,]	-6.792793e-04	-3.561076e-02
[1111,]	-1.433380e-05	-3.537533e-02
[1112,]	5.105803e-04	-3.522423e-02
[1113,]	7.297315e-04	-3.502463e-02
[1114,]	9.781512e-04	-3.461894e-02
[1115,]	1.383101e-03	-3.377200e-02
[1116,]	1.681476e-03	-3.292753e-02
[1117,]	2.011052e-03	-3.261294e-02
[1118,]	2.706837e-03	-3.247978e-02
[1119,]	3.583029e-03	-3.200381e-02
[1120,]	4.236405e-03	-3.152471e-02
[1121,]	4.537815e-03	-3.138413e-02
[1122,]	4.598160e-03	-3.131230e-02
[1123,]	4.545257e-03	-3.107750e-02
[1124,]	4.580084e-03	-3.082776e-02
[1125,]	4.760627e-03	-3.060029e-02
[1126,]	4.936229e-03	-3.034276e-02
[1127,]	5.155365e-03	-2.992530e-02
[1128,]	5.522876e-03	-2.925854e-02
[1129,]	6.044411e-03	-2.895110e-02
[1130,]	6.432454e-03	-2.928242e-02
[1131,]	6.449808e-03	-2.973166e-02
[1132,]	6.386193e-03	-2.999095e-02

[1133,]	6.469459e-03 -2.977525e-02
[1134,]	6.717833e-03 -2.905416e-02
[1135,]	6.870481e-03 -2.842220e-02
[1136,]	6.987002e-03 -2.835667e-02
[1137,]	7.524576e-03 -2.851339e-02
[1138,]	7.939074e-03 -2.829346e-02
[1139,]	7.737654e-03 -2.762862e-02
[1140,]	7.660768e-03 -2.722011e-02
[1141,]	8.064614e-03 -2.731812e-02
[1142,]	8.391310e-03 -2.724491e-02
[1143,]	8.374259e-03 -2.693812e-02
[1144,]	8.516893e-03 -2.672040e-02
[1145,]	9.074216e-03 -2.655742e-02
[1146,]	9.384112e-03 -2.626685e-02
[1147,]	9.054253e-03 -2.598563e-02
[1148,]	8.696728e-03 -2.596591e-02
[1149,]	8.770587e-03 -2.590325e-02
[1150,]	8.837174e-03 -2.574527e-02
[1151,]	8.653360e-03 -2.559177e-02
[1152,]	8.776056e-03 -2.517177e-02
[1153,]	9.106096e-03 -2.466817e-02
[1154,]	8.905266e-03 -2.461345e-02
[1155,]	8.480798e-03 -2.504289e-02
[1156,]	8.745024e-03 -2.570825e-02
[1157,]	9.648196e-03 -2.615982e-02
[1158,]	1.039838e-02 -2.607033e-02
[1159,]	1.051494e-02 -2.569412e-02
[1160,]	1.019121e-02 -2.547689e-02
[1161,]	9.812640e-03 -2.528892e-02
[1162,]	9.678652e-03 -2.512038e-02
[1163,]	9.795406e-03 -2.537491e-02
[1164,]	9.745333e-03 -2.590410e-02
[1165,]	9.362598e-03 -2.604819e-02
[1166,]	9.119174e-03 -2.568092e-02
[1167,]	9.385546e-03 -2.538771e-02
[1168,]	9.834511e-03 -2.524396e-02
[1169,]	9.983624e-03 -2.507705e-02
[1170,]	9.709798e-03 -2.500695e-02
[1171,]	9.562590e-03 -2.514067e-02
[1172,]	9.945532e-03 -2.543683e-02
[1173,]	1.009632e-02 -2.572761e-02
[1174,]	9.653250e-03 -2.606389e-02

[1175,]	9.366009e-03 -2.638916e-02
[1176,]	9.489722e-03 -2.663632e-02
[1177,]	9.660143e-03 -2.681898e-02
[1178,]	9.661030e-03 -2.669050e-02
[1179,]	9.517807e-03 -2.660825e-02
[1180,]	9.580646e-03 -2.702853e-02
[1181,]	9.735248e-03 -2.724796e-02
[1182,]	9.397421e-03 -2.701489e-02
[1183,]	9.075332e-03 -2.689578e-02
[1184,]	9.399383e-03 -2.683430e-02
[1185,]	9.821952e-03 -2.692234e-02
[1186,]	9.839839e-03 -2.722192e-02
[1187,]	9.837147e-03 -2.728932e-02
[1188,]	9.839255e-03 -2.710505e-02
[1189,]	9.471708e-03 -2.717958e-02
[1190,]	8.999117e-03 -2.760288e-02
[1191,]	8.658330e-03 -2.799533e-02
[1192,]	8.696942e-03 -2.812554e-02
[1193,]	9.090858e-03 -2.788365e-02
[1194,]	9.343457e-03 -2.752100e-02
[1195,]	9.328716e-03 -2.779880e-02
[1196,]	8.937004e-03 -2.859802e-02
[1197,]	8.202841e-03 -2.926665e-02
[1198,]	7.715080e-03 -2.970947e-02
[1199,]	7.812098e-03 -2.986700e-02
[1200,]	7.939838e-03 -2.988559e-02
[1201,]	7.872502e-03 -3.008072e-02
[1202,]	7.876986e-03 -3.050798e-02
[1203,]	7.789640e-03 -3.099942e-02
[1204,]	7.458009e-03 -3.111356e-02
[1205,]	7.189373e-03 -3.092913e-02
[1206,]	7.224416e-03 -3.090971e-02
[1207,]	7.312786e-03 -3.123381e-02
[1208,]	7.118199e-03 -3.166854e-02
[1209,]	6.670268e-03 -3.202795e-02
[1210,]	6.392039e-03 -3.217370e-02
[1211,]	6.655203e-03 -3.199932e-02
[1212,]	6.977331e-03 -3.176392e-02
[1213,]	6.861652e-03 -3.193394e-02
[1214,]	6.835001e-03 -3.246903e-02
[1215,]	7.252742e-03 -3.286804e-02
[1216,]	7.408870e-03 -3.301640e-02

[1217,]	6.925829e-03 -3.322784e-02
[1218,]	6.440713e-03 -3.368276e-02
[1219,]	6.257174e-03 -3.416181e-02
[1220,]	5.929576e-03 -3.429662e-02
[1221,]	5.400435e-03 -3.420458e-02
[1222,]	4.922665e-03 -3.437145e-02
[1223,]	4.601748e-03 -3.485303e-02
[1224,]	4.723501e-03 -3.531745e-02
[1225,]	5.015697e-03 -3.578627e-02
[1226,]	4.825713e-03 -3.640307e-02
[1227,]	4.467107e-03 -3.676987e-02
[1228,]	4.220309e-03 -3.670149e-02
[1229,]	3.933259e-03 -3.674888e-02
[1230,]	3.819026e-03 -3.690238e-02
[1231,]	3.863819e-03 -3.695520e-02
[1232,]	3.546511e-03 -3.718940e-02
[1233,]	2.925889e-03 -3.758158e-02
[1234,]	2.636558e-03 -3.796591e-02
[1235,]	2.686457e-03 -3.809879e-02
[1236,]	2.736741e-03 -3.792544e-02
[1237,]	2.729612e-03 -3.739629e-02
[1238,]	2.583671e-03 -3.723980e-02
[1239,]	2.151767e-03 -3.778064e-02
[1240,]	1.582187e-03 -3.843914e-02
[1241,]	1.355778e-03 -3.879985e-02
[1242,]	1.484164e-03 -3.887860e-02
[1243,]	1.370682e-03 -3.897677e-02
[1244,]	1.011320e-03 -3.930191e-02
[1245,]	8.912621e-04 -3.949231e-02
[1246,]	9.284108e-04 -3.959090e-02
[1247,]	9.475804e-04 -3.956317e-02
[1248,]	9.906133e-04 -3.938326e-02
[1249,]	9.597439e-04 -3.918187e-02
[1250,]	7.266261e-04 -3.921279e-02
[1251,]	1.670970e-04 -3.944156e-02
[1252,]	-6.849205e-04 -3.986811e-02
[1253,]	-1.131320e-03 -4.021039e-02
[1254,]	-8.405134e-04 -4.016425e-02
[1255,]	-5.159359e-04 -3.998831e-02
[1256,]	-4.673633e-04 -4.013584e-02
[1257,]	-4.710476e-04 -4.047747e-02
[1258,]	-5.886816e-04 -4.073194e-02

[1259,] -9.897994e-04 -4.088039e-02
[1260,] -1.602345e-03 -4.104258e-02
[1261,] -2.033314e-03 -4.122498e-02
[1262,] -2.046020e-03 -4.130021e-02
[1263,] -1.954877e-03 -4.138802e-02
[1264,] -2.036300e-03 -4.145299e-02
[1265,] -2.274602e-03 -4.155261e-02
[1266,] -2.708013e-03 -4.161584e-02
[1267,] -3.091513e-03 -4.148984e-02
[1268,] -3.098185e-03 -4.130159e-02
[1269,] -2.840729e-03 -4.117252e-02
[1270,] -2.499748e-03 -4.119714e-02
[1271,] -2.179456e-03 -4.139207e-02
[1272,] -2.178854e-03 -4.157091e-02
[1273,] -2.704143e-03 -4.164273e-02
[1274,] -3.432471e-03 -4.167792e-02
[1275,] -3.749702e-03 -4.168484e-02
[1276,] -3.611463e-03 -4.160704e-02
[1277,] -3.728695e-03 -4.161245e-02
[1278,] -4.304195e-03 -4.170550e-02
[1279,] -4.777452e-03 -4.178240e-02
[1280,] -4.909680e-03 -4.177864e-02
[1281,] -5.069388e-03 -4.164813e-02
[1282,] -5.459981e-03 -4.161823e-02
[1283,] -5.691189e-03 -4.168819e-02
[1284,] -5.497060e-03 -4.165195e-02
[1285,] -5.553842e-03 -4.151264e-02
[1286,] -6.292900e-03 -4.134400e-02
[1287,] -6.717286e-03 -4.136724e-02
[1288,] -6.470241e-03 -4.143951e-02
[1289,] -6.169878e-03 -4.130345e-02
[1290,] -5.950777e-03 -4.143260e-02
[1291,] -6.001274e-03 -4.165444e-02
[1292,] -6.486713e-03 -4.160594e-02
[1293,] -7.186531e-03 -4.132017e-02
[1294,] -7.795829e-03 -4.106914e-02
[1295,] -8.050096e-03 -4.107709e-02
[1296,] -8.159723e-03 -4.115809e-02
[1297,] -8.295782e-03 -4.110470e-02
[1298,] -8.366841e-03 -4.117369e-02
[1299,] -8.415275e-03 -4.119173e-02
[1300,] -8.487570e-03 -4.109173e-02

[1301,]	-8.509115e-03	-4.091825e-02
[1302,]	-8.653232e-03	-4.091174e-02
[1303,]	-9.082617e-03	-4.079595e-02
[1304,]	-9.555265e-03	-4.051841e-02
[1305,]	-9.609905e-03	-4.049979e-02
[1306,]	-9.348561e-03	-4.068761e-02
[1307,]	-9.310932e-03	-4.066764e-02
[1308,]	-9.474846e-03	-4.056276e-02
[1309,]	-9.501691e-03	-4.056862e-02
[1310,]	-9.545286e-03	-4.050786e-02
[1311,]	-1.002006e-02	-4.018350e-02
[1312,]	-1.068495e-02	-3.981258e-02
[1313,]	-1.084775e-02	-3.973536e-02
[1314,]	-1.053118e-02	-3.988266e-02
[1315,]	-1.024309e-02	-4.001203e-02
[1316,]	-1.012535e-02	-3.992998e-02
[1317,]	-1.010902e-02	-3.977066e-02
[1318,]	-1.008184e-02	-3.979776e-02
[1319,]	-1.018085e-02	-3.994606e-02
[1320,]	-1.065078e-02	-3.990958e-02
[1321,]	-1.114209e-02	-3.966346e-02
[1322,]	-1.126639e-02	-3.945992e-02
[1323,]	-1.123712e-02	-3.928393e-02
[1324,]	-1.130118e-02	-3.917758e-02
[1325,]	-1.148676e-02	-3.911214e-02
[1326,]	-1.191303e-02	-3.880819e-02
[1327,]	-1.249557e-02	-3.837148e-02
[1328,]	-1.291113e-02	-3.806143e-02
[1329,]	-1.297026e-02	-3.795782e-02
[1330,]	-1.272324e-02	-3.803675e-02
[1331,]	-1.264672e-02	-3.814679e-02
[1332,]	-1.291036e-02	-3.809794e-02
[1333,]	-1.299504e-02	-3.802519e-02
[1334,]	-1.280204e-02	-3.811295e-02
[1335,]	-1.258432e-02	-3.828881e-02
[1336,]	-1.240307e-02	-3.828344e-02
[1337,]	-1.256279e-02	-3.795124e-02
[1338,]	-1.303936e-02	-3.751135e-02
[1339,]	-1.342619e-02	-3.719293e-02
[1340,]	-1.351390e-02	-3.712151e-02
[1341,]	-1.335893e-02	-3.712767e-02
[1342,]	-1.321295e-02	-3.712256e-02

[1343,]	-1.313970e-02	-3.719847e-02
[1344,]	-1.309290e-02	-3.725006e-02
[1345,]	-1.316428e-02	-3.698060e-02
[1346,]	-1.347509e-02	-3.652296e-02
[1347,]	-1.385514e-02	-3.616384e-02
[1348,]	-1.412540e-02	-3.602012e-02
[1349,]	-1.425875e-02	-3.622474e-02
[1350,]	-1.424792e-02	-3.655806e-02
[1351,]	-1.428249e-02	-3.651711e-02
[1352,]	-1.449159e-02	-3.617036e-02
[1353,]	-1.468466e-02	-3.579732e-02
[1354,]	-1.484610e-02	-3.544287e-02
[1355,]	-1.512849e-02	-3.508712e-02
[1356,]	-1.525569e-02	-3.498112e-02
[1357,]	-1.507222e-02	-3.509014e-02
[1358,]	-1.492387e-02	-3.512295e-02
[1359,]	-1.491855e-02	-3.510866e-02
[1360,]	-1.484473e-02	-3.511566e-02
[1361,]	-1.469465e-02	-3.514042e-02
[1362,]	-1.478093e-02	-3.490889e-02
[1363,]	-1.517952e-02	-3.454337e-02
[1364,]	-1.561720e-02	-3.436290e-02
[1365,]	-1.597635e-02	-3.429284e-02
[1366,]	-1.603827e-02	-3.415637e-02
[1367,]	-1.582443e-02	-3.403265e-02
[1368,]	-1.582404e-02	-3.397915e-02
[1369,]	-1.600526e-02	-3.394908e-02
[1370,]	-1.602319e-02	-3.405752e-02
[1371,]	-1.597160e-02	-3.402627e-02
[1372,]	-1.609798e-02	-3.362224e-02
[1373,]	-1.634783e-02	-3.327303e-02
[1374,]	-1.650006e-02	-3.326956e-02
[1375,]	-1.650256e-02	-3.339189e-02
[1376,]	-1.644699e-02	-3.344735e-02
[1377,]	-1.643235e-02	-3.344779e-02
[1378,]	-1.652089e-02	-3.329361e-02
[1379,]	-1.660312e-02	-3.301523e-02
[1380,]	-1.665816e-02	-3.283810e-02
[1381,]	-1.688161e-02	-3.258036e-02
[1382,]	-1.722970e-02	-3.203453e-02
[1383,]	-1.746168e-02	-3.150354e-02
[1384,]	-1.743936e-02	-3.153920e-02

[1385,] -1.730701e-02 -3.217806e-02
[1386,] -1.730707e-02 -3.254263e-02
[1387,] -1.724466e-02 -3.238938e-02
[1388,] -1.691783e-02 -3.218577e-02
[1389,] -1.685012e-02 -3.193678e-02
[1390,] -1.709473e-02 -3.177599e-02
[1391,] -1.710746e-02 -3.188265e-02
[1392,] -1.692801e-02 -3.193050e-02
[1393,] -1.671568e-02 -3.202707e-02
[1394,] -1.637906e-02 -3.224497e-02
[1395,] -1.629741e-02 -3.220867e-02
[1396,] -1.648901e-02 -3.191774e-02
[1397,] -1.654017e-02 -3.180796e-02
[1398,] -1.662654e-02 -3.184886e-02
[1399,] -1.683695e-02 -3.170212e-02
[1400,] -1.696994e-02 -3.134406e-02
[1401,] -1.688562e-02 -3.107590e-02
[1402,] -1.656323e-02 -3.113156e-02
[1403,] -1.634449e-02 -3.128894e-02
[1404,] -1.618314e-02 -3.158672e-02
[1405,] -1.585644e-02 -3.203982e-02
[1406,] -1.587063e-02 -3.217979e-02
[1407,] -1.633680e-02 -3.179107e-02
[1408,] -1.661283e-02 -3.148960e-02
[1409,] -1.649994e-02 -3.150034e-02
[1410,] -1.629593e-02 -3.148096e-02
[1411,] -1.616839e-02 -3.146373e-02
[1412,] -1.626698e-02 -3.129970e-02
[1413,] -1.648522e-02 -3.107403e-02
[1414,] -1.627000e-02 -3.121843e-02
[1415,] -1.568229e-02 -3.160357e-02
[1416,] -1.539991e-02 -3.169837e-02
[1417,] -1.556772e-02 -3.147633e-02
[1418,] -1.571842e-02 -3.137541e-02
[1419,] -1.560500e-02 -3.157633e-02
[1420,] -1.560717e-02 -3.187049e-02
[1421,] -1.601097e-02 -3.191513e-02
[1422,] -1.635368e-02 -3.180310e-02
[1423,] -1.613566e-02 -3.178097e-02
[1424,] -1.557107e-02 -3.179111e-02
[1425,] -1.515695e-02 -3.181075e-02
[1426,] -1.508991e-02 -3.183583e-02

[1427,]	-1.523099e-02	-3.171635e-02
[1428,]	-1.529791e-02	-3.155283e-02
[1429,]	-1.514330e-02	-3.165584e-02
[1430,]	-1.490115e-02	-3.205610e-02
[1431,]	-1.479844e-02	-3.231397e-02
[1432,]	-1.486067e-02	-3.216870e-02
[1433,]	-1.499545e-02	-3.192575e-02
[1434,]	-1.502938e-02	-3.193358e-02
[1435,]	-1.481795e-02	-3.208574e-02
[1436,]	-1.444973e-02	-3.204973e-02
[1437,]	-1.419353e-02	-3.191777e-02
[1438,]	-1.411171e-02	-3.195955e-02
[1439,]	-1.392556e-02	-3.209615e-02
[1440,]	-1.366289e-02	-3.220333e-02
[1441,]	-1.348294e-02	-3.239284e-02
[1442,]	-1.327030e-02	-3.272452e-02
[1443,]	-1.310846e-02	-3.286739e-02
[1444,]	-1.308207e-02	-3.287135e-02
[1445,]	-1.293470e-02	-3.299155e-02
[1446,]	-1.268258e-02	-3.324832e-02
[1447,]	-1.246074e-02	-3.349942e-02
[1448,]	-1.232523e-02	-3.347872e-02
[1449,]	-1.222314e-02	-3.323609e-02
[1450,]	-1.209927e-02	-3.296871e-02
[1451,]	-1.190904e-02	-3.280237e-02
[1452,]	-1.159168e-02	-3.301057e-02
[1453,]	-1.131380e-02	-3.344818e-02
[1454,]	-1.121097e-02	-3.369524e-02
[1455,]	-1.115939e-02	-3.368975e-02
[1456,]	-1.093450e-02	-3.370551e-02
[1457,]	-1.059564e-02	-3.394296e-02
[1458,]	-1.048431e-02	-3.418827e-02
[1459,]	-1.044858e-02	-3.431885e-02
[1460,]	-1.013883e-02	-3.438398e-02
[1461,]	-9.650251e-03	-3.437607e-02
[1462,]	-9.262256e-03	-3.447260e-02
[1463,]	-9.136817e-03	-3.466557e-02
[1464,]	-9.097455e-03	-3.481457e-02
[1465,]	-8.867995e-03	-3.493414e-02
[1466,]	-8.628189e-03	-3.501506e-02
[1467,]	-8.770027e-03	-3.512349e-02
[1468,]	-9.116860e-03	-3.528796e-02

[1469,]	-9.129204e-03	-3.538730e-02
[1470,]	-8.714715e-03	-3.549749e-02
[1471,]	-8.213907e-03	-3.556377e-02
[1472,]	-8.008217e-03	-3.547777e-02
[1473,]	-8.033307e-03	-3.533969e-02
[1474,]	-7.876895e-03	-3.544241e-02
[1475,]	-7.562596e-03	-3.569343e-02
[1476,]	-7.413431e-03	-3.563253e-02
[1477,]	-7.199855e-03	-3.538350e-02
[1478,]	-6.680237e-03	-3.527622e-02
[1479,]	-6.302453e-03	-3.536698e-02
[1480,]	-6.233151e-03	-3.556294e-02
[1481,]	-6.010005e-03	-3.563895e-02
[1482,]	-5.621762e-03	-3.570276e-02
[1483,]	-5.389238e-03	-3.575191e-02
[1484,]	-5.383953e-03	-3.572733e-02
[1485,]	-5.623632e-03	-3.569379e-02
[1486,]	-5.996353e-03	-3.550046e-02
[1487,]	-6.190566e-03	-3.526425e-02
[1488,]	-6.186453e-03	-3.520455e-02
[1489,]	-6.160735e-03	-3.504045e-02
[1490,]	-5.906157e-03	-3.499130e-02
[1491,]	-5.254725e-03	-3.516665e-02
[1492,]	-4.761165e-03	-3.528336e-02
[1493,]	-4.883218e-03	-3.521556e-02
[1494,]	-5.184735e-03	-3.501199e-02
[1495,]	-5.154546e-03	-3.460310e-02
[1496,]	-4.952778e-03	-3.444775e-02
[1497,]	-5.005032e-03	-3.467789e-02
[1498,]	-5.397544e-03	-3.480532e-02
[1499,]	-5.562238e-03	-3.448121e-02
[1500,]	-5.203395e-03	-3.412756e-02
[1501,]	-5.074219e-03	-3.397817e-02
[1502,]	-5.564654e-03	-3.407720e-02
[1503,]	-5.846377e-03	-3.411160e-02
[1504,]	-5.595156e-03	-3.380065e-02
[1505,]	-5.412447e-03	-3.353928e-02
[1506,]	-5.397949e-03	-3.352030e-02
[1507,]	-5.191946e-03	-3.363577e-02
[1508,]	-4.969501e-03	-3.372908e-02
[1509,]	-5.179202e-03	-3.361816e-02
[1510,]	-5.605988e-03	-3.328930e-02

[1511,]	-5.760961e-03	-3.302506e-02
[1512,]	-5.576187e-03	-3.289812e-02
[1513,]	-5.442449e-03	-3.279151e-02
[1514,]	-5.453816e-03	-3.257852e-02
[1515,]	-5.153705e-03	-3.229111e-02
[1516,]	-4.766811e-03	-3.215731e-02
[1517,]	-4.977629e-03	-3.208934e-02
[1518,]	-5.441351e-03	-3.201452e-02
[1519,]	-5.302817e-03	-3.222815e-02
[1520,]	-4.743893e-03	-3.261259e-02
[1521,]	-4.550774e-03	-3.276059e-02
[1522,]	-4.722332e-03	-3.259687e-02
[1523,]	-4.623944e-03	-3.229792e-02
[1524,]	-4.238948e-03	-3.207938e-02
[1525,]	-3.988266e-03	-3.194686e-02
[1526,]	-3.888334e-03	-3.180877e-02
[1527,]	-3.804186e-03	-3.187914e-02
[1528,]	-3.949081e-03	-3.237931e-02
[1529,]	-4.326633e-03	-3.277477e-02
[1530,]	-4.295287e-03	-3.287076e-02
[1531,]	-3.593837e-03	-3.309419e-02
[1532,]	-2.956666e-03	-3.348228e-02
[1533,]	-2.669847e-03	-3.382415e-02
[1534,]	-2.145316e-03	-3.380358e-02
[1535,]	-1.391101e-03	-3.351758e-02
[1536,]	-9.736618e-04	-3.325497e-02
[1537,]	-7.405487e-04	-3.309727e-02
[1538,]	-3.561204e-04	-3.319647e-02
[1539,]	8.311213e-05	-3.361901e-02
[1540,]	5.486207e-04	-3.389036e-02
[1541,]	1.223881e-03	-3.395531e-02
[1542,]	2.345761e-03	-3.401894e-02
[1543,]	3.505334e-03	-3.386143e-02
[1544,]	4.231981e-03	-3.345815e-02
[1545,]	5.131243e-03	-3.322045e-02
[1546,]	6.433158e-03	-3.317688e-02
[1547,]	7.573511e-03	-3.302542e-02
[1548,]	8.757131e-03	-3.261315e-02
[1549,]	1.040868e-02	-3.200612e-02
[1550,]	1.198513e-02	-3.133528e-02
[1551,]	1.362731e-02	-3.030730e-02
[1552,]	1.548590e-02	-2.863996e-02

[1553,]	1.700916e-02	-2.703701e-02
[1554,]	1.862371e-02	-2.550028e-02
[1555,]	2.032958e-02	-2.348698e-02
[1556,]	2.210870e-02	-2.079945e-02
[1557,]	2.400084e-02	-1.755828e-02
[1558,]	2.546387e-02	-1.442125e-02
[1559,]	2.657182e-02	-1.104035e-02
[1560,]	2.774854e-02	-7.530837e-03
[1561,]	2.874461e-02	-4.522461e-03
[1562,]	2.920786e-02	-1.634344e-03
[1563,]	2.933288e-02	1.201780e-03
[1564,]	2.928523e-02	3.658838e-03
[1565,]	2.904733e-02	6.303074e-03
[1566,]	2.851439e-02	9.453096e-03
[1567,]	2.776503e-02	1.211391e-02
[1568,]	2.703541e-02	1.365137e-02
[1569,]	2.634638e-02	1.461907e-02
[1570,]	2.566810e-02	1.559847e-02
[1571,]	2.501071e-02	1.666998e-02
[1572,]	2.439023e-02	1.771636e-02
[1573,]	2.378707e-02	1.863011e-02
[1574,]	2.314083e-02	1.939830e-02
[1575,]	2.249943e-02	2.003519e-02
[1576,]	2.204143e-02	2.044470e-02
[1577,]	2.183242e-02	2.068280e-02
[1578,]	2.170468e-02	2.094432e-02
[1579,]	2.155330e-02	2.120619e-02
[1580,]	2.141147e-02	2.133670e-02
[1581,]	2.128959e-02	2.145454e-02
[1582,]	2.112141e-02	2.167400e-02
[1583,]	2.083196e-02	2.183216e-02
[1584,]	2.043883e-02	2.191251e-02
[1585,]	2.006902e-02	2.214077e-02
[1586,]	1.989485e-02	2.245320e-02
[1587,]	1.996707e-02	2.253514e-02
[1588,]	2.008839e-02	2.246396e-02
[1589,]	2.011149e-02	2.248859e-02
[1590,]	2.012029e-02	2.254733e-02
[1591,]	2.018827e-02	2.268698e-02
[1592,]	2.024668e-02	2.293207e-02
[1593,]	2.018399e-02	2.302210e-02
[1594,]	2.005091e-02	2.291524e-02

[1595,]	2.006247e-02	2.283610e-02
[1596,]	2.019410e-02	2.269634e-02
[1597,]	2.025736e-02	2.250412e-02
[1598,]	2.022642e-02	2.253831e-02
[1599,]	2.030137e-02	2.271973e-02
[1600,]	2.051531e-02	2.269453e-02
[1601,]	2.064331e-02	2.251593e-02
[1602,]	2.065722e-02	2.252457e-02
[1603,]	2.076475e-02	2.269138e-02
[1604,]	2.096688e-02	2.269951e-02
[1605,]	2.113709e-02	2.252667e-02
[1606,]	2.121958e-02	2.244405e-02
[1607,]	2.124033e-02	2.253209e-02
[1608,]	2.131052e-02	2.270058e-02
[1609,]	2.147225e-02	2.278041e-02
[1610,]	2.168913e-02	2.257956e-02
[1611,]	2.195457e-02	2.219260e-02
[1612,]	2.223830e-02	2.189810e-02
[1613,]	2.238090e-02	2.191885e-02
[1614,]	2.241391e-02	2.211380e-02
[1615,]	2.248919e-02	2.215414e-02
[1616,]	2.258885e-02	2.202841e-02
[1617,]	2.274709e-02	2.184527e-02
[1618,]	2.295994e-02	2.168814e-02
[1619,]	2.315486e-02	2.176923e-02
[1620,]	2.332622e-02	2.206278e-02
[1621,]	2.353565e-02	2.211916e-02
[1622,]	2.384182e-02	2.174800e-02
[1623,]	2.409343e-02	2.133515e-02
[1624,]	2.418949e-02	2.115496e-02
[1625,]	2.429700e-02	2.103211e-02
[1626,]	2.452773e-02	2.088548e-02
[1627,]	2.477232e-02	2.088551e-02
[1628,]	2.493383e-02	2.105657e-02
[1629,]	2.504421e-02	2.116634e-02
[1630,]	2.517206e-02	2.099707e-02
[1631,]	2.535835e-02	2.063711e-02
[1632,]	2.553914e-02	2.040303e-02
[1633,]	2.567254e-02	2.047943e-02
[1634,]	2.583937e-02	2.059340e-02
[1635,]	2.605772e-02	2.035137e-02
[1636,]	2.627463e-02	1.981438e-02

[1637,]	2.647044e-02	1.939666e-02
[1638,]	2.666330e-02	1.924191e-02
[1639,]	2.692377e-02	1.895446e-02
[1640,]	2.723248e-02	1.843614e-02
[1641,]	2.742323e-02	1.819211e-02
[1642,]	2.743609e-02	1.834751e-02
[1643,]	2.738827e-02	1.850846e-02
[1644,]	2.739311e-02	1.846604e-02
[1645,]	2.752504e-02	1.826576e-02
[1646,]	2.775635e-02	1.807540e-02
[1647,]	2.793065e-02	1.800608e-02
[1648,]	2.810101e-02	1.788167e-02
[1649,]	2.831780e-02	1.759082e-02
[1650,]	2.848521e-02	1.733119e-02
[1651,]	2.851548e-02	1.729130e-02
[1652,]	2.852721e-02	1.738666e-02
[1653,]	2.859751e-02	1.733953e-02
[1654,]	2.863712e-02	1.722807e-02
[1655,]	2.864346e-02	1.711600e-02
[1656,]	2.875115e-02	1.672172e-02
[1657,]	2.893622e-02	1.625277e-02
[1658,]	2.904177e-02	1.609280e-02
[1659,]	2.908704e-02	1.614532e-02
[1660,]	2.911113e-02	1.621524e-02
[1661,]	2.913375e-02	1.612898e-02
[1662,]	2.919162e-02	1.571190e-02
[1663,]	2.926041e-02	1.534326e-02
[1664,]	2.929134e-02	1.533334e-02
[1665,]	2.930589e-02	1.529137e-02
[1666,]	2.935258e-02	1.511285e-02
[1667,]	2.936318e-02	1.508571e-02
[1668,]	2.937218e-02	1.511753e-02
[1669,]	2.942922e-02	1.494780e-02
[1670,]	2.947288e-02	1.470598e-02
[1671,]	2.948338e-02	1.460390e-02
[1672,]	2.951840e-02	1.453490e-02
[1673,]	2.956901e-02	1.440718e-02
[1674,]	2.953446e-02	1.426815e-02
[1675,]	2.941795e-02	1.419829e-02
[1676,]	2.938909e-02	1.403102e-02
[1677,]	2.949424e-02	1.356783e-02
[1678,]	2.957048e-02	1.305649e-02

[1679,]	2.956166e-02	1.289738e-02
[1680,]	2.947119e-02	1.317020e-02
[1681,]	2.931294e-02	1.363920e-02
[1682,]	2.916487e-02	1.396637e-02
[1683,]	2.906753e-02	1.405357e-02
[1684,]	2.902118e-02	1.390724e-02
[1685,]	2.903295e-02	1.358991e-02
[1686,]	2.907198e-02	1.335575e-02
[1687,]	2.910105e-02	1.325241e-02
[1688,]	2.906766e-02	1.308235e-02
[1689,]	2.894298e-02	1.299275e-02
[1690,]	2.881800e-02	1.305632e-02
[1691,]	2.879029e-02	1.298065e-02
[1692,]	2.883463e-02	1.268628e-02
[1693,]	2.877933e-02	1.247956e-02
[1694,]	2.859869e-02	1.247599e-02
[1695,]	2.840689e-02	1.245052e-02
[1696,]	2.822829e-02	1.245400e-02
[1697,]	2.807920e-02	1.262397e-02
[1698,]	2.806304e-02	1.261293e-02
[1699,]	2.817733e-02	1.206382e-02
[1700,]	2.827618e-02	1.124511e-02
[1701,]	2.829069e-02	1.072295e-02
[1702,]	2.816805e-02	1.078225e-02
[1703,]	2.798358e-02	1.115776e-02
[1704,]	2.772416e-02	1.160480e-02
[1705,]	2.752405e-02	1.208123e-02
[1706,]	2.756973e-02	1.223115e-02
[1707,]	2.760000e-02	1.198678e-02
[1708,]	2.742697e-02	1.162692e-02
[1709,]	2.715568e-02	1.135916e-02
[1710,]	2.690144e-02	1.128627e-02
[1711,]	2.676650e-02	1.130993e-02
[1712,]	2.679818e-02	1.132175e-02
[1713,]	2.693863e-02	1.145909e-02
[1714,]	2.688437e-02	1.155823e-02
[1715,]	2.659214e-02	1.132931e-02
[1716,]	2.626520e-02	1.121728e-02
[1717,]	2.606316e-02	1.144260e-02
[1718,]	2.606502e-02	1.136947e-02
[1719,]	2.625710e-02	1.075234e-02
[1720,]	2.637948e-02	1.020546e-02

[1721,]	2.627382e-02	1.014540e-02
[1722,]	2.606029e-02	1.013973e-02
[1723,]	2.588894e-02	9.855508e-03
[1724,]	2.581308e-02	9.603250e-03
[1725,]	2.571925e-02	9.414844e-03
[1726,]	2.555787e-02	9.106482e-03
[1727,]	2.547104e-02	8.936967e-03
[1728,]	2.547297e-02	9.183660e-03
[1729,]	2.542796e-02	9.549576e-03
[1730,]	2.529551e-02	9.566106e-03
[1731,]	2.515112e-02	9.187851e-03
[1732,]	2.495984e-02	8.869262e-03
[1733,]	2.468097e-02	8.901289e-03
[1734,]	2.446275e-02	9.004454e-03
[1735,]	2.429895e-02	8.873802e-03
[1736,]	2.406118e-02	8.654477e-03
[1737,]	2.377892e-02	8.517401e-03
[1738,]	2.364303e-02	8.499417e-03
[1739,]	2.361860e-02	8.587514e-03
[1740,]	2.352506e-02	8.648254e-03
[1741,]	2.333948e-02	8.778589e-03
[1742,]	2.313521e-02	9.020622e-03
[1743,]	2.291434e-02	9.221987e-03
[1744,]	2.274618e-02	9.351204e-03
[1745,]	2.275629e-02	9.170540e-03
[1746,]	2.288409e-02	8.581068e-03
[1747,]	2.294073e-02	8.028443e-03
[1748,]	2.287652e-02	7.743825e-03
[1749,]	2.279587e-02	7.445832e-03
[1750,]	2.269080e-02	6.897286e-03
[1751,]	2.255727e-02	6.455304e-03
[1752,]	2.244590e-02	6.690911e-03
[1753,]	2.225864e-02	7.403291e-03
[1754,]	2.206672e-02	7.888469e-03
[1755,]	2.197645e-02	7.834325e-03
[1756,]	2.188841e-02	7.447349e-03
[1757,]	2.184584e-02	7.131238e-03
[1758,]	2.180374e-02	7.200731e-03
[1759,]	2.169912e-02	7.570566e-03
[1760,]	2.162861e-02	7.717613e-03
[1761,]	2.168351e-02	7.449761e-03
[1762,]	2.187828e-02	7.146643e-03

[1763,]	2.201675e-02	7.227930e-03
[1764,]	2.186830e-02	7.834035e-03
[1765,]	2.166496e-02	8.591131e-03
[1766,]	2.172373e-02	8.847564e-03
[1767,]	2.183970e-02	8.687431e-03
[1768,]	2.179893e-02	8.651362e-03
[1769,]	2.173746e-02	8.779177e-03
[1770,]	2.176019e-02	8.894541e-03
[1771,]	2.192834e-02	9.010542e-03
[1772,]	2.220591e-02	9.194326e-03
[1773,]	2.250374e-02	9.381954e-03
[1774,]	2.271041e-02	9.490526e-03
[1775,]	2.272682e-02	9.693621e-03
[1776,]	2.271917e-02	1.013774e-02
[1777,]	2.287946e-02	1.059916e-02
[1778,]	2.306520e-02	1.086690e-02
[1779,]	2.308492e-02	1.110305e-02
[1780,]	2.300203e-02	1.149510e-02
[1781,]	2.295400e-02	1.193789e-02
[1782,]	2.293853e-02	1.229377e-02
[1783,]	2.290516e-02	1.267234e-02
[1784,]	2.285615e-02	1.310064e-02
[1785,]	2.290614e-02	1.333509e-02
[1786,]	2.300719e-02	1.339958e-02
[1787,]	2.299203e-02	1.368485e-02
[1788,]	2.298074e-02	1.417068e-02
[1789,]	2.319531e-02	1.447382e-02
[1790,]	2.349137e-02	1.461069e-02
[1791,]	2.353268e-02	1.483278e-02
[1792,]	2.337220e-02	1.506059e-02
[1793,]	2.331042e-02	1.514210e-02
[1794,]	2.343975e-02	1.515267e-02
[1795,]	2.363776e-02	1.524227e-02
[1796,]	2.375814e-02	1.539156e-02
[1797,]	2.380226e-02	1.547102e-02
[1798,]	2.378643e-02	1.567059e-02
[1799,]	2.378402e-02	1.625932e-02
[1800,]	2.381455e-02	1.702910e-02
[1801,]	2.374098e-02	1.760240e-02
[1802,]	2.366212e-02	1.792806e-02
[1803,]	2.375836e-02	1.813770e-02
[1804,]	2.396586e-02	1.837162e-02

[1805,]	2.413491e-02	1.860310e-02
[1806,]	2.436222e-02	1.846769e-02
[1807,]	2.467367e-02	1.797595e-02
[1808,]	2.478137e-02	1.783666e-02
[1809,]	2.463576e-02	1.815289e-02
[1810,]	2.457308e-02	1.846046e-02
[1811,]	2.456643e-02	1.877728e-02
[1812,]	2.444561e-02	1.925683e-02
[1813,]	2.437123e-02	1.975145e-02
[1814,]	2.447198e-02	2.002966e-02
[1815,]	2.457788e-02	2.014206e-02
[1816,]	2.453804e-02	2.029568e-02
[1817,]	2.446820e-02	2.051213e-02
[1818,]	2.453107e-02	2.070066e-02
[1819,]	2.462928e-02	2.089983e-02
[1820,]	2.457653e-02	2.121137e-02
[1821,]	2.446987e-02	2.154452e-02
[1822,]	2.451134e-02	2.171550e-02
[1823,]	2.467723e-02	2.171204e-02
[1824,]	2.483508e-02	2.158638e-02
[1825,]	2.495026e-02	2.151715e-02
[1826,]	2.498175e-02	2.164404e-02
[1827,]	2.490504e-02	2.181943e-02
[1828,]	2.481365e-02	2.191843e-02
[1829,]	2.471748e-02	2.214162e-02
[1830,]	2.458433e-02	2.251047e-02
[1831,]	2.449860e-02	2.277632e-02
[1832,]	2.455397e-02	2.288782e-02
[1833,]	2.466496e-02	2.294800e-02
[1834,]	2.471135e-02	2.301594e-02
[1835,]	2.468085e-02	2.316614e-02
[1836,]	2.462038e-02	2.341683e-02
[1837,]	2.457209e-02	2.365076e-02
[1838,]	2.459942e-02	2.372907e-02
[1839,]	2.474043e-02	2.362491e-02
[1840,]	2.490649e-02	2.352287e-02
[1841,]	2.494256e-02	2.359037e-02
[1842,]	2.484706e-02	2.375098e-02
[1843,]	2.473252e-02	2.389534e-02
[1844,]	2.468161e-02	2.407129e-02
[1845,]	2.471621e-02	2.426739e-02
[1846,]	2.474733e-02	2.445667e-02

[1847,]	2.468174e-02	2.469108e-02
[1848,]	2.459170e-02	2.490815e-02
[1849,]	2.458495e-02	2.494935e-02
[1850,]	2.465462e-02	2.478169e-02
[1851,]	2.467212e-02	2.467578e-02
[1852,]	2.458722e-02	2.479726e-02
[1853,]	2.448827e-02	2.502168e-02
[1854,]	2.430960e-02	2.545258e-02
[1855,]	2.403939e-02	2.603925e-02
[1856,]	2.387922e-02	2.647148e-02
[1857,]	2.388651e-02	2.664248e-02
[1858,]	2.403991e-02	2.654939e-02
[1859,]	2.410907e-02	2.650478e-02
[1860,]	2.391064e-02	2.677731e-02
[1861,]	2.369119e-02	2.711365e-02
[1862,]	2.361800e-02	2.728621e-02
[1863,]	2.360640e-02	2.735163e-02
[1864,]	2.360578e-02	2.740713e-02
[1865,]	2.356590e-02	2.750002e-02
[1866,]	2.351469e-02	2.758976e-02
[1867,]	2.346588e-02	2.762216e-02
[1868,]	2.335701e-02	2.764867e-02
[1869,]	2.325228e-02	2.772846e-02
[1870,]	2.319792e-02	2.789401e-02
[1871,]	2.307020e-02	2.817261e-02
[1872,]	2.280823e-02	2.848498e-02
[1873,]	2.259343e-02	2.865489e-02
[1874,]	2.253646e-02	2.869177e-02
[1875,]	2.249426e-02	2.875840e-02
[1876,]	2.240233e-02	2.887250e-02
[1877,]	2.232024e-02	2.895343e-02
[1878,]	2.226614e-02	2.898108e-02
[1879,]	2.215426e-02	2.903166e-02
[1880,]	2.195290e-02	2.916747e-02
[1881,]	2.184576e-02	2.925356e-02
[1882,]	2.188910e-02	2.922155e-02
[1883,]	2.187461e-02	2.920049e-02
[1884,]	2.179267e-02	2.918976e-02
[1885,]	2.178995e-02	2.907173e-02
[1886,]	2.181044e-02	2.896669e-02
[1887,]	2.173147e-02	2.903530e-02
[1888,]	2.166047e-02	2.924486e-02

[1889,]	2.166776e-02	2.939192e-02
[1890,]	2.166670e-02	2.928573e-02
[1891,]	2.170072e-02	2.890044e-02
[1892,]	2.178735e-02	2.855123e-02
[1893,]	2.165330e-02	2.872549e-02
[1894,]	2.131812e-02	2.927262e-02
[1895,]	2.135178e-02	2.933728e-02
[1896,]	2.171629e-02	2.876925e-02
[1897,]	2.184198e-02	2.855235e-02
[1898,]	2.191548e-02	2.871375e-02
[1899,]	2.239582e-02	2.834403e-02

Table S2. Instrumental texture analysis and TSS parameters.

Training set				
**Sample**	**DiamEqu**	**Hardness (N)**	**TSS (°Brix) ‡**	
T1_1	18.6	19.12	19.6±0.2	l
T1_2	17.0	14.58	18.5±0.1	g
T1_3	17.6	8.70	16.6±0.2	d
T1_4	19.7	14.21	18.7±0.2	h
T1_5	21.1	14.93	18.2±0.1	g
T1_6	17.4	9.55	16.7±0.2	d
T1_7	18.7	13.58	17.7±0.2	f
T1_8	22.8	17.12	18.3±0.1	g
T1_9	17.3	8.08	19.2±0.1	i
T1_10	23	17.96	16.6±0.2	d
T1_11	17.6	9.45	17.7±0.2	f
T1_12	18.1	12.83	17.2±0.1	e
T1_13	18.7	16.86	18.1±0.1	g
T1_14	19.3	12.68	21.5±0.1	o
T1_15	17.9	12.11	18.1±0.1	g
T1_16	20	16.40	17.2±0.1	e
T1_17	18.6	13.23	19.3±0.1	i
T1_18	19.6	14.38	18.2±0.1	g
T1_19	18.8	13.12	22.7±0.2	q
T1_20	18.4	9.27	19.4±0.1	i
T1_21	19.1	14.36	17.1±0.1	e
T1_22	18.3	17.3	20.5±0.1	m
T1_23	19.3	12.92	18.3±0.1	g
T1_24	17.6	9.74	20.3±0.1	m
T1_25	18.2	14.55	17.1±0.1	e

T1_26	19.9	12.18	16.4±0.1	c
T1_27	18.8	14.50	15.5±0.1	a
T1_28	17.4	14.73	18.0±0.1	g
T1_29	18.3	10.26	18.3±0.1	g
T2_1	18.6	9.58	15.8±0.2	b
T2_2	19.5	15.29	19.3±0.1	i
T2_3	18.8	14.66	19.2±0.1	i
T2_4	18.4	9.73	21.8±0.2	p
T2_5	18	15.70	16.7±0.2	d
T2_6	18.6	12.73	16.4±0.1	c
T2_7	17.7	11.24	15.6±0.2	b
T2_8	20.2	20.41	17.7±0.1	f
T2_9	19.4	15.99	19.0±0.1	h
T2_10	19	8.80	16.9±0.2	d
T2_11	18.1	9.84	20.8±0.2	n
T2_12	19.7	14.43	17.0±0.1	e
T2_13	20.2	13.18	19.5±0.1	i
T2_14	16.1	8.60	21.9±0.2	p
T2_15	18.3	13.91	17.6±0.2	f
T2_16	19.2	15.48	19.4±0.1	i
T2_17	20.6	14.92	16.0±0.1	c
T2_18	19.1	14.04	21.6±0.2	p
T2_19	20.2	19.65	20.4±0.2	m
T2_20	19.5	16.71	18.1±0.1	g
T2_21	18.7	12.25	21.1±0.1	o
T2_22	19.5	15.85	18.1±0.1	g
T2_23	20.4	19.67	19.4±0.1	i
T2_24	19.4	18.46	17.4±0.1	e
T2_25	17.4	16.32	19.0±0.1	h
T2_26	18.3	10.76	16.7±0.2	d
T2_27	21.5	16.31	18.6±0.2	h
T2_28	18.8	10.64	21.2±0.1	o
T2_29	17.5	14.83	18.7±0.2	h
T2_30	19.5	15.26	19.1±0.1	i
T3_1	20.3	9.48	18.5±0.1	g
T3_2	18.5	11.18	17.4±0.1	e
T3_3	19	10.09	19.6±0.2	l
T3_4	19.3	15.76	19.2±0.1	i
T3_5	18.4	13.72	19.5±0.1	i
T3_6	18.8	17.80	19.0±0.1	h
T3_7	20.3	14.88	16.8±0.2	d

T3_8	19.5	11.03	17.0±0.1	e
T3_9	19.2	19.82	17.2±0.1	e
T3_10	18.6	9.72	15.2±0.1	a
T3_11	20.2	10.44	20.8±0.2	n
T3_12	17.9	8.79	18.3±0.1	g
T3_13	16.8	8.94	17.6±0.2	f
T3_14	19.6	17.32	19.3±0.1	i
T3_15	19.2	13.01	17.1±0.1	e
T3_16	16.9	10.30	19.6±0.2	l
T3_17	16.6	7.24	21.8±0.2	p
T3_18	18.2	9.64	18.5±0.1	g
T3_19	18.5	8.79	19.4±0.1	i
T3_20	19.9	13.13	17.4±0.1	e
T3_21	19.9	15.46	18.2±0.1	g
T3_22	19.2	10.3	19.7±0.2	l
T3_23	19.5	19.31	17.4±0.1	e
T3_24	18.4	9.55	19.8±0.2	l
T3_25	20.1	17.45	18.2±0.1	g
T3_26	18.6	13.56	17.1±0.1	e
T3_27	17.7	14.17	17.5±0.1	e
T3_28	19.2	13.23	17.2±0.1	e
T3_29	19.4	20.55	16.7±0.2	d
T3_30	18.7	15.76	17.4±0.1	e
**External test set**				
**Sample**	**DiamEqu**	**Hardness (N)**	**TSS (°Brix) ‡**	
T1_31	23	17.9	16.8±0.2	d
T1_32	17.6	9.5	17.6±0.2	f
T1_33	18.1	13	17.3±0.1	e
T2_31	19	8.7	16.8±0.2	d
T2_32	18.1	9.8	20.7±0.1	n
T2_33	19.7	14.4	17.1±0.1	e
T3_31	18.6	9.7	15.2±0.1	a
T3_32	20.2	10.3	20.7±0.2	n
T3_33	17.9	8.9	18.2±0.1	g

‡Mean ± SD. Different letters within the same column indicate a significant difference among samples (ANOVA test followed by a Tukey post hoc test using a p-value <0.05.).

Table S3. Regression coefficients, standard error, p-values and t-values for best iPLS on "hardness" parameter (ncomp = 4)

Interval	Wave numbers	Reg. coeffs	Std. err.	t-value	p-value (α= 0.05)
763	8496	-1.92969	1.038223	-1.85677	0.096307
764	8492	-1.7279	1.042464	-1.66088	0.131105
765	8488	-2.30822	0.985412	-2.35339	0.043065
766	8484	-2.73787	0.831196	-3.30815	0.009111
767	8480	-1.77608	0.64137	-2.77473	0.021586
768	8476	-1.15797	0.622962	-1.86047	0.095742
769	8472	-2.29818	0.722173	-3.19606	0.0109
770	8468	-3.57046	0.792756	-4.5235	0.00144
771	8464	-3.26976	0.806219	-4.06463	0.002822
772	8460	-2.4337	0.797087	-3.04862	0.013824
773	8456	-2.33325	0.529764	-4.39709	0.001728
774	8452	-2.32671	1.081519	-2.15311	0.059734
775	8448	-1.90051	1.501195	-1.26879	0.236348
776	8444	-1.16457	1.318182	-0.88586	0.398744
777	8440	-0.43217	1.010455	-0.42776	0.678881
778	8436	-0.2833	1.101428	-0.25266	0.806209
779	8432	-0.50941	1.24736	-0.40097	0.697792
780	8428	-0.85394	1.216783	-0.69489	0.504669
781	8424	-1.5892	0.932707	-1.70712	0.121982
782	8420	-1.99587	1.058893	-1.90033	0.08984
783	8416	-1.54263	1.108804	-1.40691	0.193035
784	8412	-0.91569	1.127246	-0.82244	0.432074
785	8408	-0.61618	1.235641	-0.5043	0.626168
786	8404	-0.77839	1.019338	-0.76595	0.463321
787	8400	-1.2748	0.732415	-1.73988	0.115873
788	8396	-1.49836	0.974507	-1.53773	0.158495
789	8392	-1.48603	1.169589	-1.27039	0.235803
790	8388	-1.68708	1.025575	-1.64206	0.134994
791	8384	-1.92725	1.106812	-1.73713	0.116375
792	8380	-2.31289	1.17648	-1.96526	0.080957
793	8376	-2.72368	1.012612	-2.69602	0.024553
794	8372	-2.30143	0.86828	-2.66136	0.025988
795	8368	-1.26664	0.829799	-1.53371	0.159469
796	8364	-0.7591	0.898771	-0.84916	0.417809
797	8360	-1.12471	0.933768	-1.21364	0.255773
798	8356	-1.83071	0.933915	-1.97279	0.079983

799	8352	-2.40616	0.881745	-2.74008	0.022845
800	8348	-2.65659	0.926394	-2.8775	0.018252
801	8344	-2.38336	1.103617	-2.16958	0.058154
802	8340	-1.83056	1.175908	-1.56767	0.151403
803	8336	-1.59672	1.002068	-1.60572	0.142799
804	8332	-1.5481	0.758937	-2.04982	0.070634
805	8328	-1.13332	0.654834	-1.73156	0.117398
806	8324	-0.69298	0.694251	-0.99157	0.347317
807	8320	-0.78175	0.552211	-1.4014	0.194623
808	8316	-0.99372	0.669331	-1.46828	0.176089
809	8312	-1.06133	0.720025	-1.46091	0.178054
810	8308	-1.35238	0.735195	-1.83709	0.09937
811	8304	-1.72811	0.844282	-2.04893	0.070736
812	8300	-1.76161	0.784584	-2.24133	0.051731
813	8296	-1.58033	0.604593	-2.60306	0.028595
814	8292	-1.47677	0.61388	-2.40222	0.039753
815	8288	-1.37535	0.678243	-2.03696	0.072119
816	8284	-1.07299	0.72834	-1.48584	0.171485
817	8280	-0.92513	0.991506	-0.93828	0.372596
818	8276	-1.0709	0.947551	-1.13137	0.287145
819	8272	-0.83009	0.624821	-1.32744	0.217049
820	8268	-0.59415	0.551897	-1.08059	0.307984
821	8264	-1.01163	0.674691	-1.51155	0.164937
822	8260	-1.19487	0.859614	-1.39366	0.196874
823	8256	-0.98081	0.837387	-1.1596	0.276048
824	8252	-1.0625	0.751379	-1.39706	0.195883
825	8248	-1.37621	0.813238	-1.68369	0.126529
826	8244	-1.92547	0.807684	-2.38439	0.040932
827	8240	-2.24907	0.848135	-2.65928	0.026077
828	8236	-1.95817	1.073818	-1.83328	0.099973
829	8232	-1.88136	1.010156	-1.87389	0.093716
830	8228	-2.00962	0.73071	-2.756	0.022257
831	8224	-1.77747	0.693849	-2.55484	0.030948
832	8220	-1.50678	0.652087	-2.30187	0.046856
833	8216	-1.191	0.586108	-2.02421	0.073619
834	8212	-0.77949	0.747544	-1.03382	0.3282
835	8208	-1.24626	0.639969	-1.94213	0.084022
836	8204	-2.42943	0.682287	-3.56964	0.006028
837	8200	-2.84381	0.735204	-3.87915	0.003736
838	8196	-2.20072	0.652807	-3.37463	0.008196
839	8192	-1.42127	0.651387	-2.17812	0.05735

840	8188	-1.28375	0.748134	-1.71097	0.121249
841	8184	-1.55966	0.739685	-2.10621	0.064466
842	8180	-1.98766	0.746253	-2.66721	0.02574
843	8176	-2.89073	0.664217	-4.36452	0.001811
844	8172	-3.72059	0.783485	-4.76453	0.001023
845	8168	-3.4001	0.827926	-4.12104	0.002594
846	8164	-2.3363	0.83618	-2.80496	0.020546
847	8160	-1.7804	0.91126	-1.9651	0.080977
848	8156	-2.17653	1.001384	-2.19011	0.05624
849	8152	-2.92489	1.009982	-2.91679	0.017121
850	8148	-3.15241	0.732171	-4.32885	0.001908
851	8144	-2.77337	0.765488	-3.63609	0.005434
852	8140	-2.51501	0.973798	-2.59149	0.029142
853	8136	-2.73159	0.860107	-3.18822	0.011038
854	8132	-3.183	0.736813	-4.33414	0.001894
855	8128	-3.54502	0.783476	-4.5381	0.00141
856	8124	-3.35975	0.820444	-4.10746	0.002647
857	8120	-2.46666	0.665921	-3.70982	0.004846
858	8116	-1.69238	0.591526	-2.85917	0.018806
859	8112	-1.65687	0.65208	-2.54914	0.031239
860	8108	-1.79535	0.646811	-2.79567	0.02086
861	8104	-1.61552	0.517309	-3.14399	0.011852
862	8100	-1.35753	0.630877	-2.15672	0.059384
863	8096	-1.26651	0.730999	-1.7244	0.118723
864	8092	-1.38282	0.697745	-1.96446	0.081061
865	8088	-1.69066	0.586195	-2.86708	0.018565
866	8084	-1.95715	0.450679	-4.33669	0.001887
867	8080	-1.74815	0.607394	-2.8801	0.018175
868	8076	-1.23228	0.804682	-1.53107	0.160111
869	8072	-1.27497	0.678244	-1.87976	0.092841
870	8068	-2.15867	0.502011	-4.31527	0.001947
871	8064	-3.03196	0.680767	-4.47814	0.001537
872	8060	-3.27578	0.816249	-4.03651	0.002944
873	8056	-3.14364	0.88332	-3.57836	0.005946
874	8052	-2.84713	0.852518	-3.35654	0.008435
875	8048	-2.33908	0.769452	-3.05079	0.013775
876	8044	-1.90133	0.713013	-2.66735	0.025734
877	8040	-2.10696	0.543517	-3.8735	0.003769
878	8036	-2.6022	0.499017	-5.22166	0.000548
879	8032	-2.57567	0.653528	-3.95565	0.003326
880	8028	-2.2217	0.660466	-3.37976	0.00813

881	8024	-2.19373	0.620425	-3.55072	0.006209
882	8020	-2.63966	0.634414	-4.17654	0.002388
883	8016	-3.0682	0.650753	-4.73079	0.001073
884	8012	-2.94048	0.612681	-4.80902	0.000962
885	8008	-2.56399	0.694234	-3.69227	0.00498
886	8004	-2.26845	0.511029	-4.43062	0.001646
887	8000	-1.76767	0.617556	-2.85116	0.019053
888	7996	-1.56665	0.851088	-1.83207	0.100166
889	7992	-2.0572	0.938839	-2.18863	0.056376
890	7988	-2.23582	0.929051	-2.40858	0.039341
891	7984	-2.05906	0.88733	-2.32639	0.045012
892	7980	-2.33022	0.846835	-2.76466	0.021944
893	7976	-2.55555	0.813697	-3.15608	0.011623
894	7972	-2.11138	0.675863	-3.13456	0.012033
895	7968	-1.45194	0.532332	-2.72949	0.023244
896	7964	-1.27711	0.509768	-2.50328	0.03368
897	7960	-1.63916	0.651933	-2.51604	0.032982
898	7956	-2.05182	0.73463	-2.79961	0.020726
899	7952	-2.20042	0.708281	-3.11549	0.012409
900	7948	-1.93943	0.65432	-2.96681	0.015784
901	7944	-1.48298	0.862139	-1.71648	0.120208
902	7940	-1.45524	0.899874	-1.6173	0.140268
903	7936	-1.91962	0.722948	-2.6657	0.025803
904	7932	-2.47029	0.641729	-3.86507	0.003817
905	7928	-2.72349	0.739075	-3.69413	0.004965
906	7924	-2.55077	0.791602	-3.22172	0.01046
907	7920	-2.24525	0.774499	-2.88919	0.017908
908	7916	-2.04468	0.562283	-3.62557	0.005524
909	7912	-2.15521	0.458888	-4.7099	0.001105
910	7908	-2.77051	0.577643	-4.82857	0.000936
911	7904	-3.33761	0.771503	-4.35323	0.001842
912	7900	-3.24144	0.799561	-4.07128	0.002794
913	7896	-2.77939	0.811873	-3.42993	0.007509
914	7892	-2.3756	0.793182	-2.99422	0.015097
915	7888	-2.04162	0.726296	-2.80775	0.020452
916	7884	-1.84777	0.70832	-2.61115	0.028217
917	7880	-1.90725	0.649048	-2.95134	0.016186
918	7876	-2.09194	0.740029	-2.84446	0.019263
919	7872	-2.03611	0.92919	-2.20497	0.054894
920	7868	-1.74108	0.999933	-1.74654	0.114666
921	7864	-1.96926	0.898264	-2.19183	0.056083

922	7860	-2.61853	0.764745	-3.42709	0.007543
923	7856	-2.82576	0.779376	-3.63498	0.005443
924	7852	-2.69005	1.013829	-2.66527	0.025822
925	7848	-2.40607	1.099407	-2.20265	0.055102
926	7844	-1.97654	0.913491	-2.17815	0.057347
927	7840	-1.78963	0.73181	-2.46189	0.036047
928	7836	-1.9928	0.626163	-3.2075	0.010702
929	7832	-2.17672	0.556266	-3.94172	0.003397
930	7828	-2.09587	0.702134	-3.00413	0.014857
931	7824	-2.14955	0.767016	-2.81959	0.02006
932	7820	-2.49484	0.792023	-3.16568	0.011445
933	7816	-2.75733	0.855293	-3.23777	0.010195
934	7812	-2.77373	0.845628	-3.2945	0.009311
935	7808	-2.61459	0.69434	-3.78128	0.00434
936	7804	-2.37073	0.494482	-4.81838	0.000949
937	7800	-2.20275	0.37504	-5.9119	0.000226
938	7796	-2.16973	0.407039	-5.36045	0.000456
939	7792	-2.15851	0.470708	-4.599	0.001292
940	7788	-2.05954	0.497513	-4.1446	0.002504
941	7784	-2.01698	0.514402	-3.92578	0.00348
942	7780	-2.07229	0.707442	-2.93579	0.0166
943	7776	-2.08771	0.943128	-2.22136	0.053446
944	7772	-1.95971	0.992228	-1.98158	0.078858
945	7768	-1.54204	0.981985	-1.56881	0.151139
946	7764	-1.10574	0.86924	-1.2623	0.238567
947	7760	-1.02481	0.575261	-1.77224	0.110117
948	7756	-1.00518	0.494854	-2.03369	0.072501
949	7752	-0.84649	0.566366	-1.49885	0.168143
950	7748	-1.02837	0.550429	-1.87414	0.093679
951	7744	-1.68639	0.47477	-3.57014	0.006023
952	7740	-2.21177	0.661781	-3.36224	0.008359
953	7736	-2.20926	0.623989	-3.55988	0.00612
954	7732	-1.98773	0.538922	-3.70542	0.004879
955	7728	-1.91076	0.674497	-2.84629	0.019205
956	7724	-1.89951	0.794487	-2.40167	0.039789
957	7720	-1.64559	0.765031	-2.1572	0.059337
958	7716	-0.91883	0.517115	-1.77266	0.110044
959	7712	-0.13969	0.309603	-0.42534	0.680578
960	7708	-0.22494	0.491465	-0.44567	0.666364
961	7704	-1.06373	0.631912	-1.68876	0.125532
962	7700	-1.63482	0.668496	-2.46286	0.03599

963	7696	-1.70329	0.859672	-1.99776	0.07683
964	7692	-1.69345	0.881356	-1.93593	0.084861
965	7688	-1.72209	0.715489	-2.42325	0.038405
966	7684	-1.7161	0.542059	-3.1903	0.011001
967	7680	-1.5473	0.465971	-3.35282	0.008485
968	7676	-1.19782	0.435707	-2.7829	0.0213
969	7672	-0.8722	0.443056	-1.99781	0.076824
970	7668	-0.77552	0.606401	-1.30064	0.225695
971	7664	-1.1331	0.653513	-1.76324	0.11169
972	7660	-1.59442	0.565947	-2.86117	0.018745
973	7656	-1.26062	0.410283	-3.11981	0.012322
974	7652	-0.31795	0.315824	-1.03408	0.328084
975	7648	0.382823	0.50411	0.752825	0.470787
976	7644	0.885492	0.841742	1.051064	0.320631
977	7640	1.04019	0.876529	1.187594	0.26539
978	7636	0.570925	0.848004	0.672924	0.517899
979	7632	0.158054	0.935823	0.167529	0.870658
980	7628	0.309214	0.799918	0.385388	0.708901
981	7624	0.865352	0.603727	1.433968	0.185397
982	7620	1.430247	0.64291	2.227282	0.052931
983	7616	1.502841	0.632752	2.374791	0.041581
984	7612	1.205068	0.488279	2.462665	0.036001
985	7608	1.299613	0.549782	2.370478	0.041876
986	7604	1.644564	0.779055	2.130314	0.06199
987	7600	1.819876	0.935421	1.964935	0.080999
988	7596	1.736324	0.644508	2.709135	0.024032
989	7592	1.024957	0.370664	2.762225	0.022032
990	7588	0.566385	0.576048	0.974695	0.35518
991	7584	1.354346	0.316205	4.277189	0.002058
992	7580	2.552352	0.40201	6.353415	0.000132
993	7576	3.051035	0.633728	4.818882	0.000948
994	7572	2.798721	0.681484	4.109134	0.00264
995	7568	2.535269	0.732186	3.464318	0.007112
996	7564	2.570126	0.673005	3.821923	0.004078
997	7560	2.655008	0.750103	3.542102	0.006293
998	7556	2.740136	0.877328	3.12642	0.012192
999	7552	2.835244	0.817126	3.47574	0.006985
1000	7548	3.306342	0.74204	4.46616	0.001563
1001	7544	4.070374	0.684466	5.961048	0.000212
1002	7540	4.262638	0.628112	6.797419	7.93E-05
1003	7536	3.939882	0.732235	5.383473	0.000442

1004	7532	3.62262	0.840277	4.31122	0.001958
1005	7528	3.324411	0.888217	3.74212	0.00461
1006	7524	3.231004	0.862951	3.744519	0.004593
1007	7520	3.356857	0.762031	4.407755	0.001701
1008	7516	3.384806	0.717351	4.721475	0.001087
1009	7512	3.531223	0.689544	5.129769	0.00062
1010	7508	3.955162	0.999022	3.972352	0.003243
1011	7504	4.302835	1.085199	3.978978	0.003211
1012	7500	4.601388	0.98417	4.689345	0.001137
1013	7496	4.862741	1.066592	4.5731	0.001341
1014	7492	4.800517	1.036477	4.646283	0.001208
1015	7488	4.890555	0.919638	5.334913	0.000472
1016	7484	5.533132	0.984925	5.638095	0.000318
1144	**6972**	-3.84789	1.081782	-3.56487	0.006073
1145	6968	-4.01187	1.219901	-3.30205	0.0092
1146	6964	-4.32045	1.100951	-3.9391	0.003411
1147	6960	-4.51829	1.157484	-3.91097	0.003559
1148	6956	-4.27349	1.153426	-3.70683	0.004869
1149	6952	-4.10606	0.887108	-4.62859	0.001239
1150	6948	-4.33765	0.931997	-4.65776	0.001189
1151	6944	-4.43743	1.08953	-4.08065	0.002755
1152	6940	-4.43828	1.019766	-4.36092	0.001821
1153	6936	-4.53254	0.905506	-5.01348	0.000726
1154	6932	-4.2791	0.703632	-6.09053	0.000181
1155	6928	-3.77615	0.467432	-8.08786	2.03E-05
1156	6924	-3.84759	0.61899	-6.22655	0.000154
1157	6920	-4.36768	0.968871	-4.51737	0.001452
1158	6916	-4.54809	0.973387	-4.67599	0.001159
1159	6912	-4.41999	0.797533	-5.54226	0.00036
1160	6908	-4.45638	0.665136	-6.70791	8.77E-05
1161	6904	-4.53243	0.634831	-7.1512	5.36E-05
1162	6900	-4.17388	0.734594	-5.68484	0.0003
1163	6896	-3.57845	0.771028	-4.63863	0.001222
1164	6892	-3.43842	0.839939	-4.09331	0.002704
1165	6888	-3.72504	0.865707	-4.30744	0.001969
1166	6884	-3.85656	0.896604	-4.30932	0.001964
1167	6880	-3.80891	0.784882	-4.86499	0.00089
1168	6876	-3.87513	0.794064	-4.89257	0.000856
1169	6872	-3.94404	0.789286	-5.00322	0.000736
1170	6868	-3.79747	0.848996	-4.47021	0.001554
1171	6864	-3.42139	1.067538	-3.20074	0.010818

1172	6860	-2.99475	1.125662	-2.6587	0.026101
1173	6856	-2.93869	0.945757	-3.10849	0.012549
1174	6852	-3.20369	0.716055	-4.47718	0.001539
1175	6848	-3.28889	0.792545	-4.15181	0.002478
1176	6844	-3.2788	0.916485	-3.58265	0.005906
1177	6840	-3.35889	0.927613	-3.63197	0.005469
1178	6836	-3.56987	1.037158	-3.4542	0.007227
1179	6832	-3.87184	1.167742	-3.32579	0.008858
1180	6828	-3.8049	1.067701	-3.57213	0.006004
1181	6824	-3.54695	0.892447	-3.98331	0.00319
1182	6820	-3.39931	0.801126	-4.25357	0.002131
1183	6816	-3.20452	0.82083	-3.9118	0.003555
1184	6812	-3.27888	0.893578	-3.67158	0.005142
1185	6808	-3.52647	0.810211	-4.35096	0.001848
1186	6804	-3.14147	0.71167	-4.41193	0.001691
1187	6800	-2.49168	0.661576	-3.76243	0.004468
1188	6796	-2.33962	0.711962	-3.27999	0.009529
1189	6792	-2.16873	0.830948	-2.60238	0.028626
1190	6788	-1.7534	0.735319	-2.36856	0.042008
1191	6784	-1.61968	0.708533	-2.26327	0.049909
1192	6780	-1.93106	0.776621	-2.4733	0.035378
1193	6776	-2.36646	0.881492	-2.68312	0.025078
1194	6772	-2.6533	0.871644	-3.04657	0.01387
1195	6768	-3.1042	0.825162	-3.76871	0.004425
1196	6764	-3.58069	0.868869	-4.1316	0.002553
1197	6760	-3.61738	0.940027	-3.85934	0.003851
1198	6756	-3.29186	0.937424	-3.52391	0.006476
1199	6752	-2.86594	0.838657	-3.4293	0.007517
1200	6748	-2.52301	0.867133	-2.91862	0.01707
1201	6744	-2.12637	0.74087	-2.88086	0.018152
1202	6740	-1.80931	0.582111	-3.1273	0.012175
1203	6736	-1.88314	0.688819	-2.75702	0.02222
1204	6732	-2.16182	0.739761	-2.9428	0.016412
1205	6728	-2.40142	0.501837	-4.80197	0.000971
1206	6724	-2.35658	0.591353	-3.99071	0.003154
1207	6720	-2.08601	0.832106	-2.50952	0.033337
1208	6716	-2.30506	1.060016	-2.17958	0.057213
1209	6712	-2.96758	1.156941	-2.57549	0.029917
1210	6708	-3.03019	0.990911	-3.07403	0.013267
1211	6704	-2.49944	0.785873	-3.19813	0.010864
1212	6700	-2.28646	0.710066	-3.23607	0.010223

1213	6696	-2.36663	0.610793	-3.88935	0.003679
1214	6692	-2.05318	0.516567	-3.97981	0.003207
1215	6688	-1.29554	0.582901	-2.21506	0.053998
1216	6684	-0.81919	0.714243	-1.13955	0.283892
1217	6680	-1.00873	0.809194	-1.24723	0.243792
1218	6676	-1.2164	0.76716	-1.59623	0.144901
1219	6672	-1.12208	0.863701	-1.313	0.221672
1220	6668	-1.21754	0.89405	-1.37055	0.203724
1221	6664	-1.32966	0.801733	-1.6571	0.131878
1222	6660	-1.01069	0.804594	-1.24808	0.243494
1223	6656	-0.67427	0.72744	-0.91908	0.382026
1224	6652	-0.5797	0.717473	-0.8035	0.442387
1225	6648	-0.38417	0.788659	-0.48317	0.640509
1226	6644	-0.27176	0.779493	-0.34553	0.737634
1227	6640	-0.68998	0.690187	-1.00782	0.339866
1228	6636	-1.1922	0.638365	-1.89513	0.090591
1229	6632	-1.2715	0.665157	-1.94778	0.083264
1230	6628	-1.12814	0.689541	-1.66966	0.129327
1231	6624	-0.86155	0.657136	-1.33795	0.213734
1232	6620	-0.64401	0.548327	-1.19912	0.261098
1233	6616	-0.44485	0.444654	-1.02431	0.332432
1234	6612	0.096272	0.286895	0.32908	0.749624
1235	6608	0.669766	0.324178	2.08727	0.066477
1236	6604	0.673867	0.459598	1.477915	0.17355
1237	6600	0.308517	0.698317	0.440859	0.669715
1238	6596	0.437016	0.661188	0.661389	0.524932
1239	6592	0.972507	0.446471	2.190177	0.056234
1240	6588	0.987827	0.547616	1.810293	0.103687
1241	6584	0.654325	0.693311	0.938999	0.372246
1242	6580	0.636546	0.612428	1.025976	0.331687
1243	6576	0.806779	0.540324	1.472961	0.174852
1244	6572	0.781011	0.577643	1.333507	0.21513
1245	6568	0.837953	0.693031	1.200759	0.260493
1246	6564	1.239881	0.678188	1.830382	0.100435
1247	6560	1.31609	0.640743	2.058826	0.069612
1248	6556	0.86162	0.584573	1.472607	0.174945
1249	6552	0.549866	0.525756	1.040387	0.325301
1250	6548	0.860372	0.548595	1.5667	0.151627
1251	6544	1.587942	0.600488	2.653377	0.02633
1252	6540	1.902632	0.572073	3.341172	0.008644
1253	6536	1.691858	0.589438	2.876283	0.018288

1254	6532	1.706414	0.602295	2.834334	0.019583
1255	6528	2.105575	0.619508	3.411525	0.007731
1256	6524	2.469102	0.721258	3.4454	0.007328
1257	6520	2.627967	0.732336	3.61165	0.005645
1258	6516	2.641472	0.650329	4.082218	0.002749
1259	6512	2.738048	0.658827	4.171252	0.002407
1260	6508	3.062343	0.676865	4.536873	0.001412
1261	6504	3.29782	0.622698	5.307229	0.000489
1262	6500	3.321378	0.723413	4.601094	0.001289
1263	6496	3.44874	0.919145	3.761616	0.004474
1264	6492	3.588018	0.944524	3.8064	0.004176
1265	6488	3.443395	0.647542	5.321734	0.00048
1266	6484	3.207404	0.425193	7.54195	3.53E-05
1267	6480	3.264618	0.584234	5.589004	0.000339
1268	6476	3.588278	0.751227	4.787048	0.000991
1269	**6472**	3.609944	0.923857	3.9234	0.003493
1522	5460	6.339926	0.880183	7.211706	5.02E-05
1523	5456	6.32354	0.835673	7.573959	3.42E-05
1524	5452	6.430552	0.837762	7.683966	3.05E-05
1525	5448	6.438968	0.804865	8.008921	2.19E-05
1526	5444	6.145425	0.704735	8.727394	1.10E-05
1527	5440	5.728312	0.612365	9.360573	6.18E-06
1528	5436	5.419111	0.662282	8.188643	1.84E-05
1529	5432	5.258606	0.853638	6.164328	0.000166
1530	5428	5.240663	0.972553	5.390214	0.000439
1531	5424	5.197142	0.91598	5.673771	0.000304
1532	5420	5.120583	0.78656	6.50895	0.00011
1533	5416	5.203051	0.690724	7.530646	3.58E-05
1534	5412	5.271927	0.654622	8.050756	2.10E-05
1535	5408	5.206307	0.637727	8.163119	1.88E-05
1536	5404	5.16083	0.737618	6.999934	6.33E-05
1537	5400	5.070871	0.912811	5.560929	0.000351
1538	5396	4.819312	0.896213	5.382948	0.000443
1539	5392	4.466351	0.714679	6.251023	0.000149
1540	5388	4.17423	0.563412	7.401672	4.10E-05
1541	5384	4.015121	0.558742	7.170475	5.25E-05
1542	5380	3.84112	0.562218	6.812165	7.80E-05
1543	5376	3.46907	0.611535	5.654947	0.000312
1544	5372	3.064767	0.687017	4.446653	0.001608
1545	5368	2.658196	0.742785	3.56426	0.006079
1546	5364	2.339714	0.766854	3.037387	0.014077

1547	5360	2.367026	0.733062	3.21594	0.010558
1548	5356	2.279178	0.645523	3.511126	0.006607
1549	5352	1.748461	0.648458	2.673076	0.025494
1550	5348	1.099339	0.791438	1.371939	0.203307
1551	5344	0.426036	0.987902	0.416892	0.686525
1552	5340	-0.21636	1.074898	-0.22055	0.830362
1553	5336	-0.67528	1.172965	-0.59959	0.563571
1554	5332	-1.10013	1.133403	-0.99499	0.345738
1555	5328	-1.44667	1.07104	-1.37043	0.203761
1556	5324	-1.53649	1.160441	-1.33933	0.213302
1557	5320	-1.68548	1.268237	-1.3442	0.211782
1558	5316	-2.14038	1.310496	-1.64823	0.133707
1559	5312	-2.63372	1.289763	-2.055	0.070045
1560	5308	-2.67019	1.230792	-2.18064	0.057115
1561	5304	-2.30028	1.143791	-2.02157	0.073935
1562	5300	-2.06088	1.039254	-1.99233	0.077505
1563	5296	-2.11457	1.042172	-2.03564	0.072273
1564	5292	-2.19842	1.206027	-1.82899	0.100657
1565	5288	-2.129	1.314197	-1.62773	0.138025
1566	5284	-1.92223	1.267941	-1.52367	0.161925
1567	5280	-1.59046	1.13061	-1.41247	0.191443
1568	5276	-1.09822	0.952756	-1.15678	0.277144
1569	5272	-0.51671	0.819797	-0.6332	0.542356
1570	5268	-0.04959	0.768737	-0.06762	0.94757
1571	5264	0.220449	0.809927	0.266326	0.795989
1572	5260	0.575453	0.878775	0.646243	0.534252
1573	5256	1.25675	0.733488	1.70414	0.122552
1574	5252	2.080449	0.496347	4.185076	0.002358
1575	5248	2.759037	0.499809	5.517684	0.000372
1576	5244	3.166959	0.683629	4.632711	0.001232
1577	5240	3.236761	0.740551	4.376394	0.00178
1578	5236	3.101765	0.678036	4.587379	0.001314
1579	5232	2.949148	0.613328	4.824015	0.000942
1580	5228	2.843561	0.48864	5.834123	0.000249
1581	5224	2.951027	0.507583	5.823938	0.000252
1582	5220	3.376535	0.587602	5.756005	0.000274
1583	5216	3.959792	0.498551	7.958152	2.31E-05
1584	5212	4.377423	0.270635	16.19554	5.79E-08
1585	5208	4.292973	0.401654	10.68038	2.06E-06
1586	5204	3.914226	0.56902	6.860108	7.39E-05
1587	5200	3.724708	0.529199	7.018062	6.20E-05

1588	5196	3.695842	0.630947	5.843907	0.000246
1589	5192	3.657498	0.76432	4.77502	0.001008
1590	5188	3.647527	0.799895	4.551757	0.001382
1591	5184	3.659325	0.880934	4.150852	0.002481
1592	5180	3.607714	0.94936	3.8031	0.004197
1593	5176	3.515883	0.889891	3.955981	0.003324
1594	5172	3.518392	1.019441	3.455165	0.007216
1595	5168	3.6051	0.972787	3.70909	0.004852
1596	5164	3.689789	0.758975	4.861453	0.000894
1597	5160	3.645237	0.753298	4.834939	0.000928
1598	5156	3.424961	0.837003	4.086821	0.00273
1599	5152	3.619431	0.727333	4.976882	0.000763
1600	5148	4.398353	0.628728	7.009535	6.26E-05
1601	5144	4.755318	0.640427	7.440874	3.93E-05
1602	5140	4.327308	0.665538	6.513942	0.00011
1603	5136	3.803143	0.744782	5.121688	0.000627
1604	5132	3.681047	0.718374	5.144543	0.000608
1605	5128	3.516528	0.583538	6.043134	0.000192
1606	5124	2.927075	0.534768	5.478295	0.000391
1607	5120	2.4073	0.608417	3.958329	0.003313
1608	5116	2.09324	0.72148	2.904222	0.017475
1609	5112	1.860679	0.73438	2.533748	0.032038
1610	5108	1.93738	0.604638	3.200621	0.010821
1611	5104	2.170586	0.533033	4.069288	0.002803
1612	5100	2.06266	0.580018	3.553557	0.006181
1613	5096	1.741361	0.559599	3.106249	0.012595
1614	5092	1.714157	0.419025	4.084993	0.002737
1615	5088	1.868671	0.396298	4.717106	0.001094
1616	5084	1.874443	0.473721	3.96348	0.003287
1617	5080	1.683551	0.515386	3.272726	0.009641
1618	5076	1.398586	0.463928	3.019182	0.014499
1619	5072	1.159249	0.449267	2.584648	0.029471
1620	5068	0.963528	0.556083	1.734888	0.116785
1621	5064	0.763048	0.698424	1.093064	0.302758
1622	5060	0.655985	0.825168	0.798314	0.445242
1623	5056	0.637675	0.796374	0.807751	0.44006
1624	5052	0.58749	0.618675	0.957194	0.363474
1625	5048	0.537588	0.601932	0.89744	0.392858
1626	5044	0.464065	0.629577	0.739531	0.478429
1627	5040	0.303488	0.634078	0.481044	0.641964
1628	5036	0.060831	0.684346	0.094974	0.926416

1629	5032	-0.28168	0.715198	-0.38333	0.710369
1630	5028	-0.59803	0.671148	-0.88058	0.401448
1631	5024	-0.63768	0.568488	-1.11843	0.292344
1632	5020	-0.49799	0.541143	-0.92942	0.376927
1633	5016	-0.55055	0.556552	-1.0038	0.3417
1634	5012	-0.82171	0.543778	-1.51981	0.16288
1635	5008	-0.9007	0.568248	-1.58234	0.14803
1636	5004	-0.79012	0.71317	-1.10063	0.299623
1637	5000	-0.99545	0.885363	-1.12084	0.29137
1638	4996	-1.48538	0.903929	-1.64649	0.134069
1639	4992	-1.75417	0.826164	-2.13074	0.061947
1640	4988	-1.89402	0.769678	-2.46702	0.035745
1641	4984	-2.0923	0.762976	-2.74587	0.022629
1642	4980	-2.08453	0.715071	-2.91729	0.017107
1643	4976	-1.79316	0.628506	-2.85047	0.019075
1644	4972	-1.42757	0.590264	-2.40802	0.039377
1645	4968	-1.35619	0.592026	-2.28175	0.048424
1646	4964	-1.90576	0.565378	-3.37313	0.008216
1647	4960	-2.81545	0.643263	-4.38922	0.001748
1648	4956	-3.26214	0.746449	-4.38547	0.001757
1649	4952	-3.04166	0.775806	-3.93371	0.003439
1650	4948	-2.80447	0.780714	-3.59844	0.005762
1651	4944	-2.95511	0.835477	-3.53656	0.006348
1652	4940	-3.47393	0.767257	-4.5281	0.00143
1653	4936	-3.93334	0.652286	-6.03946	0.000193
1654	4932	-4.0759	0.681863	-5.99358	0.000204
1655	4928	-4.20687	0.806623	-5.23161	0.000541
1656	4924	-4.29631	0.873178	-4.93489	0.000808
1657	4920	-4.18586	0.915635	-4.58436	0.00132
1658	4916	-4.09218	0.956186	-4.29304	0.002011
1659	4912	-4.1682	1.002833	-4.17063	0.002409
1660	4908	-4.28815	1.040109	-4.1372	0.002532
1661	4904	-4.19675	0.984356	-4.27843	0.002055
1662	4900	-3.84336	0.89167	-4.32319	0.001924
1663	4896	-3.58508	0.721818	-4.97555	0.000764
1664	4892	-3.84717	0.558776	-6.8963	7.10E-05
1665	4888	-4.39282	0.659269	-6.68316	9.03E-05
1666	4884	-4.59881	0.831079	-5.55646	0.000353
1667	4880	-4.33895	0.804842	-5.41347	0.000425
1668	4876	-3.94457	0.6256	-6.32358	0.000137
1669	4872	-3.87607	0.502649	-7.72362	2.93E-05

1670	4868	-4.16869	0.500553	-8.34497	1.58E-05
1671	4864	-4.22449	0.569303	-7.44516	3.91E-05
1672	4860	-3.94521	0.586979	-6.74759	8.39E-05
1673	4856	-3.8677	0.58821	-6.59683	9.96E-05
1674	4852	-4.15796	0.613396	-6.79475	7.95E-05
1675	4848	-4.48085	0.658029	-6.82395	7.70E-05
1676	4844	-4.48648	0.715317	-6.28614	0.000143
1677	4840	-4.22988	0.705573	-6.00863	0.0002
1678	4836	-4.03542	0.643861	-6.28056	0.000144
1679	4832	-4.02768	0.616185	-6.54936	0.000105
1680	4828	-4.19085	0.661142	-6.35372	0.000132
1681	4824	-4.4107	0.774487	-5.71107	0.00029
1682	4820	-4.43439	0.762597	-5.8287	0.00025
1683	4816	-4.34959	0.613735	-7.09703	5.69E-05
1684	4812	-4.37871	0.569617	-7.69975	3.00E-05
1685	4808	-4.36392	0.728713	-6.00567	0.000201
1686	4804	-4.20512	0.850362	-4.96115	0.000779
1687	4800	-4.1672	0.825133	-5.06268	0.000679
1688	4796	-4.40206	0.787613	-5.59964	0.000334
1689	4792	-4.80842	0.79888	-6.03269	0.000195
1690	4788	-5.12795	0.818363	-6.28517	0.000143
1691	4784	-5.15729	0.802909	-6.4452	0.000119
1692	4780	-4.92752	0.696316	-7.09785	5.68E-05
1693	4776	-4.72492	0.602873	-7.85634	2.56E-05
1694	4772	-4.85517	0.633057	-7.68951	3.03E-05
1695	4768	-5.06309	0.68204	-7.44609	3.91E-05
1696	4764	-4.84309	0.735932	-6.60191	9.91E-05
1697	4760	-4.35227	0.74096	-5.892	0.000231
1698	4756	-4.1913	0.68143	-6.16763	0.000165
1699	4752	-4.40923	0.674754	-6.55323	0.000105
1700	4748	-4.56659	0.704738	-6.50152	0.000111
1701	4744	-4.53222	0.75516	-6.02018	0.000198
1702	4740	-4.43426	0.732035	-6.07074	0.000186
1703	4736	-4.28732	0.659957	-6.50838	0.00011
1704	4732	-4.27034	0.697338	-6.13678	0.000171
1705	4728	-4.5682	0.685494	-6.68108	9.05E-05
1706	4724	-4.88516	0.647347	-7.56923	3.43E-05
1707	4720	-4.86813	0.606551	-8.05034	2.11E-05
1708	4716	-4.6944	0.568685	-8.27639	1.69E-05
1709	4712	-4.60307	0.645506	-7.149	5.37E-05
1710	4708	-4.5674	0.713225	-6.42024	0.000122

1711	4704	-4.54606	0.656591	-6.94046	6.76E-05
1712	4700	-4.45457	0.587212	-7.60482	3.31E-05
1713	4696	-4.3282	0.605093	-7.17225	5.24E-05
1714	4692	-4.25799	0.688421	-6.20139	0.000159
1715	4688	-4.30009	0.743527	-5.79538	0.000261
1716	4684	-4.51906	0.735546	-6.15368	0.000168
1717	4680	-4.77558	0.739127	-6.4725	0.000115
1718	4676	-4.87989	0.771244	-6.34167	0.000134
1719	4672	-4.74234	0.737201	-6.44891	0.000118
1720	4668	-4.50913	0.696487	-6.4893	0.000113
1721	4664	-4.3799	0.609719	-7.1991	5.09E-05
1722	4660	-4.31505	0.52496	-8.23606	1.75E-05
1723	4656	-4.26522	0.484768	-8.81447	1.01E-05
1724	4652	-4.1647	0.454479	-9.18228	7.24E-06
1725	4648	-3.97883	0.533298	-7.4806	3.77E-05
1726	4644	-3.82246	0.617935	-6.20298	0.000158
1727	4640	-3.70226	0.613408	-6.04754	0.000191
1728	4636	-3.61217	0.627732	-5.76276	0.000272
1729	4632	-3.61837	0.689295	-5.25957	0.000521
1730	4628	-3.67325	0.776803	-4.74194	0.001056
1731	4624	-3.67559	0.781641	-4.71638	0.001095
1732	4620	-3.60466	0.658875	-5.48122	0.00039
1733	4616	-3.62192	0.518884	-6.98629	6.42E-05
1734	4612	-3.94936	0.445669	-8.87672	9.56E-06
1735	4608	-4.35894	0.577745	-7.56881	3.44E-05
1736	4604	-4.36971	0.709431	-6.18388	0.000162
1737	4600	-4.014	0.763929	-5.27578	0.00051
1738	4596	-3.76009	0.740374	-5.09818	0.000647
1739	4592	-3.8945	0.747116	-5.23211	0.00054
1740	4588	-4.04993	0.739557	-5.49545	0.000382
1741	4584	-3.76923	0.640476	-5.90366	0.000228
1742	4580	-3.30688	0.593675	-5.58734	0.00034
1743	4576	-3.2146	0.565452	-5.7037	0.000293
1744	4572	-3.49052	0.536949	-6.52201	0.000109
1745	4568	-3.75823	0.558415	-6.75143	8.35E-05
1746	4564	-3.97736	0.533114	-7.48594	3.75E-05
1747	4560	-4.1177	0.632596	-6.534	0.000107
1748	4556	-3.82256	0.69676	-5.50593	0.000377
1749	4552	-3.21169	0.544359	-5.91511	0.000225
1750	4548	-3.03481	0.435526	-6.98056	6.46E-05
1751	4544	-3.35344	0.458066	-7.33566	4.39E-05

1752	4540	-3.57758	0.502888	-7.13149	5.48E-05
1753	4536	-3.59003	0.441798	-8.14773	1.91E-05
1754	4532	-3.52409	0.432871	-8.16598	1.88E-05
1755	4528	-3.37422	0.562981	-6.01515	0.000199
1756	4524	-3.17505	0.648955	-4.91151	0.000834
1757	4520	-3.11128	0.638621	-4.89156	0.000858
1758	4516	-3.37254	0.672389	-5.03821	0.000701
1759	4512	-3.74069	0.756667	-4.96572	0.000774
1760	4508	-3.76255	0.770213	-4.90351	0.000844
1761	4504	-3.50219	0.687773	-5.10697	0.000639
1762	4500	-3.3259	0.608078	-5.48654	0.000387
1763	4496	-3.45549	0.62176	-5.5809	0.000343
1764	4492	-3.87169	0.70162	-5.54222	0.00036
1765	4488	-4.10829	0.637153	-6.47099	0.000115
1766	4484	-3.72443	0.56512	-6.60925	9.82E-05
1767	4480	-3.11136	0.563251	-5.53492	0.000363
1768	4476	-2.99304	0.60707	-4.93652	0.000806
1769	4472	-3.14067	0.610694	-5.1507	0.000603
1770	4468	-3.03638	0.547892	-5.55242	0.000355
1771	4464	-2.90805	0.440182	-6.61782	9.73E-05
1772	4460	-3.05296	0.542256	-5.64168	0.000317
1773	4456	-3.32109	0.729259	-4.5645	0.001358
